# Tackling algorithmic bias and promoting transparency in health datasets: the STANDING Together consensus recommendations

**DOI:** 10.1016/S2589-7500(24)00224-3

**Published:** 2024-12-18

**Authors:** Joseph E Alderman, Joanne Palmer, Elinor Laws, Melissa D McCradden, Johan Ordish, Marzyeh Ghassemi, Stephen R Pfohl, Negar Rostamzadeh, Heather Cole-Lewis, Ben Glocker, Melanie Calvert, Tom J Pollard, Jaspret Gill, Jacqui Gath, Adewale Adebajo, Jude Beng, Cassandra H Leung, Stephanie Kuku, Lesley-Anne Farmer, Rubeta N Matin, Bilal A Mateen, Francis McKay, Katherine Heller, Alan Karthikesalingam, Darren Treanor, Maxine Mackintosh, Lauren Oakden-Rayner, Russell Pearson, Arjun K Manrai, Puja Myles, Judit Kumuthini, Zoher Kapacee, Neil J Sebire, Lama H Nazer, Jarrel Seah, Ashley Akbari, Lew Berman, Judy W Gichoya, Lorenzo Righetto, Diana Samuel, William Wasswa, Maria Charalambides, Anmol Arora, Sameer Pujari, Charlotte Summers, Elizabeth Sapey, Sharon Wilkinson, Vishal Thakker, Alastair Denniston, Xiaoxuan Liu

**Affiliations:** a University Hospitals Birmingham NHS Foundation Trust, Birmingham, UK; b National Institute for Health and Care Research (NIHR) Birmingham Biomedical Research Centre, Birmingham, UK; c Department of Bioethics, The Hospital for Sick Children, Toronto, ON, Canada; d Genetics and Genome Biology, SickKids Research Institute, Toronto, ON, Canada; e Birmingham Health Partners Centre for Regulatory Science and Innovation, Birmingham, UK; f NIHR Applied Research Collaboration West Midlands, Birmingham, UK; g NIHR Blood and Transplant Research Unit in Precision Transplant and Cellular Therapeutics, Birmingham, UK; h Centre for Patient Reported Outcomes Research, School of Health Sciences, College of Medical and Dental Sciences, Birmingham, UK; i University of Birmingham, Birmingham, UK; j Roche Diagnostics, Rotkreuz, Switzerland; k School of Clinical Medicine, Cambridge, UK; l Victor Phillip Dahdaleh Heart and Lung Research Institute, Cambridge, UK; m Hughes Hall, Cambridge, UK; n University of Cambridge, Cambridge, UK; o Department of Electrical Engineering and Computer Science, Massachusetts Institute of Technology, Cambridge, MA, USA; p Institute for Medical Engineering and Science, Massachusetts Institute of Technology, Cambridge, MA, USA; q Google Research, Mountain View, CA, USA; r Google Research, Montreal, Quebec, USA; s Google, Mountain View, CA, USA; t Department of Computing, Imperial College London, London, UK; u Independent Cancer Patients' Voice, London, UK; v Patient and Public Contributor, Sheffield, UK; w patient and public contributor, Munich, Germany; x NIHR Sheffield Biomedical Research Centre, Sheffield, UK; y School of Medicine and Population Health, University of Sheffield, Sheffield, UK; z Institute of Women's Health, University College London, London, UK; aa Institute of Health Informatics, University College London, London, UK; ab NIHR Great Ormond Street Hospital Biomedical Research Centre at UCL, University College London, London, UK; ac Tethyan Consulting, Canberra, Australia; ad Oxford University Hospitals NHS Foundation Trust, Oxford, UK; ae University of Oxford, Oxford, UK; af PATH, Seattle, WA, USA; ag Wellcome Trust, London, UK; ah Population Health Sciences Institute, Newcastle University, Newcastle, UK; ai Health Determinants Research Collaboration, Gateshead Council, Gateshead, UK; aj Google, London, UK; ak Leeds Teaching Hospitals NHS Trust, Leeds, UK; al University of Leeds, Leeds, UK; am Department of Clinical Pathology and Department of Clinical and Experimental Medicine, Linköping University, Linköping, Sweden; an Genomics England, London, UK; ao The Alan Turing Institute, London, UK; ap Australian Institute for Machine Learning, University of Adelaide, Adelaide, SA, Australia; aq Medicines and Healthcare products Regulatory Agency, London, UK; ar Department of Biomedical Informatics, Harvard Medical School, Harvard University, Cambridge, MA, USA; as African Biobanks and Longitudinal Epidemiologic Ecosystem, Ibadan, Nigeria; at Health Research Authority, London, UK; au King Hussein Cancer Centre, Amman, Jordan; av Harrison.ai, Sydney, NSW, Australia; aw Alfred Health, Melbourne, VIC, Australia; ax Monash University, Melbourne, VIC, Australia; ay Population Data Science, Swansea University Medical School, Faculty of Medicine, Health and Life Science, Swansea University, Wales, UK; az All of Us Research Program, National Institutes of Health, Office of the Director, Bethesda, MD, USA; ba Department of Radiology, Emory University School of Medicine, Atlanta, GA, USA; bb Nature Research, London, UK; bc The Lancet Digital Health, The Lancet, London, UK; bd Department of Biomedical Sciences and Engineering, Mbarara University of Science and Technology, Mbarara, Uganda; be Dermatopharmacology, Faculty of Medicine, Southampton, UK; bf University of Southampton, Southampton, UK; bg Department of Dermatology, University Hospitals Southampton NHS Foundation Trust, Southampton, UK; bh WHO, Geneva, Switzerland; bi PIONEER, HDR UK Health Data Hub in Acute Care, Birmingham, UK; bj NIHR Midlands Applied Research Collaboration, Acute Care Theme, West Midlands, UK; bk NIHR Midlands Patient Safety Collaboration, Birmingham, UK; bl National Institute for Health and Care Research, Southampton, UK; bm British Standards Institute, London, UK; bn NIHR Biomedical Research Centre, Moorfields Eye Hospital and University College London, London, UK

## Abstract

Without careful dissection of the ways in which biases can be encoded into artificial intelligence (AI) health technologies, there is a risk of perpetuating existing health inequalities at scale. One major source of bias is the data that underpins such technologies. The STANDING Together recommendations aim to encourage transparency regarding limitations of health datasets and proactive evaluation of their effect across population groups. Draft recommendation items were informed by a systematic review and stakeholder survey. The recommendations were developed using a Delphi approach, supplemented by a public consultation and international interview study. Overall, more than 350 representatives from 58 countries provided input into this initiative. 194 Delphi participants from 25 countries voted and provided comments on 32 candidate items across three electronic survey rounds and one in-person consensus meeting. The 29 STANDING Together consensus recommendations are presented here in two parts. Recommendations for Documentation of Health Datasets provide guidance for dataset curators to enable transparency around data composition and limitations. Recommendations for Use of Health Datasets aim to enable identification and mitigation of algorithmic biases that might exacerbate health inequalities. These recommendations are intended to prompt proactive inquiry rather than acting as a checklist. We hope to raise awareness that no dataset is free of limitations, so transparent communication of data limitations should be perceived as valuable, and absence of this information as a limitation. We hope that adoption of the STANDING Together recommendations by stakeholders across the AI health technology lifecycle will enable everyone in society to benefit from technologies which are safe and effective.

## Introduction

Artificial intelligence (AI) in health care has grown substantially over the past decade, with hundreds of AI products now available for use around the world.^1^, ^2^ There are many ways in which these technologies can transform health care, improving access to specialist-level diagnoses and treatments while reducing dependence on constrained health-system resources. However, the hope that AI will positively influence health-care delivery and outcomes needs to be balanced against the risks of harm associated with algorithmic biases.

A growing literature highlights the risk of AI health technologies exacerbating health inequity by both amplifying existing biases and generating new biases. For example, Obermeyer and colleagues^3^ demonstrated racial bias in an algorithm that was designed to guide health-care resource allocation. The root cause of this bias was the use of historical health-care costs as a proxy label during algorithm training to predict individuals' need for health care. The resulting algorithm systematically underestimated illness severity for Black patients, for whom resources were historically insufficiently allocated.^3^ Importantly, once discovered, measures could be taken to mitigate the effects of this algorithmic bias by reformulating the algorithm. Other examples of algorithmic bias include chest x-ray image classifiers trained on large public radiology datasets that underdiagnose pathology for underserved groups, and an acute kidney injury detection algorithm that underperformed for women because of sex imbalance in its training data.^4^, ^5^

Biases in AI health technologies can arise through many mechanisms, but one major source of bias is the data used to train, evaluate, and monitor them.^6^, ^7^ The ways in which data can contribute to bias in AI health technologies are complex and multifaceted. First, inadequate representation of particular groups in health datasets can result in reduced algorithmic accuracy, either because of insufficient sample size, or because the way groups are sampled does not give an accurate representation of that population in the real world.^5^, ^8^, ^9^ The majority of health datasets include data from only a small list of countries,^10^, ^11^, ^12^, ^13^, ^14^ and even within these countries, minoritised groups can be relatively under-represented.^15^ Additionally, data on demographic attributes are inadequately recorded, so the degree of representation of groups cannot be assessed.^10^, ^11^, ^12^, ^13^, ^14^ This problem is compounded by barriers to accessing health services, which mean that underserved groups are left out of so-called real-world datasets,^16^ and poorer-resourced settings might not have the infrastructure or funds to support data curation efforts, excluding whole populations and geographies. Second, data from underserved populations are more likely to be incomplete (recorded as unknown) or inaccurate.^17^, ^18^ Their presence might also be obscured if they are not explicitly labelled as a category—for example, because of a small sample size, through aggregation (use of other or mixed categories), or by not considering intersectionality (eg, intersections of age and gender). Individuals can sometimes be excluded or removed from a dataset because they do not map to a majority categorisation (eg, removal of patients who do not map to binary gender categories). Third, even if it were possible to achieve error-free measurement and recording of data, this error-free approach would not prevent encoding of societal and structural inequalities into datasets. These encodings are often diffuse and difficult to detect and measure.

Without careful and intentional dissection of the biases within datasets, it should be expected that health technologies built from them will inherit their limitations and assumptions, with the risk of amplifying and systematising bias wherever the technologies are used.^19^ AI health technologies are particularly sensitive to these considerations, and have the added challenge that it is not always possible to identify or constrain which data features drive algorithmic outputs. For example, algorithms have been shown to infer attributes such as race even if not explicitly trained to do so, which risks shortcut learning, in which the inferred attribute undesirably affects the algorithm's prediction.^20^, ^21^

Acknowledging these limitations can help everyone involved in creating, assessing, or using AI health technologies, to make better use of the datasets of today, and work towards improving the datasets of tomorrow. Recognising the presence of bias and studying datasets as artifacts can provide insight and knowledge about drivers of inequity, enabling targeted sociotechnical interventions to reduce embedded biases in datasets and their impact on AI health technologies.^22^, ^23^ To build towards more responsible use of health data, we firstly advocate for transparency—thoughtful, self-critical assessment of datasets, and clear reporting of limitations and biases, enabling a better understanding of whether a dataset serves as a sound basis from which to draw scientific conclusions or to build a technology. Secondly, we advocate for proactive inquiry of how biases can be introduced during AI development, through choices made by developers in data selection and model specification. We recommend transparent documentation of these decisions and testing of their effects on model performance across groups, particularly for groups that have worse health outcomes.

The Standards for Data Diversity, Inclusivity, and Generalisability (STANDING Together) programme is an international collaboration to build recommendations towards this vision. The programme started in December, 2021,^24^ and used standard consensus methodology to build recommendations that have broad relevance across differing contexts and jurisdictions. The process included a systematic review of existing approaches and a multistage modified electronic Delphi study to build consensus among a diverse range of stakeholders. These recommendations have a specific focus on datasets used for the development, evaluation, and monitoring of AI health technologies, and respond to increasing international awareness of algorithmic bias and the wider harms of under-representative, biased datasets.^25^, ^26^ The recommendations build on previous initiatives on the documentation of datasets within and beyond the medical domain,^27^, ^28^ and are intended to complement these and other efforts.^29^, ^30^ This Review describes the STANDING Together Delphi study, from development of the candidate items to the final recommendations and accompanying explanatory text. We believe that adoption of these recommendations will drive further progress towards AI health technologies that are not just safe on average, but safe for all.

## Methods

**Figure 1:**
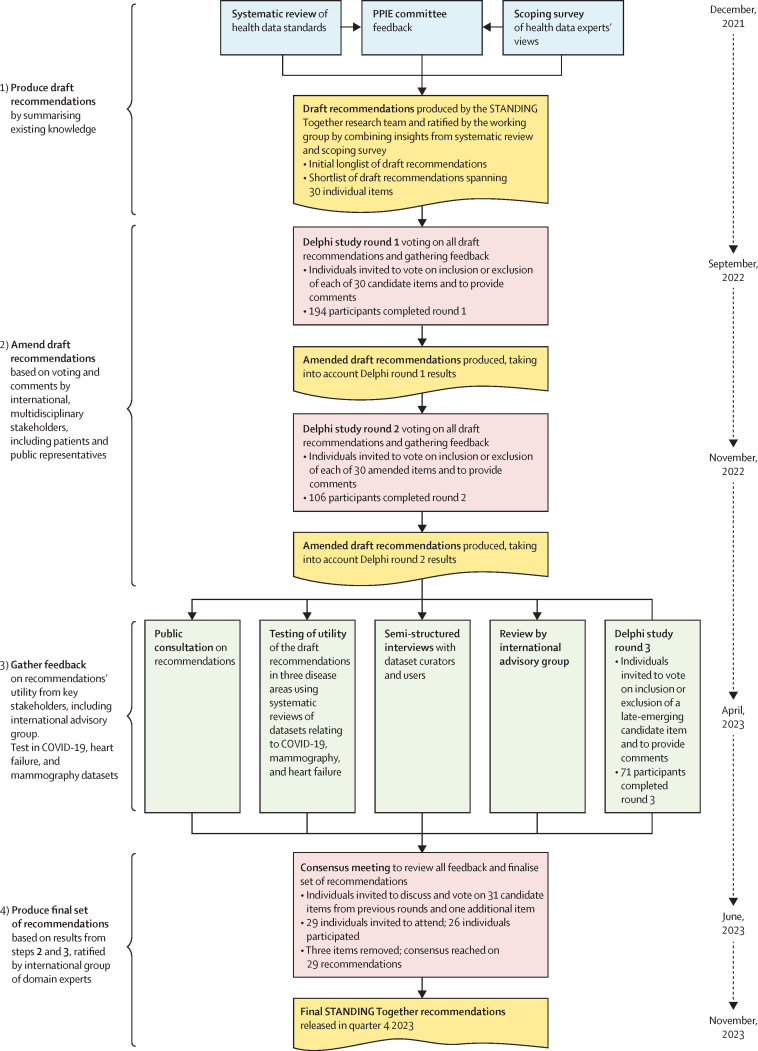
Overview of the design of the STANDING Together programme Draft recommendations were produced following a systematic review of existing approaches and a scoping survey of health data experts, in collaboration with feedback from our patient and public involvement and engagement (PPIE) committee. The draft recommendations were refined as part of an electronic Delphi study comprising three rounds of voting via an electronic survey and a consensus meeting to ratify the final items. Between the survey and the consensus meeting, feedback was sought through a public consultation and review by our international advisory group. The final recommendations were locked following the consensus meeting and are presented in this Review.

The STANDING Together recommendations were developed through a mixed-methods research programme from December, 2021, to November, 2023 (figure 1). A working group of international experts (AOA, AK, BM, CS, DT, ES, FMc, HC-L, JGa, JO, KH, LO-R, MG, MM, MCa, MMc, NR, NS, RM, RP, SK, and SP) working across different sectors—including health care, computer science, policy, regulation, and academia—and two patient co-investigators (AOA and JGi) was convened (by AD, EL, JEA, JGa, JP, and XL) to provide oversight throughout the project. To complement the expert input of the 23 members of the working group, a patient and public involvement and engagement (PPIE) committee of 12 members (CHL and JB) met quarterly and drew on their lived experience of health inequalities to guide the content and scope of the recommendations—in particular, to ensure that language used was inclusive, respectful, and accurate.

Ethical approval was granted by the University of Birmingham Science, Technology, Engineering, and Mathematics Ethical Review Committee (ERN_21-1831). Details of the study were published on the National Institute for Health and Care Research website before the project commenced.

### Development of draft recommendations

We undertook a systematic review of existing standards, frameworks, and best practices for health datasets, and a stakeholder survey exploring issues of bias, health equity, and best practices regarding AI medical devices. The search strategy, its selection criteria, and the results of this systematic review have been published separately.^31^ Themes extracted from the reviewed articles and survey responses informed an initial longlist of recommendations compiled by the STANDING Together research team and ratified by the working group and PPIE committee. Extracted recommendations were grouped according to where in the AI health technology lifecycle they applied, and similar concepts were merged to produce a shortlist of draft recommendations spanning 30 individual items.

### Building recommendations through a modified Delphi study

For the STANDING Together recommendations to be relevant across cultures and contexts, it was important to hear a wide range of views and to iteratively adapt sequential drafts of the recommendations in response to dissent and other forms of feedback. The Delphi method involves convening a group of experts and using both their responses to sequential questionnaires and their feedback to build a consensus on a topic.^32^ STANDING Together used a modified electronic Delphi study to build the final set of recommendations presented in this Review.^33^ In brief, this consensus process consisted of four rounds of voting: three online rounds held via electronic surveys, and one in-person round held via a consensus meeting.

#### Electronic survey rounds

Each Delphi survey was administered using Qualtrics (Qualtrics XM, Provo, UT, USA) and informed consent was obtained digitally from participants. The survey text was developed in collaboration with patient partners to ensure that the language was inclusive and interpretable. For accessibility, definitions for key terms and audio recordings were provided for all text in the survey. To lower technical barriers for participation, short videos explaining technical concepts were shown at the start of each survey. An example of the voting interface displayed to participants during rounds 1 and 2 of the survey is shown in the appendix (p 6). Each round of the survey was piloted by the working group (n=23) and PPIE committee (n=12). Participants were recruited through several mechanisms, including invitation of relevant experts as identified by the working group, publicly through the STANDING Together project website, through social media, and via an announcement paper^24^ in which readers could submit expressions of interest to participate in the Delphi study. Snowball recruitment was used to widen participants from diverse geographies. No incentives or rewards for participation were offered. Participants were asked to vote on inclusion or exclusion of each candidate item and to provide comments. Options on a five-point Likert scale were as follows: definitely exclude; probably exclude; unsure; probably include; and definitely include. Demographic information was also requested from participants. Although personalised invitations to participate were sent to individuals' email addresses, their responses were anonymised before analysis via deletion of any potentially identifying information. The questions from each Delphi survey round are provided in the appendix (pp 44–154).

A threshold for exclusion of 75% was set a priori, meaning that if 75% or more respondents voted definitely exclude or probably exclude for any item, the item would be removed from the draft recommendations and any subsequent voting rounds. Two full online rounds featuring every included candidate item were done to allow items to be amended before revoting on the basis of respondent feedback. A supplemental third round was added to address a single late-emerging candidate item, which arose from the public consultation. When items had been amended between rounds 1 and 2, participants were provided with both the original wording and the new wording. Round 1 was open from Sept 27 to Oct 17, 2022 (inclusive), round 2 was open from Nov 11 to Dec 2, 2022 (inclusive), and round 3 was open from April 26 to May 29, 2023 inclusive.

#### In-person consensus meeting

The fourth and final round of the Delphi study was a two-day consensus meeting hosted in June, 2023, at the University of Birmingham, Birmingham, UK. The meeting was hosted by XL and chaired by AD. Potential participants were identified from those who had previously expressed interest in participating in the Delphi study, and from the professional networks of members of the STANDING Together working group. These individuals included stakeholders with expertise across health care, data science, computer science, regulation, bioethics, social science, policy, and industry, in addition to patients and medical journal editors. In total, 29 individuals were invited and 26 individuals were able to attend. The final number of attendees was not prespecified; we opted for a pragmatic approach, ending recruitment when a balanced spread of expertise had been achieved among those able to attend. In addition, all five members of the STANDING Together research team and three additional volunteers attended to assist with the running of the event, but only the invited consensus-meeting participants were able to contribute to discussion and to vote on items. When required, arrangements were made to allow carers to accompany participants to the meeting, but they were not involved in the consensus process. 19 of 26 consensus-meeting participants were not involved in the design of the Delphi study; four attendees were representatives of the working group (BM, HC-L, JO, and MMc) and three attendees were representatives of the PPIE committee (AOA, CHL, and JB). All invited attendees were offered travel expenses and were provided with catering and accommodation for the duration of the meeting. In addition, PPIE committee representatives were compensated for their time. Consensus meeting participants were provided with information including the intended scope of the recommendations, the draft items, results from previous Delphi study rounds, explanations on how items changed between rounds, and a summary of comments from the Delphi survey and public consultation.

Before voting, consensus meeting participants discussed and agreed on the scope of the recommendations. Five statements detailing the purpose and context of the STANDING Together recommendations were coauthored live with the participants. During discussion of each recommendation item, live amendments were made and shared with all participants via a projector screen in response to comments and proposals from the consensus-meeting participants. When no further comments were offered, a vote was held on the item. All votes were held using PointSolutions software (Echo360; Youngstown, OH, USA). Participants were able to vote to include, exclude, or abstain for each item. Items were included if 70% or more votes were to include. Further, a quorum of 90% was applied, so that if 10% or more participants voted abstain, the vote would be nullified and the item rediscussed. When the quorum was met, items receiving fewer than 70% of include votes were removed from the recommendations. Panellists were invited to explain their responses if they had chosen to abstain and the item was entered into rediscussion. As well as voting on items' exclusion, participants were also given the opportunity to propose new items. Detailed notes were kept throughout discussions so that any unresolved comments could be actioned after the meeting, while correcting language and grammar and determining the final order in which the recommendation items would be presented.

A final draft of the recommendation items was disseminated for review by consensus-meeting participants and the working group, with a glossary of key terms (panel 1) and explanatory text for each item authored by the research team, to provide context and elaboration. The individual items were locked following this final review, forming the STANDING Together recommendations published here.

Panel 1 **Glossary of key terms**

**Attribute**

A measured characteristic of an individual or group of individuals that can be biological, socially constructed, or a combination of both. Groups of individuals can be defined according to attributes (eg, blood test results, diagnoses, health outcomes, employment status, language, national identity, age, sex, gender, race, and ethnicity). Relevant attributes, for the purposes of these recommendations, encompass a wide array of characteristics that have potential associations with health outcomes, including but not limited to demographic, social, and economic factors.

**Bias (of datasets or algorithms)**

For the purpose of the STANDING Together recommendations, bias is defined as a systematic deviation from objectivity or neutrality, which can occur at any stage throughout the artificial intelligence (AI) development lifecycle. In the health-care context, bias can reflect differences between groups that are driven by factors that do not have clinical relevance. In the context of datasets, bias can arise because of differences in the rate or characteristics of errors or missingness between groups, or because of underlying distortions in the environment from which data are recorded (including structural and systematic discrimination and oppression). Biases in the outputs of algorithms (algorithmic bias) can unfairly disadvantage certain groups over others, and might reflect biases in datasets or result from choices made during development.

**Contextualised groups of interest**

A group of individuals with shared relevant attributes that have known or suspected associations with disparate health outcomes related to the intended use of an AI health technology. Groups can be defined by multiple attributes (eg, age in combination with gender), particularly when these factors might interact and compound disparate health outcomes (see intersectionality). Disparate health outcomes can be driven by a variety of factors, including structural and social determinants of health,^34^ biological factors, or by the AI health technology under development (including datasets used and the effect of implementation on the health-care pathway; a non-exhaustive list of potentially relevant attributes is provided in the appendix [p 2]). The relative contribution of each factor can evolve over time.

**Data custodian**

Individuals or organisations legally (or otherwise) responsible for the dataset.

**Data origin**

**Figure 2:**
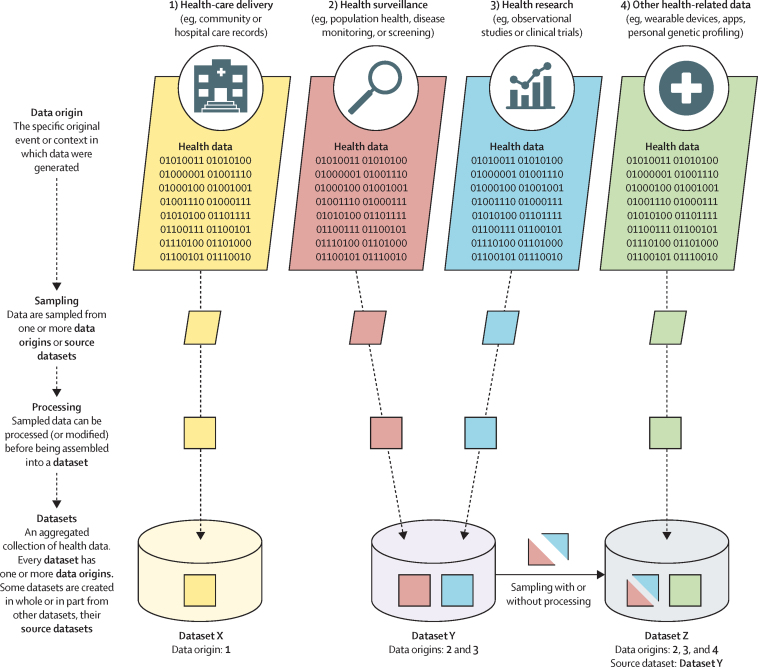
Datasets, data origins, and source datasets All health datasets have one or more data origins, the specific original event or context in which primary health data were generated. Data origins can include several categories: direct delivery of health care, such as records from primary or secondary care services, emergency services, or care facilities (origin 1); health surveillance, such as population or public health records, national disease monitoring, or screening for diseases in asymptomatic populations (origin 2); health research, such as observational studies or clinical trials of health interventions (origin 3); and other health-related data, such as data from wearable devices, health and wellbeing apps, and direct-to-consumer biomedical testing data including genetic profiling (origin 4). Some health datasets are created using only data obtained directly from one or more data origins. Other datasets are created in whole or in part by sampling data from source datasets. Datasets can also combine data obtained directly from data origins and data from source datasets. For example, dataset X is composed only of data from data origin 1 and has no source datasets. By contrast, datasets Y and Z are each composed of data from more than one data origin. Dataset Y has two data origins (2 and 3) but has no source datasets. Dataset Z samples data directly from data origin 4, but also includes data from dataset Y, its source dataset. Accordingly, dataset Z contains data from data origins 2, 3, and 4.

The original event or context in which data were generated (figure 2). This should usually include details of the specific setting and geographical location in which data were created. For instance, data generated through provision of health care in a secondary care hospital in Geneva, Switzerland; data generated on participation in a clinical trial done across 20 hospitals in Japan; or data generated through interaction with a consumer-facing app freely available via the internet. When the data origin is a provider of health-care services, details of the health-care system in which it operates should also be included (eg, details of how the system is funded and accessibility of health-care services).

**Data shifts**

Changes in the statistical nature (or distribution) of the data (or any subset of the data) within a dataset occurring as a reflection of changes in the system from which data are obtained. Data shifts commonly refer to changes over time (eg, practice updates leading to changes in the way health care is delivered or changes in the prevalence of a disease over time), but they can also refer to changes in data distribution in other contexts (eg, differences in the composition of groups contained within a dataset, differences in the way data were collected between groups, or differences in the way data are generated across health-care settings and contexts). Substantial data shifts can result in a mismatch between the data used to train an algorithm and the context in which it is intended to be used, affecting its performance and contributing to bias.

**Data user**

Individuals or organisations who use a dataset in the lifecycle of an AI health technology.

**Dataset**

An aggregated collection of data. In the health-care context, data contained in a dataset might be tabular (containing columns and rows of text or numerical data, such as the blood test results of patients), medical images or videos (eg, a collection of knee x-ray images, or a video captured during coronary angiography indicating arterial blood flow), audio (eg, a collection of recordings of heart sounds), or other more complex designs, including combinations of tabular data and other data types.

**Dataset curator**

Individuals or organisations involved in any of the stages of creation of a dataset. Data curators can be involved in various decisions relating to determining the purpose of the dataset, making the choice of what to measure and how, obtaining the data itself (including designing and applying any sampling strategies), structuring the dataset, preprocessing the data or data modification, and making data available for use.

**Data instance**

A term used to describe a single unit of data within a specific context. For example, a data instance might refer to a single observation among a set of observations contained within a dataset. In the health-care context individual patients can be represented as data instances, although where individual patients receive health care multiple times, each episode of care might be considered as a separate instance.

**Error**

A discrepancy between the expected and observed values in a system. In the context of a dataset, error can manifest as inconsistency between the dataset and the environment from which it is derived, such that the recorded data do not accurately reflect reality. In the context of an algorithm, error can manifest as a discrepancy between a prediction and the truth. Errors can occur at any stage during dataset creation, including during measurement, recording, processing, and transmission of data. Imprecision caused by constraining complex biological or social phenomena into categories can also lead to error.

**Fairness methods**

In the context of AI, fairness methods refer to statistical approaches used to compare a performance metric or other statistical property across groups for whom an algorithm is used. Furthermore, a variety of algorithmic techniques exists to develop models that satisfy a fairness constraint, such as methods applied to model development with the goal of reducing the performance differences between subgroups.

**Features**

Features are measurable properties of a dataset that are used as inputs for machine learning algorithms. Often the term is used interchangeably with the term variable. In the context of tabular data, features can be conceptualised as the observations or measurements within the data, representing set values (eg, height, weight, and blood sugar concentration) for individual data instances. In the context of images and other non-tabular data, features can represent a part of the data with particular relevance (eg, the edge of an anatomical structure). In some cases, features and attributes can represent the same phenomena.

**Harm**

The STANDING Together recommendations use a broad, sociotechnical definition of harm, recognising that algorithmic harm has several potential manifestations. It has been broadly defined as adverse lived experiences resulting from a system's deployment and operation in the world.^35^ In a regulatory context, harm is defined as injury or damage to the health of people, or damage to property or to the environment.^36^, ^37^ Individuals can be personally harmed by an AI health technology (eg, misdiagnosis leading to the wrong treatment for the patient), or harm can be indirect or at the group level (eg, an AI health technology that worsens health inequity by contributing to adverse health outcomes for certain groups when compared to others,^5^ or that contributes to testimonial injustice, in which the testimony of an individual is perceived as less reliable than an algorithm's output).^38^

**Imputation**

A statistical process used to generate data as a replacement for missing data in a dataset.

**Intended use (also known as intended purpose)**

The purpose or purposes for which the supplier of an AI health technology specifies that they intend the technology to be used. In the context of AI health technologies that are medical devices, the intended use would generally be specified by the manufacturer or person or organisation legally responsible.

The International Medical Devices Regulators Forum defines intended use as the objective intent regarding the use of a product, process, or service as reflected in the specifications, instructions, and information provided by the manufacturer.^39^ National medical device regulators have adopted similar but slightly different definitions and terminologies (eg, indications for use) across jurisdictions, including the US Food and Drug Administration,^40^ the UK Medicines and Healthcare products Regulatory Agency,^41^ Health Canada,^42^ and the Therapeutics Goods Administration, Australia.^43^

**Intended use population**

The population for whom an AI health technology can be used, as defined by the supplier or manufacturer.

**Intersectionality**

Understanding the lived experience of an individual and its impact on their health outcomes requires considering several aspects of their identity and life circumstances rather than focusing on a single attribute in isolation. Intersectionality refers to how individuals are subject to several, intersecting forms of privilege or disadvantage. The combined effect of these intersecting factors on health outcomes cannot be predictable on the basis of the effect of each in isolation. Some factors are products of the sociopolitical environment, some are the product of oppression and discrimination, and others are the subtle influences of socialisation.^44^, ^45^

**Labels**

Labels (or data labels) refer to annotations or information assigned to each data instance, to provide meaning to the raw data. Not all datasets have labels. Data labels are commonly used in supervised learning tasks, in which associations are learned between features within the data and their corresponding labels. In the health-care context, these labels are often clinical outcomes. Labels can be generated by human experts, or by an algorithm. In some cases, labels are objective measurements, whereas in other cases they can be subjective assessments.

**Lifecycle of AI health technology**

The lifecycle of an AI health technology describes all steps involved in the creation and use of the technology, spanning the initial idea generation through to the marketisation, deployment, decommissioning, and disposal of the final product. Safety and quality monitoring after deployment is part of the lifecycle.

**Metadata**

Data that defines and describes other data (International Organization for Standardization and International Electrotechnical Commission 11179).^46^ In a health dataset, metadata can relate to clinical context or technical details (eg, the indication for which a chest x-ray was requested by a clinician, and details of the specific x-ray machine used to acquire the image).

**Missingness**

The patterns in which data are missing within a dataset. Recognising and understanding these patterns and their implications is important when assessing whether a dataset is appropriate for a particular use case. Failing to account for missingness when developing AI health technologies can be a cause of algorithmic bias.

**Permitted uses**

Any activities for which the dataset can explicitly be used.

**Persistent identifier**

A permanent and unique reference by which a dataset can be identified. Digital assets (including datasets) are often assigned a digital persistent identifier (eg, a digital object identifier).^47^

**Plain language**

Communication in which wording, structure, and design are so clear that intended readers can find what they need, understand what they find, and use that information (International Organization for Standardization 24495-1).^48^ This can include the use of simple words and grammar and avoidance of jargon and technical language. Plain language makes it easier for non-expert readers to understand what is written. There are electronic tools available to assess the complexity of written language, including readability scores built into word processors, and dedicated online tools.

**Purpose (of a dataset)**

The reason why a dataset was created and made available. For instance, to develop a specific algorithm, for a grand challenge or datathon, or as a generic resource for researchers.

**Source datasets**

Any upstream datasets used in whole or in part in the creation of a new dataset (figure 2). Whereas all datasets will have one or more data origins, not all datasets will have source datasets.

### Consultation on draft recommendations between Delphi study rounds

Following the first two Delphi study survey rounds, a public online consultation was held using Qualtrics (Qualtrics XM; Provo, UT, USA) to further gather input on the draft recommendations. To facilitate this public engagement, a green paper (a report detailing the draft recommendations and how they had been developed) was produced and disseminated to our project contact list, via social media, and on the project website. The draft recommendations were also presented at the 2022 US Symposium for Artificial Intelligence for Learning Health Systems (SAIL). Both the green paper and the SAIL presentation requested online feedback, which was open for 4 weeks.

To seek additional international input, an international advisory group composed of 34 individuals from 14 countries was convened. Members of this group were sought from low-income and middle-income countries to increase international diversity and to ensure the draft recommendations could be globally applicable. The STANDING Together Research team met with members of the international advisory group quarterly three times between February and October, 2023 and held more frequent discussions online to gather an international perspective on the content and wording of recommendation items, particularly relating to the interpretability and applicability of key concepts, such as demographic attributes and concepts of race and ethnicity in different countries and cultures.

Concurrently we conducted an interview study with semi-structured interviews of 28 experts in health dataset creation and use. The purposes of this interview study were to inform the scope of our draft recommendations (the contexts in which they should apply), and to identify challenges to adopting health dataset standards, both generally and with specific reference to our draft recommendations. Detailed methods and results relating to this interview study will be reported separately.

### Statistics and reproducibility

After each Delphi survey round, a set of plots was created showing each item's results, categorised by stakeholder group. Following the third voting round, summary statistics (median, IQR, and the percentage of respondents voting to probably include or definitely include) and plots highlighting the performance of each item across each voting round were produced using R version 4.1.1 and the packages tidyverse,^49^ magrittr,^50^ readxl,^51^ RColorBrewer,^52^ ggthemes,^53^ and rworldmap.^54^

### Role of the funding source

The funders of the study had no role in study design, data collection, data analysis, data interpretation, or writing of the report.

## Development of recommendations

### Scope of the STANDING Together recommendations

In defining the scope of STANDING Together, two distinct perspectives emerged: those of the dataset curators, who build datasets; and those of the data users, who analyse and use datasets for a particular purpose (panel 2). These groups, when they are not overlapping, have different needs and goals, and different levels of influence in shaping and constructing the datasets. It became apparent that the ability to conceptualise data bias is contingent on a clearly defined context or use case, and the approach to mitigating its impact depends on whether an individual or team is acting as a data curator or user. These contrasting perspectives led to the creation of two complementary parts of the recommendations: first, Recommendations for Dataset Documentation, aimed at dataset curators; and second, Recommendations for Dataset Use, aimed at data users. There are 29 individual recommendations across both sections.

Panel 2 **Intended scope of the STANDING Together recommendations**

The following statement on the intended scope of the STANDING Together recommendations, which include Recommendations for Documentation of Health Datasets and Recommendations for Use of Health Datasets, was drafted as part of a qualitative research study for the development of these recommendations, and refined by attendees at the consensus meeting. This qualitative study will be published separately.

The STANDING Together recommendations constitute best practice in the documentation and use of health datasets for artificial intelligence (AI) health technologies. Individual organisations (such as funders or regulators) might choose to mandate them, but that is entirely at their discretion. The recommendations have value at every stage in the AI health technology lifecycle. They might have particular relevance for datasets used in later stages of development of AI health technologies and regulatory approval. The recommendations are sufficiently principle based to be relevant to any and every jurisdiction. They should be interpreted and contextualised to address any particular issues for each setting in which they are applied. They contain sufficient detail to allow users to see easily how to operationalise these principles, while remaining sufficiently flexible for the diversity of populations and settings in which they might be used. It might not always be possible to meet all the requirements of the STANDING Together recommendations. To ensure transparency, if all requirements are not met, this should be documented and explained, and highlighted as a source of uncertainty with the potential to cause or contribute to harm.

### Consensus process

#### Delphi study

The modified electronic Delphi study consisted of three rounds of online voting through surveys administered via the internet, and a final round consisting of an in-person consensus meeting (figure 1). 194 participants completed the first round of the Delphi study, 106 completed the second round, and 71 completed the third round (see appendix pp 3–5 for information about Delphi survey participants). Averaged across all three online Delphi study survey rounds, 30·5% of participants were computer or data scientists, 20·5% were health-care professionals, 15·6% were members of the public, 7·5% were experts in policy, regulation, or law, and 25·9% worked in other roles (appendix p 7). Across all three Delphi survey rounds, no items met the threshold for removal. 26 people participated in the consensus meeting, where each item was discussed, amended if necessary, and voted on. Three items were removed and one item was added. A record of the performance of each individual item and how it was modified across the three Delphi survey rounds and during the consensus meeting is provided in the appendix (pp 9–43).

#### Participation

**Figure 3:**
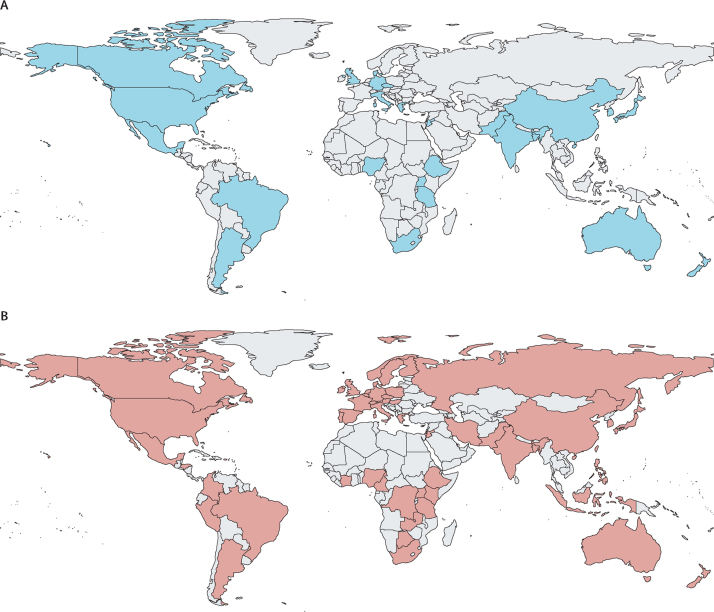
Reach of the STANDING Together initiative (A) Geographical locations of the participants in the Delphi study (25 countries). (B) Overall reach of the STANDING Together initiative (a total of 58 countries), including participants in the Delphi study, members of the patient and public involvement and engagement committee and international advisory group, members of the working group and attendees at the consensus meeting, participants in the interview study, and individuals who reviewed the draft standards or the green paper via the project website. Some countries are represented more than once. The reach of the STANDING Together project disaggregated by type of involvement is presented in the appendix (p 8).

Including those who accessed the draft recommendations or the green paper via the project website during the public consultation, the STANDING Together project has so far reached 58 countries (figure 3). Representatives from 25 countries contributed to the Delphi study, the members of the international advisory group and PPIE committee represented 16 countries, the interview study involved representatives from 14 countries, and consensus meeting attendees and working group members represented nine countries (appendix p 8). Participants in the creation of these recommendations were individuals working across a range of sectors—including health-care delivery, regulation, policy, academic research, computer science, AI development, and research funding, among others. We also included patient representatives and members of the public both as coinvestigators and as participants.

## Recommendations for the Documentation of Health Datasets

Dataset documentation gives data meaning by describing its provenance and nature. Effective documentation enables potential users of a dataset to decide whether it is suitable for their purpose, on the basis of its content, context, and limitations. Documentation should accompany all datasets. For new datasets, new documentation should be created. For datasets created from pre-existing source datasets, any existing documentation can simply be referenced, so long as the information remains accurate and representative for the new dataset (figure 2). If any information is unavailable, this should be transparently reported.

Here we present 18 recommendations for dataset documentation, aimed at dataset curators, including the following: dataset summary (item 1.1a), dataset identity and access (1.1b), reasons behind dataset creation (1.1c), data origin (1.1d), data sampling and aggregation from multiple sources (1.1e), data shifts over time (1.1f), groups within the dataset and attributes of individuals (items 1.2a–1.2c), sources of bias (1.3a–1.3f), ethics and governance (1.4a), patient and public participation (1.4b), and bias and impact assessments (1.4c).

### Recommendation 1.1a: dataset summary

Dataset documentation should include a summary of the dataset written in plain language. This summary should state the data origin and its purpose, and give a short description of the content to help data users assess whether the dataset meets their needs (appendix p 10).

#### Explanation

The dataset summary should help data users to determine whether the dataset meets their needs. An effective dataset summary should be similar to the abstract of a research paper and understandable to a wide audience—concise, structured, and written in plain language, with explanations for any necessary technical terms. To be useful, this summary should provide context regarding the source and purpose of the dataset and an overview of the content. The summary should include a description of the data origin, including the time period over which data were collected. This information should include the number of individuals represented in the dataset and the way in which individuals can be distinguished from one another, particularly if there were several records for certain individuals. The purpose of the dataset should include a summary of why the current dataset is being made available (eg, to develop a specific algorithm, for a grand challenge or datathon, or as a generic resource for researchers).

### Recommendation 1.1b: dataset identity and access

Dataset documentation should: (1) State the dataset's identity, including a persistent identifier and information regarding date(s) of release. (2) Provide information on how the data can be accessed, including permitted use, licensing arrangements and details of the data custodian(s). (3) Describe adherence to principles for data use and access (appendix p 11).

#### Explanation

It is essential that datasets can be uniquely and persistently identified to enable all outputs based on the dataset to be traced. Dataset documentation should relate to a specific dataset, and relevant identifying information about the dataset should therefore be provided (this information could include version number and other metadata). Datasets might require specific permissions to be granted before access, and they might have restrictions regarding how they can be used and by whom. Permitted use describes any activities for which the dataset can explicitly be used, and licensing arrangements should describe any limitations or restrictions. Adherence or compliance to recognised principles for data use and access (such as the FAIR principles) should be considered where possible, but this is not a specific requirement; the FAIR principles aim to make data Findable, Accessible, Interoperable, and Reusable.^55^

### Recommendation 1.1c: reasons behind dataset creation and its purpose(s)

Dataset documentation should include the reasons why this dataset was created, including any intended benefit(s), any purposes for which dataset use should be avoided, who created the dataset (including any competing interests), and who funded it (appendix p 12).

#### Explanation

Some datasets are created for a specific purpose, whereas others will be created for several potential applications. For example, a dataset of chest x-ray images might be created with the specific intention of developing an algorithm to detect pneumonia. On the other hand, a dataset of chest x-ray images might be created as part of an imaging biobank, for researchers to use for many purposes. The reasons behind dataset creation might have influenced decisions during the creation process, and this information might be relevant for the data user. There might also be situations in which a dataset should not be used—for example, because of legal and ethical constraints on what the dataset can be used for, or in recognition of certain limitations of the data. Dataset documentation should include a statement that explains how it was funded and explicitly declares real or perceived competing interests. If there are no competing interests to declare, there should be a statement to this effect.

### Recommendation 1.1d: data origin

Dataset documentation should describe the data origin, why it was selected, and what individuals were told would happen to their data (appendix pp 13–14).

#### Explanation

Describing the data origin (eg, whether the origin is patient records to provide clinical care, a clinical trial, a biobank, or equivalent) can provide useful context for data users. When several data origins were used, information should be provided for each data origin. Data collected as part of a clinical trial or other research study might be systematically different from that collected during routine clinical care, even if it relates to the same individuals and clinical situation (eg, eligibility criteria could mean that the composition of groups present in a clinical trial dataset is different from that of a dataset of routine care, or the requirement to complete a case report form might result in more complete ethnicity data than in routine care data). Reasons for the choice of data origin should also be stated, because these reasons could reveal strengths and limitations. Dataset curators should describe what information individuals were given about how their data would be managed and used (eg, for administrative purposes in health-care delivery or for research purposes as part of a study), and whether consent was granted by individuals. There is an ethical obligation (and in many jurisdictions also a legal requirement) to inform individuals about how their data will be used, and seeking informed consent might be necessary. If individuals were not informed or asked for consent, or if no information about this is available, this absence of consent should be described.

### Recommendation 1.1e: data sampling and aggregation from multiple sources

Dataset documentation should describe how data were sampled from the data origin and any source dataset(s), including an explanation of sampling strategies, their rationale, and potential impact on the composition of the dataset. If the dataset has been compiled from multiple data origins or source datasets, dataset documentation should describe how each was selected, and how decisions were made during data aggregation, particularly in the case of grouping populations and modification of demographic coding (appendix pp 13–14).

#### Explanation

In the context of dataset creation, sampling is the process of selecting which data should be included from the data origin and any source datasets. The specific way in which data are sampled could influence the composition of the final dataset, which could contribute to bias. For example, sampling data from large, urban hospitals might exclude groups of individuals residing in more remote settings. As well as stating the sampling strategy used, the reasons for choosing this strategy and its effect on the dataset should be described, so that data users can consider the risk of bias and possible consequences. When combining data from several data origins or source datasets, the ways that populations are grouped and how their associated data are recorded could differ. For example, race and ethnicity categories are not consistently defined across countries, and in some settings, different concepts (eg, tribe or caste) might be more relevant. If any data are modified to enable aggregation from several data sources (eg, combining ethnic groups into broader categories), the reasons for doing so and methods used should be stated because this might affect the use and interpretation of the data, and could lead to aggregation bias (see also item 1.3a). It is good practice to provide a plain language summary of any code used to enable data aggregation, detailing its purpose and mechanism. Where possible, the code itself should also be shared.

### Recommendation 1.1f: data shifts over time

For longitudinal datasets or datasets with multiple versions, dataset documentation should describe any known or expected changes over time relating to the population, medical practice, or how data were collected (including devices, sensors, and software used), which may contribute to data shifts over time (appendix p 15).

#### Explanation

Data shifts over time represent changes in the statistical nature or distribution of all or some of the data included in a dataset. Substantial data shifts over time can result in a mismatch between the data used to train an algorithm and the context in which it is intended to be used, affecting its performance and contributing to bias. Data shifts can occur as a result of population changes (eg, changes in the age distribution of a country as a result of lengthening life expectancy), changes in the way health care is delivered (eg, introduction of a new laboratory blood test or a new treatment for a disease), or changes in the way data are measured, recorded, and collected (eg, replacing paper medical records with electronic health records or the introduction of wearable monitors to measure patients' vital signs), and many other factors. Data shifts over time can be gradual (eg, a slow rise in life expectancy over time as health care improves) or sudden (eg, a rapid decrease in mortality from an infectious disease after the roll-out of effective vaccinations). These data shifts over time might be detectable by analysing the pattern of data recorded in a dataset, or informed by knowledge of the clinical context. Knowing about data shifts over time allows dataset curators to provide additional context that might not be available to data users unless explicitly stated. Certain data shifts over time might be known to be present, some might be expected but not proven, whereas others might not be detectable. All known and expected data shifts over time should be described, allowing data users to consider any potential effects when they use the data for development.

### Recommendation 1.2a: composition of groups within dataset

Dataset documentation should: (1) Include a summary of the groups present in the dataset. The choice of which groups to describe, and the means of categorisation should be explained. (2) Highlight any known missing groups within the dataset and any reason(s) for their missingness (appendix p 16).

#### Explanation

Individuals who share attributes can be grouped within datasets (eg, by race, ethnicity, sex, gender, or age). The ways in which groups are defined within a dataset should be described, given that grouping concepts might not be universal and can introduce assumptions about individuals and contribute to bias. Data users will use information about the composition of a dataset to assess its suitability for their purpose. As such, knowledge of the groups the dataset includes (ie, who is represented in the dataset) is crucial information in making this assessment. In some cases, groups will be absent from a dataset because its clinical context or purpose is not relevant to them (eg, adults would not be present in a dataset of paediatric chest x-ray images). In other cases, groups might be absent for structural reasons—for example, if they are not represented in the data origin or the sampling process leads to their exclusion (eg, individuals living with mobility impairment might be less able to attend an appointment to test eligibility for a clinical trial, and so might be under-represented in a subsequent dataset of trial data). Additionally, labels might not precisely capture any groups that exist in the dataset. When certain groups that would be expected to be present are known to be absent from the dataset, this should be explained (see also items 1.3c and 1.3e).

### Recommendation 1.2b: recording of individuals' attributes

Dataset documentation should: (1) Describe how and why individuals' attributes are provided in the dataset, and whether this information is available at the individual or aggregate level. (2) Explain whether attributes have been coded, condensed, derived, or modified, stating how and why this was done. (3) Highlight the proportion of attributes recorded as “unknown” or “other” and “prefer not to say”, and if possible explain the reasons why (appendix p 17).

#### Explanation

Wherever possible, attributes that are assigned by humans should be self-reported by individuals represented in a dataset.^56^ If attributes have been reported by others (eg, a health-care professional or a scientist conducting a study), are computer generated (eg, via imputation or linkage from other datasets), or are modified in any way, the method and rationale for doing so should be explained because this can be a source of bias.^57^ Coding or condensing attributes in a way that causes several attributes to be combined or simplified is a form of modification and should be transparently reported. If possible, dataset curators should describe how the original attributes were ascertained and provide the original attributes themselves, including any missing or erroneous values and those generated or modified. If a computational or automated approach has been used for generation or modification of attributes, transparency can be enhanced by providing a plain language summary of its purpose and mechanism, and where possible the code itself should also be shared. Attributes documented as “unknown”, “other”, and “prefer not to say” (or equivalent) should be reported, and the reasons why they are reported this way should be explained to enable data users to understand the limitations and uncertainties associated with the data.

### Recommendation 1.2c: groups at risk of disparate health outcomes

Dataset documentation should: (1) Include data (when available) on relevant attributes relating to individuals included within the dataset. If including these data may place individuals at risk of identification or endanger them, these data should instead be provided at aggregate level. If data on relevant attributes are missing, reasons for this should be stated. (2) Highlight the presence of groups who are at risk of disparate health outcomes caused by structural or societal factors in this dataset, with consideration of both risk factors that are universal, and those that are specific to the site of data collection (appendix pp 18–19).

#### Explanation

Relevant attributes are characteristics of individuals or groups that have potential associations with health outcomes, including but not limited to demographic, social, political, and economic factors. These associations can reflect interaction with a biological process or the influence of sociocultural factors, including structural and systemic marginalisation, discrimination, and oppression. In some cases, relevant attributes might have a positive effect on health outcomes, benefiting some groups over others. Which attributes are relevant attributes depends on the context and purpose of the dataset and might not be consistent across countries and cultures (eg, caste groupings might be a social determinant of health and hence a relevant attribute in some countries, but not in others). Depending on the context, relevant attributes might include (but are not limited to) sex, gender, race, ethnicity, age, socioeconomic status, sexual orientation, disability, pregnancy, relationship or marriage status, religion or belief, nationality, ancestry, occupation, language or languages spoken, caste, creed, or tribe.

If data on relevant attributes are not provided, data users cannot interpret or account for disparate health outcomes that might be associated with these attributes. Additionally, data users cannot identify under-representation within the dataset or audit for potential disparate performance of a technology in relation to these attributes (ie, data disaggregation).^56^ It should be noted that some attributes might risk identification of individuals, particularly in the case of rare attributes and when several datasets relating to the same individuals could be linked. In some situations, identification of individuals might lead to direct harm (eg, data about an individual's sexual orientation might cause them to face imprisonment or capital punishment in some countries). The principle of do no harm should apply when data relating to personal identity are made available (ie, self-identification).^56^ The risk that individuals might be identified by the inclusion of any attributes in the dataset should be assessed (eg, as part of a data protection impact assessment; see item 1.4c). This risk assessment should be documented, including any mitigation steps taken and their justification. Mitigation steps might include providing data at aggregate rather than individual level (eg, stating the proportion of individuals in each category rather than providing an identity for each individual).

Examples of universal risk factors include extremes of age, people living with severe disabilities, and displaced people and refugees. Examples of risk factors specific to the site of data collection include marginalised religious, caste, or cultural groups.

### Recommendation 1.3a: limitations of the dataset

Dataset documentation should identify known or expected sources of bias, error, or other factors that affect the dataset as a whole, which may impact its generalisability or applicability (appendix p 20).

#### Explanation

All datasets have limitations. Although some limitations might be detectable to data users appraising the dataset, others will be knowable only to dataset curators. These limitations comprise data errors, missing data, and other deficiencies that could affect all groups equally (ie, no individual is at any greater risk of being affected than any other) or might affect one group more than another (ie, a systematic bias that places certain individuals at higher risk than others). Both types of limitation should be documented, including both known and expected limitations. When known or expected biases, errors, or other factors are identified, they should be reported and discussed in dataset documentation, including any possible effect they might have on dataset use. When there is uncertainty about the presence or importance of a dataset limitation, the presumption should be towards disclosure and discussion.^25^ Where possible, dataset curators should reflect on the effect their own previous assumptions and preconceptions might have had on biases in the dataset (eg, in the choice to focus on certain clinical problems in the dataset, in the strategy for sampling, coding and processing of data, in the way individuals were grouped and the categories used, and in the way missing data were managed).

### Recommendation 1.3b: modifications made to the data

Dataset documentation should describe whether any data items were modified from the original source, or if any of the data are synthetic, providing the rationale for doing so and any methods used (appendix p 21).

#### Explanation

Where possible, data contained within datasets should be unmodified, because the process of modification can contribute to bias. When modification has been undertaken (eg, combining ethnic groups into broader categories), the rationale for doing so and methods used should be clearly explained to enable the data user to mitigate for any potential biases, and it is good practice to provide both modified and unmodified data. Reasons for data modification can include (but are not limited to) anonymisation or de-identification, remapping or recoding to existing data standards (eg, the Observational Medical Outcomes Partnership^58^ or Fast Healthcare Interoperability Resources), compensation for errors in the source data, and preprocessing or transformation as an initial step to enable data use. Synthetic data refers to data that are generated artificially by a computer system, often intended to resemble real-world data. Reasons for generating derived, imputed, or synthetic data might be well intentioned—for example, to address missing data values (eg, imputing missing test results for individuals) or to add additional data to a dataset (eg, attempting to mitigate under-representation of certain groups by adding synthetically generated data to a dataset). However, these methods might themselves introduce bias. It is good practice to provide a plain language summary of any code used for modification or imputation of data that details its purpose and mechanism. Where possible, the code itself should also be shared.

### Recommendation 1.3c: missing data

Dataset documentation should describe the proportion, nature, and causes of missing data (if known), particularly if there are systematic differences across groups within the dataset. Documentation should also describe if and how missing data have been handled (eg, imputation; appendix p 22).

#### Explanation

Missing data refers to the absence of specific information in a dataset where it would be expected to be present. Providing details of the proportion, nature, and causes of missing data can enable data users to compensate for this. Dataset documentation should highlight and explain the causes of differences in the pattern of missing data across groups (eg, individuals experiencing poverty might be less able to afford to travel to hospital appointments, meaning that a dataset of routine clinical care might have more missing data for these individuals). When attempts have been made to handle missing data (including imputation to replace missing values), the rationale for doing so and methods used should be described in the dataset documentation. Both the unmodified data and any data that have been modified, and a plain language summary detailing the purpose and methods behind any code used, should be provided. Where possible, the code itself should also be shared.

### Recommendation 1.3d: known or potential bias caused or exacerbated by data acquisition and processing

Dataset documentation should: (1) Describe how bias may be introduced by the acquisition and processing of data within the dataset. (2) Highlight any known or potential differences in data acquired across different groups, or differences in the uncertainty of measurements between groups. (3) Describe any attempts to mitigate these biases (appendix p 23).

#### Explanation

The acquisition and processing of data generated by physical devices or human observers, and the subsequent processing of data by software or humans, can introduce errors and bias.^59^ For instance, pulse-oximeter devices have been shown to overestimate blood oxygen saturation in individuals with darker skin.^60^ Where specific biases are known or suspected in the acquisition or processing of data, this bias should be described and explained in the dataset documentation. Where possible, metadata should be provided for each variable in a dataset, stating how it was acquired, including details of any devices, sensors, and software used. Digital Imaging and Communications in Medicine and other relevant data standards can be used for this purpose. Any known or suspected inconsistency in the way data were acquired or processed between groups should be clearly highlighted as a source of bias. Within particular variables, differences in the degree and nature of uncertainty between groups in the dataset should be highlighted (eg, some groups might have access to more accurate or precise versions of a particular laboratory test; the data provided by these more accurate or precise tests will have a level of uncertainty different from that of data derived from less accurate or precise tests). If any attempts have been made to mitigate these biases, these should be described (see also item 1.3a).

### Recommendation 1.3e: known or potential exclusion introduced by data collection

Dataset documentation should: (1) Identify the context of data collection and areas where exclusion may have been introduced into the data collection process. (2) Describe any attempts to mitigate these biases (appendix pp 23–24).

#### Explanation

When individuals are inadequately represented in datasets, they are less likely to be able to benefit from subsequent health technologies developed with the data.^19^ When data collection is known or suspected to have excluded certain groups, this exclusion should be explained (eg, collecting data from only one geographical area, including only participants with access to certain health-care services or providers, or using questionnaires in particular languages). Any attempts to mitigate exclusion should also be explained (see also items 1.2a, 1.3a, and 1.3b). Differences in the availability of data between groups represented in a dataset should be reported, including any likely causes of these differences. For instance, if it were noted that individuals in one ethnic group had fewer scan results than other ethnic groups, this difference in number could have several causes, including barriers to accessing health care, clustering of particular communities in geographical areas and divergent practice between health-care institutions, or use of risk-estimation tools that use ethnicity as a predictor. The implications of these differences on the reliability of the data should also be reported.

### Recommendation 1.3f: known or potential bias in assigned or derived labels

Dataset documentation should: (1) Provide a description of any assigned or derived labels, including who decided what labels to include, what they were called, and how they were generated. (2) Highlight labels that are at high risk of bias; for example, where label generation was at the discretion of individuals, where known biases in labelling behaviour have been evidenced previously, or in the use of proxy variables. (3) Describe any attempts to mitigate these biases (appendix p 25).

#### Explanation

Labels refer to annotations or information assigned to each data instance, to provide meaning to the raw data. Labels are commonly used in supervised learning tasks, when associations are learned between features within the data and their corresponding labels. Labels can be generated by subjective assessment by human experts (eg, radiological image interpretation), derivation of parameters using proxy variables (eg, estimating health-care need using the proxy variable of health-care cost), instrument-based measurement of a biological process (eg, diagnosing a myocardial infarction using elevated troponin concentration), using an algorithm trained with a relevant dataset, or objective measurement (eg, height and mass). All types of labels can be subject to bias, whether through conscious or unconscious human bias, differential performance of instruments between groups, or because of encoded bias in an algorithm used for labelling. If any attempts have been made to mitigate bias in labels, the rationale and methods used should be described (see also item 1.3a).

### Recommendation 1.4a: ethics and governance

Dataset documentation should: (1) State which data protection laws have been adhered to, and in which jurisdiction(s) they apply. (2) Describe measures taken to protect the identities of individuals. (3) Describe permissions (including ethical, legal, and institutional) obtained to enable dataset creation, and details of the governance of the dataset. (4) Describe adherence to principles that respect data sovereignty for communities, where relevant (appendix pp 25–26).

#### Explanation

Datasets must adhere to ethical principles that underpin health care and comply with data protection and privacy laws relevant to the jurisdiction from which data are obtained. The identities of individuals should be protected in accordance with data protection laws and any assurances given to the individuals themselves, and dataset documentation should clearly describe measures taken to ensure this. Relevant permissions should be obtained before providing access to or releasing a dataset, including from ethical, legal, and institutional review boards or equivalent. Details of these permissions, including any reference numbers, the approving body, and the date of approval, should be provided in dataset documentation. If conducted, details of any review by data safety monitoring boards (or equivalent) should also be provided. Where reference standards or guidance for dataset governance have been adhered to (eg, the International Organization for Standardization or WHO),^61^ details should be given. Data sovereignty refers to ethical and legal frameworks that aim to enable and respect the self-determination of communities (most notably Indigenous communities) to manage, use, and protect data about their own people. Examples of data sovereignty frameworks include Collective benefit, Authority to control, Responsibility, Ethics and Ownership, Control, Access, Possession.^62^, ^63^

### Recommendation 1.4b: patient and public participation

Dataset documentation should: (1) Describe the role of any advisory boards and patient and public participation groups. (2) Provide information on any efforts to share data and findings with those who contributed to the dataset and any feedback gathered from participants that is relevant to data interpretation (appendix pp 26–27).

#### Explanation

It is important to promote free, active, and meaningful participation of patients and the public when creating datasets and making them available for use (ie, participation).^56^, ^64^ The roles of patient and public representatives and advisory boards in dataset creation and sharing should be described, including efforts to share the outputs of datasets with contributors of the data. In some cases, this might be deliverable only at an aggregate level for all individuals together—for instance, publishing plain language summaries of research findings on a dataset's website. In other cases, data itself can be shared with individuals, either for information purposes or to provide an opportunity for individuals to check alignment with how they perceive they should be represented. If no such sharing of data or of findings has been done, this should be stated in the dataset documentation and reasons given (eg, because of technical difficulty or cost constraints).

### Recommendation 1.4c: bias and impact assessments

If a formal assessment of bias or societal impact has been previously conducted on the dataset, dataset documentation should include these assessments and results (appendix p 28).

#### Explanation

Formal assessments of bias or societal impact can include (but are not limited to) equality impact assessments (to encourage identification of discrimination and barriers to equality), algorithmic impact assessments (to assess potential societal impacts of an algorithmic system before the system is in use),^65^ data protection impact assessments (to identify and minimise data protection risks),^66^ and disclosure risk assessments.^67^ Where possible, the dataset documentation should include either the assessment itself, a summary of its key points and conclusions, or a permanent link to the site where it is available.

## Recommendations for the Use of Health Datasets

Development and testing of AI health technologies requires the use of data. AI health technologies should be developed and evaluated using datasets that represent the intended purpose and intended use population.

Here we present 11 recommendations for dataset use, aimed at data users, including the following: provision of sufficient dataset documentation (item 2.1a), evaluation of datasets and AI health technologies in the context of relevant groups (items 2.2a–2.2f), acknowledgment of known biases and limitations, and their implications (2.3a–2.3c), and management of uncertainties and risks when using datasets throughout the lifecycle of AI health technologies (2.4a).

### Recommendation 2.1a: provide sufficient information about dataset(s) to allow traceability and auditability

Datasets used in the lifecycle of AI health technologies should be accompanied by documentation which conforms to Recommendations for Documentation of Health Datasets (see previous section), enabling auditing against these statements (appendix p 29).

#### Explanation

The lifecycle of an AI health technology includes idea generation, all stages of development and regulation, implementation, and ongoing post-market data collection that could lead to iteration or withdrawal of the technology. All datasets used during the lifecycle of the AI health technology should be accompanied by adequate documentation that conforms to the Recommendations for Documentation of Health Datasets (see previous section). Transparency, as described in this item, is essential for regulators, purchasers, and users to be able to robustly evaluate performance of AI health technologies, including with regard to safety and potential bias. This transparency includes a detailed understanding of the datasets used throughout the product lifecycle, particularly those used in the later stages of development. It is recognised that this transparency might be in tension with some other considerations of dataset users (such as commercial considerations). However, even if datasets themselves are not shared, provision of descriptive summaries of the data is valuable and can be a helpful mechanism to improve safe use of AI health technologies. Additionally, dataset users might be using datasets for which the original descriptions are themselves incomplete. When documentation cannot be provided (for these or other reasons), this should be stated and explained.

### Recommendation 2.2a: identify contextualised groups of interest in advance who may be at risk of disparate performance or harm from AI health technology

Data users should identify contextualised groups of interest in advance. These may be identified in various ways, including evidence appraisal and literature review, collaboration with domain experts in the intended use of the AI health technology, consultation with those who have relevant lived experience, and evidence generation and discovery through data analysis and algorithm testing (appendix pp 30–31).

#### Explanation

Contextualised groups of interest consist of individuals with shared relevant attributes with known or suspected associations with disparate health outcomes relating to the intended use of the AI health technology. Disparate health outcomes can be driven by a variety of factors, including structural and social determinants of health, biological factors, or by the AI health technology under development (including datasets used and the effect of implementation on the health-care pathway).^7^, ^42^ Predefining these groups can help developers and evaluators of AI health technologies to make informed decisions around appropriate inclusion, testing, and auditing of the performance of the technology across these groups, to reveal potential disparate performance of the technology.^23^ Individual patients can be personally harmed by an AI health technology (eg, by misclassifying a chest x-ray showing pneumonia as being healthy, or by influencing a clinician to recommend the wrong treatment for the patient, including by disregarding the testimony of the patient in favour of the prediction of an algorithm). Harm can also be indirect or at the group level (eg, an AI health technology that causes or contributes to health inequity by exhibiting disparate performance, in which certain groups experience greater improvements in health outcomes than others). Contextualised groups of interest can be identified in various ways, including with reference to intersectionality. It should be recognised that no single source of knowledge is comprehensive, and this activity is not intended to be an exhaustive search for all possible groups. In some cases, groups at risk of harm might have been identified in the documentation accompanying datasets used (see item 1.2c).

### Recommendation 2.2b: justify that datasets have been used appropriately to support the intended use population and intended use of AI health technology

The intended use population should be appropriately represented in datasets used in an AI health technology. The contextualised groups of interest (ie, those who may be at risk of disparate performance or harm; see item 2.2a) should also be included where possible, and if not included this should be explicitly stated by data users. Areas of under-representation should be identified and transparently reported by data users (appendix p 32).

#### Explanation

As part of seeking regulatory authorisation for a medical device, the applicant is usually required to specify the intended use population within the intended use statement (also known as the intended purpose statement).^46^ In doing so, the manufacturer should provide evidence that performance for the intended use population is safe. If the previously defined contextualised groups of interest are not appropriately represented in the datasets used to develop or evaluate an AI health technology, this should be clearly stated and both the reasons for under-representation and a plan to mitigate any consequences should be explained.

### Recommendation 2.2c: report the explicit and implicit use of relevant attributes during the lifecycle of the AI health technology

Data users should report whether and how any relevant attributes were used during the lifecycle of the AI health technology, including as a feature, proxy, or label (appendix p 33).

#### Explanation

Some AI health technologies use relevant attributes explicitly or implicitly. This inclusion might be as a feature or predictor (eg, sex as a predictor in a multivariable model for stroke risk), a proxy for another measure (eg, health-care cost as a proxy of health-care need), or a label (eg, prediction of sex using retinal images). The decision to use such attributes is often built on assumptions about how the attribute is associated with the health outcome of interest. These assumptions might be shown to be flawed, misunderstood, or disproven as new evidence comes to light. By reporting the explicit use of such attributes, the scientific basis of these associations can be scrutinised.

In some cases, the attributes of individuals can be associated with a disease because of biological factors (eg, the gene causing sickle cell disease is particularly prevalent in Black people with ancestry in sub-Saharan Africa). However, in many cases, the assumed correlation between certain diseases and race or ethnicity is sometimes more accurately explained by their exposure to social determinants of health (eg, in settings in which individuals in certain racial or ethnic groups experience greater deprivation, or face direct discrimination, oppression, and violence, these factors drive ill health rather than race or ethnicity per se). In such cases, the relationship between relevant attributes (including race and ethnicity) represents a social bias and use of these attributes in the AI health technology lifecycle risks perpetuating this bias. Of particular concern is the possibility that the relevant attribute might, as a result of the model, become linked to the allocation of resources (eg, if the race or ethnicity of an individual affects their predicted illness severity this could affect clinical decisions about subsequent escalation to critical care). It should be noted that even when a relevant attribute, such as race or ethnicity, is not intentionally used within the model, the use of other data that are strongly associated with the relevant attribute (eg, insurance status) might cause the same bias. Given these risks of bias, data users should report and justify the use of any relevant attributes for any purpose in the AI health technology lifecycle, including any potential implications.

### Recommendation 2.2d: evaluation performance of the AI health technology for contextualised groups of interest

Data users should report performance of the AI health technology for contextualised groups of interest identified in item 2.2a, to enable comparison of performance for each group versus aggregate performance across the overall study population, and comparison of performance between different contextualised groups of interest (appendix p 34).

#### Explanation

Disparate performance between the contextualised groups of interest should be transparently reported to enable appropriate decision making regarding potential risk of harm. Performance should be reported for all contextualised groups of interest, and comparison should be made between these groups and between each group and the overall performance in a study population, to provide a relative comparison of the magnitude of risk and to highlight in which groups the differences are greatest. The level of uncertainty should also be reported using appropriate statistical methods (eg, with confidence intervals). If it is not possible to compute performance for certain contextualised groups of interest because of a complete absence of data, this should be explained.

### Recommendation 2.2e: identify disparate performance in any additional groups outside of the prespecified contextualised groups of interest

As well as conducting prespecified evaluation of performance in contextualised groups of interest (items 2.2a and 2.2c), data users should also evaluate performance across other groups to identify disparate performance of the AI health technology which was not previously anticipated, and may lead to harm (appendix p 35).

#### Explanation

The purpose of this exploratory analysis is to identify groups for which the AI health technology exhibits disparate performance so that the risk of harm when the technology is used can be assessed. In addition to the prespecified analysis (across contextualised groups of interest), there might be other groups that experience disparate performance of the AI health technology. The observed performance of the AI health technology should be compared across all other available groups; detection of substantial disparities in performance between groups could indicate algorithmic bias. As well as reporting performance for these groups, the level of uncertainty should also be reported using appropriate statistical methods (eg, with confidence intervals). Developers, evaluators, and auditors of the AI health technology should determine whether any disparity or uncertainty in performance is sufficient to warrant further investigation.

The effect of data shifts over time could mean that groups at risk of harm change over time and between contexts. Performance of the AI health technology might also change over time, meaning that repeated evaluation is needed. Groups at risk of harm can be identified through automated approaches (eg, clustering analyses), or manually by considering a longlist of attributes, including (but not limited to) sex, gender, race, ethnicity, age, socioeconomic status, sexual orientation, disability, pregnancy, relationship or marriage status, religion or belief, nationality, ancestry, occupation, language or languages spoken, caste, creed, or tribe. Depending on the context, it might be appropriate to consider evaluation in intersectional groups (eg, young female individuals).

### Recommendation 2.2f: report any approaches or methods (including fairness methods) used to intentionally modify performance across groups

Data users should document any approaches or methods used during the lifecycle of the AI health technology which attempt to modify performance, including addressing disparate performance across groups. If applicable, explain the rationale and goals for doing so, the methods and metrics used, whether and how thresholds were set and whether these varied between groups (appendix p 36).

#### Explanation

If the AI health technology was modified with the intention of altering performance across groups, or to use differences between groups to compensate for pre-existing inequities (eg, applying different thresholds or recalibration across groups), this should be documented. Documentation should include the rationale for doing so and should also detail the methods applied and the results achieved.^23^ We neither endorse nor recommend the use of fairness methods because the validity of such approaches is recognised as an ongoing area of research.

### Recommendation 2.3a: report limitations of datasets used and any implications on the AI health technology

Data users should report the limitations of datasets used, and any implications with reference to the intended use of the AI health technology. Data users should investigate whether these limitations are systematically different across relevant groups, including those with attributes categorised as “unknown”, “prefer not to say”, or “other”, and report differences which could result in disparate performance of the AI health technology across groups (appendix p 37).

#### Explanation

Data users should appraise datasets they use during the lifecycle of AI health technologies and report any limitations. Transparency should be prioritised, such that any and all limitations are reported, even if their implication in relation to the intended use is minor. The choice of relevant groups should be justified, but would be expected to include contextualised groups of interest and groups identified in item 2.2e.

### Recommendation 2.3b: report differences between the intended purposes of the AI health technology and datasets used, including the implications of discordance

Data users should report the purpose(s) of datasets used (see item 1.1c), and how these differ from the intended use of the AI health technology (see 2.2b). The implications of any discordance and how this affects the suitability of the dataset for its role should be stated (appendix p 38).

#### Explanation

Although datasets created for a particular purpose can have several other valid uses, data users should ensure that the choice of datasets is appropriate for the intended use of the AI health technology (see item 2.2a and 2.2b). For transparency, data users should report the intended purposes of datasets used and the implication of any discordance with the intended use of the AI health technology, particularly when this could contribute to bias. Transparency should be prioritised, such that any and all implications are reported (eg, a dataset generated during a clinical trial will be influenced by the eligibility criteria of the trial and might not represent the wider population, a bias that might affect the performance of any algorithm developed using these data).

### Recommendation 2.3c: report findings from pre-existing assessments of the AI health technology and any datasets used

Data users should review any available pre-existing assessments of both the AI health technology and any datasets used, and report how the findings may have implications on groups within the intended use population, including risk of harm (appendix p 39).

#### Explanation

Formal assessments of bias or societal impact can include (but are not limited to) equality impact assessments, algorithmic impact assessments,^65^ medical algorithmic audits,^68^ and data protection impact assessments.^66^ If any of these parameters include information relevant to the performance of the AI health technology between different groups within the intended use population, this should be reported. Harm in this context refers both to direct harm caused by the AI health technology and to indirect harm (including causing or exacerbating health inequity).

### Recommendation 2.4a: address uncertainties and risks with mitigation plans

Where data users have identified uncertainty or potentially variable performance in groups, any clinical implications resulting from these findings must be clearly stated and reported as risks. The data user should document strategies to monitor, manage, and reduce these risks as part of the implementation of the AI health technology (appendix p 40).

#### Explanation

Data users should assess the potential clinical implications of uncertainty or disparate performance of the AI health technology across groups and over time. The mitigation of risks associated with the AI health technology is likely to require collaboration from many stakeholders; however, the data user has a specific responsibility to address this as they are developing the AI health technology.^69^, ^70^ When the AI health technology is a medical device, the data user will have certain legal obligations to document and mitigate many of these risks and potentially to report serious adverse incidents and events to the relevant regulator. Before deployment, the data user should therefore prespecify strategies to monitor, manage, and reduce risks, including post-market surveillance and (where appropriate) post-market clinical follow-up, including in the event that risk of harm differs between groups.

## Discussion

Ethical delivery of health care requires prioritising not only safety but also inclusivity and health equity. In the context of AI, these considerations apply both to the technologies themselves and to the health systems in which they are deployed.^61^ Health datasets can be a major contributor to AI-driven health inequalities both through the absence of diversity and inclusion and because of embedded biases. STANDING Together has sought to tackle this problem through an extensive, international, multistakeholder Delphi study, building consensus-driven Recommendations for Transparent Documentation of Datasets and harm mitigation for algorithmic bias. Our study includes the following key strengths: the iterative design, engaging a large group of stakeholders to identify and refine potential items, elicit dissenting views, and respectfully build consensus where possible; the diversity of the participant group by role (patients and the public, health professionals, technologists, data curators, ethicists, policy experts, academic researchers, journal editors, and medical device regulators), by geography (58 countries), and by demographics; the number of participants reached; and the emphasis on ensuring that recommendations are specific enough to be operationalisable, but also sufficiently sensitive and flexible enough to be applicable across different contexts and settings. Through our public consultation and wider engagement, we have transparently communicated progress and encouraged challenge and dissent throughout the development of these recommendations.

This Review builds on existing published work evidencing biases and patterns of health inequalities embedded within data, and the consequences on algorithmic bias and downstream health outcomes. Earlier studies led to the following key findings: systematically poor diversity and representation in health datasets across several disease areas and a general absence of transparency about who is represented;^10^, ^11^, ^12^, ^13^, ^14^ under-representation within training datasets leading to disparate performance and to higher uncertainty in model predictions due to model instability, and under-representation in test datasets leading to higher uncertainty in estimates of model performance;^71^ the potential for groups to be made invisible through data aggregation, homogenisation, or other forms of categorisation that remove granularity in individual attributes;^72^ and emerging evidence of downstream harm.^3^ These findings have led to the development of tools that advocate for reflective approaches to dataset construction and transparent reporting.^31^ Notably, many of the items in Recommendations for Documentation of Health Datasets were built from Healthsheet (a documentation artifact for health datasets) and Datasheets for Datasets.^27^, ^28^ Both include additional considerations for dataset documentation, beyond bias and equity considerations, and we recommend their use alongside the STANDING Together recommendations.

This study additionally responds to the needs of international policy makers and regulators. WHO, regulators in several jurisdictions, and other policy bodies have all issued statements regarding a need for AI health technologies to be built on inclusive, representative data.^61^ However, there has hitherto been a lack of detail as to what good looks like in a health-care context. Through the consultations of this study, regulators and other stakeholders noted challenges in knowing where to set the bar of acceptable practice, how to define the axes on which inequity and bias should be considered, and how to operationalise a set of principles across many contexts in which AI health technologies are built and implemented.

Several themes emerged through this study, including the role of proactive inquiry and of transparent reporting as iterative drivers for better practice, the need to be sensitive to context and complexity, and the value of a shared understanding of what we are aiming for (even when it might not yet be achievable).

Choices made during the creation of datasets include the recording of the attributes of individuals and selection of data sources to be aggregated. These choices, which can be conscious or subconscious, often reflect the worldview of those generating and processing health data or funding and commissioning dataset creation. The Recommendations for Documentation of Health Datasets prompt dataset curators to reflect broadly on the types of bias present and to include details in dataset documentation. These details include a consideration of how attributes are used to group individuals and the associated value judgements, and how the technical and social processes of measurement, recording, processing, and transmission of health data can introduce bias and the differential patterns of error that might occur.^3^, ^73^ Some biases can occur during the sampling and processing stages, whereas others will be present at source because of structural inequities. The Recommendations for Use of Health Datasets provide actionable guidance on how to translate identification of bias into risk mitigation measures, while aligning to existing medical device regulation requirements. These recommendations call for proactive identification of contextualised groups of interest, representing those who are already evidenced as having (or being at risk of) disparate health outcomes. They also ensure that disparate performance across such population groups is specifically tested for and transparently reported. Finally, they recommend that the implications of performance disparity should be reported as risks, and mitigation plans developed to reduce the risk of harm. Regardless of the nature of bias, its identification and transparent reporting can enable data users to reduce the risk that it is encoded in downstream AI health technologies. If bias encoding cannot be avoided at the algorithm stage, its identification enables a range of stakeholders relevant to the AI health technology's use (developers, regulators, health policy makers, and end users) to acknowledge and mitigate the translation of bias into harm.

The STANDING Together programme was originally funded to address health inequity driven by bias relating to race and ethnicity. However, while defining the scope of this work, it became increasingly clear that relevant attributes are wide-ranging, can intersect, and are often highly context and culture dependent. We therefore took a principle-based approach, avoiding the listing of specific attributes, and have provided flexibility for users to define attributes that are most relevant when considering health inequalities in a given context. The diversity of all members of the STANDING Together collaboration—by geography, culture, and role—has been crucial in developing recommendations that balance specificity and clarity, while being flexible enough to be contextualised to different settings.

Although the recommendations arising from STANDING Together are intended to serve a range of audiences, the outputs have been intentionally positioned to ensure that they can support medical device regulators. In 2021, the US Food and Drug Administration, UK Medicines and Healthcare products Regulatory Agency (MHRA), and Health Canada published joint guidelines for Good Machine Learning Practice (GMLP), which include the guiding principle that datasets are representative of the intended patient population.^25^ It is our intention that the STANDING Together recommendations will support regulators and AI health technology manufacturers in meaningfully aligning with guidelines and principles including GMLP, and support them in providing evidence to demonstrate this alignment. The STANDING Together programme is also part of the MHRA Software and AI as a Medical Device Change Programme Roadmap, as a tool to identify bias.^74^

The STANDING Together recommendations reflect a consensus position of data participants (patients and public representatives), dataset curators, data users, and institutions that influence decisions (eg, medical device regulators, journal editors, and health research funders). This consensus position required all stakeholders to understand the competing priorities—for example, the concern of dataset curators and innovators to minimise burden and provide flexibility alongside the desire to mandate specific and clear targets across an exhaustive list of risk areas. An unexpected but crucial part of building this shared understanding was the need to ensure that the group was using a common language, by both agreeing definitions of existing terms and introducing new terms when current terms were deemed to be inadequate (eg, relevant attributes and contextualised groups of interest). The recommendations are complementary to checklists that facilitate transparent reporting of research studies, such as those covering a range of study types including randomised controlled studies (CONSORT-AI), early-stage clinical evaluation (DECIDE-AI), and diagnostic and prognostic prediction model studies (TRIPOD+AI).^75^, ^76^, ^77^

It is our hope that the STANDING Together recommendations are about culture change rather than checklists. They are a set of principles based on a shared understanding, which will guide those creating and using health datasets to do so in a way that minimises harm. The principles are designed so that they invite users of these recommendations to respond with a reflective statement on how they have addressed each item, rather than with a binary yes or no. There is no perfect dataset, and the first crucial step is to recognise this. If specific parts of the recommendations cannot be met, this should not be perceived as a deficiency of the dataset; instead, transparency through detailed documentation of limitations of the dataset should be seen as a strength and added value. In so doing, individuals and teams creating and using datasets are better able to identify key factors that might drive bias and subsequent risk of harm, and reflect on what can be done to combat this in future.

In line with the ethos of this work, the STANDING Together team has aimed to bring an approach of proactive, self-critical inquiry throughout the design and delivery of the study, including with regard to assessing probable impact and plans for dissemination. Limitations related to the study itself include factors that might limit inclusion of participants and therefore lead to bias in the sample represented. Although the study reached 58 countries, this should be seen in the context of, for example, 193 member states of the UN. In addition, the predominant use of electronic means of communication and the English language will have excluded some potential participants. We acknowledge these limitations and will include in our dissemination of these recommendations a way to gather further feedback from a broader reach than was achieved through their development, with a view to steadily increasing representation in future iterations of these recommendations.

Limitations related to the study delivery include several factors common to consensus studies: omission of relevant publications that post-date the systematic review; possible survey fatigue in which Delphi respondents simply accept most items rather than engage with them critically; and risk of strong voices exerting undue influence during the consensus meeting, undermining the careful neutrality of the earlier Delphi stages. In response, it should be noted that we actively identified, and sought to minimise, these risks through steps that included the following: frequent scanning of new literature throughout the study and through consultation with our working group; accessible survey design with visual aids to minimise respondent burden; and active measures in the consensus meeting to ensure that less dominant voices felt empowered to speak and be heard, and ensuring that all decisions at the consensus meeting were taken in the context of the earlier findings of the (anonymous) Delphi study.

Several considerations relating to the societal context in which the STANDING Together recommendations currently reside are likely to affect its readiness for adoption, dissemination, and impact. STANDING Together requires support from regulators, journal editors, funders, standards bodies, and other institutions that can endorse and incorporate these recommendations into regulatory policy. Although the recommendations have value across the data lifecycle, it is recognised that, particularly in the early development of AI health technologies, it might be impractical or impossible to adhere to all aspects of the recommendations. When adherence is not possible, we advocate that this should be documented and explained. It is also recognised that adoption of these recommendations requires time and resource investment, and for small teams and individual researchers, finding these resources might be particularly challenging. We therefore advocate for funders to recognise the importance of STANDING Together practices and ensure that research teams can be adequately resourced to deliver on its recommendations.

Cutting-edge AI technologies (including large-scale foundation models) could enable far more complex and capable AI health technologies, but with risks that are unknown and might be of far greater scale. Although the STANDING Together recommendations were not built with these technologies specifically in mind, we believe that they have general relevance, although updates might be needed as these technologies mature. It is also recognised that sensitive documentation requirements, particularly for minoritised groups already experiencing oppression, discrimination, and in some cases, prosecution, could put individuals at greater risk of harm. Moreover, between countries and cultures, there will be differences in the groups that are underserved or that experience minoritisation, marginalisation, and oppression. The rights and interests of individuals and groups must therefore be carefully considered in the context of relevant geographies, cultures, and societies. We plan to address these considerations and others in a separate report, detailing a linked qualitative research study focused on the implementation of these recommendations. We do not provide guidance in this Review about the structure that dataset documentation should take. Instead, we invite those using these recommendations to consider existing artefacts when structuring their documentation, including Datasheets for Datasets or Healthsheets. Medical journals and other organisations can opt to design bespoke artifacts to best fit their needs.^27^, ^28^ At the time of publication, the recommendations will be available only in English, but we are committed to increasing dissemination through translations. Finally, although these recommendations are intended to reduce the risk of AI health technologies contributing to health inequity, they cannot address the underlying social causes of health inequity.

The STANDING Together recommendations, focused on data diversity and inclusion, aim to equip dataset curators and users to better understand the limitations of the datasets of today and improve the datasets of tomorrow as a crucial foundation for building inclusive and equitable AI health technologies. It is anticipated that these recommendations will need amendment and updating as our collective understanding of this complex area develops, through feedback from users, researchers, regulators, and other policy makers and the public. We invite comments and suggestions via the STANDING Together website to ensure that future versions are as broadly applicable as possible, while enabling standardisation where possible. We particularly welcome the work of others to contextualise these recommendations for their own settings, and we ask that they openly share their experiences and adaptations to accelerate learning and improvement in practice, with a view that the potential benefits of AI transformation in health care can be shared more inclusively and equitably.

### Contributors

### Data sharing

A detailed summary of the way in which each individual item was modified during development of these recommendations is provided in the appendix (pp 10–43). This summary includes the performance of each item across all rounds of the Delphi study. Anonymised raw data from each Delphi voting round and the r code used to generate plots and summary statistics can be requested for the purpose of verifying the findings of this research via an email sent to the corresponding author. Data relating to questions that required free-text responses and those relating to demographic attributes will be redacted. Other relevant study documentation (specifically, the wording of the questions asked across all three Delphi survey rounds) are provided in the appendix (pp 44–154).

## Declaration of interests

JEA is a named researcher on grants funded by the Engineering and Physical Sciences Research Council (EPSRC), the National Institute for Health and Care Research (NIHR), and the Medical Research Council (MRC); is co-organiser of the Alan Turing Institute Clinical AI Interest Group; and was an NIHR Academic Clinical Fellow from 2017 to 2020. EL is employed as a research fellow at University Hospitals Birmingham NHS Foundation Trust and received support to attend the Symposium for Artificial Intelligence Learning Health Systems conference (May, 2023). JO is an employee of Roche Diagnostics but contributed while an employee of the UK MHRA; declares support from the AIRIS 2024 committee; and has shares in Roche Group. MG is a Canadian Institute for Advanced Research (CIFAR) AI Chair, CIFAR Azrieli Global Scholar, Herman L F von Helmholtz Career Development Professor, and Jameel Clinic Affiliate, and acknowledges support from these programmes. SRP is an employee of Google Research and has stock in Google. JS is an employee of Harrison.ai. NR is an employee of Google Research. HC-L is an employee of Google and has stock or stock options in Google. DS is Acting Deputy Editor of *The Lancet Digital Health*, Elsevier. BG was scientific advisor for Kheiron Medical Technologies (January, 2018, to September, 2021) and has had part-time employment with stock options as part of the standard compensation package (since October, 2021); has part-time employment at HeartFlow with stock options as part of the standard compensation package (since September, 2018); and is the Royal Academy of Engineering Research Chair in Safe Deployment of Medical Imaging AI. MCa is Director of the Birmingham Health Partners Centre for Regulatory Science and Innovation, Director of the Centre for Patient Reported Outcomes Research, and an NIHR Senior Investigator. MCa, NIHR Birmingham Biomedical Research Centre, Applied Research Collaboration West Midlands, UK SPINE, Research England, the European Regional Development Fund DEMAND Hub at the University of Birmingham, the University Hospitals Birmingham NHS Foundation Trust, and the NIHR Blood and Transplant Research Unit in Precision Transplant and Cellular Therapeutics; funding from Health Data Research UK, Innovate UK (part of UKRI), Macmillan Cancer Support, UCB Pharma, GSK, Anthony Nolan, Gilead Sciences, European Commission, European Federation of Pharmaceutical Industries and Associations, Janssen, Merck, and The Brain Tumor Charity; and personal fees from Aparito, ICON, CIS Oncology, Takeda Pharmaceuticals, Merck, Daiichi Sankyo, Glaukos, GSK, Halfloop, the Patient-Centered Outcomes Research Institute (PCORI), Pfizer, Genentech, and Vertex Pharmaceuticals, outside of the submitted work. MCa also declares royalties via revenue share from the commercial licence of Symptom Burden Questionnaire-Long COVID; payment or honoraria from the University of Maastricht, South-Eastern Norway Regional Health Authority, Cochrane Portugal, Singapore National Medical Research Council; and stock in GSK held by a family member. MCa has a leadership or fiduciary role in PROTEUS consortium (paid via a consultancy fee from Genetech and PCORI). TJP declares NIH awards (OT2OD032701 and R01EB030362), and grants or contracts from Google Cloud and Amazon Web Services. SK was an employee of Hardian Health. L-AF received consulting fees from Therapeutic Goods Administration, Australia, and WHO, and is a member of the AI in Healthcare Committee for Standards Australia and Director of Tethyan Consulting. CS received research funding from UKRI, NIHR, and the Wellcome Trust to support their research programme. BAM reports grants or contracts from MRC, the British Heart Foundation, the United States Agency for International Development, and HDRUK; consulting fees and support for attending meetings from the Bill & Melinda Gates Foundation; and was an employee of the Wellcome Trust at the time of the Delphi study. KH is an employee of Google Research. AK is an employee of Google and receives Google stock reimbursement. MM was an employee of Genomics England, Founder and Director of One HealthTech and Data Science for Health Equity (within One HealthTech), and received support from Nature Journals and Conde Nast. RP is an employee of MHRA. AKM is Deputy Editor of *NEJM AI*, NEJM Group. PM has a data custodian role as Director of the Clinical Practice Research Datalink and is an employee of MHRA. ZK was employed by Health Research Authority at the time of the consensus process. AAk received grants from the Economic and Social Care Research Council (ADR Wales ES/W012227/1) and HDRUK (HDR-9006). LB received support from the US National Institutes of Health (NIH) All of Us Research Programme to attend STANDING Together meetings. JWG is on the 2022 Robert Wood Johnson Foundation Harold Amos Medical Faculty Development Programme; declares support from the Radiological Society of North America Health Disparities grant (EIHD2204), Lacuna Fund (67), Gordon and Betty Moore Foundation, and NIH (NIBIB) Medical Imaging and Data Resource Center grant under contracts 75N92020C00008 and 75N92020C00021; has received honoraria from the National Bureau of Economic Research for authorship in their 2022 conference collection; has participated on the American Heart Association Deracing Algorithms advisory board; and is a board member of Hl7 and SIIM. LR is Senior Editor of the journal *Nature Medicine*, Nature Research, Springer Nature. MCh is an NIHR Academic Clinical Fellow. AAr is a panel member for various NIH Research committees, has received honoraria from HDRUK for attending panel meetings, and has an honorary affiliation with Moorfield's Eye Hospital. SP is employed by WHO. ES has had research funded by UK Research and Innovation (UKRI), NIHR, Wellcome Trust, Health Data Research UK (HDRUK), MRC, EPSRC, and Innovate UK, and declares support from the European Respiratory Society, British Thoracic Society, and Gilead. VT is Head of UK Approved Body as at the British Standards Institution. AD reports institutional research grants awarded by the NIHR, MRC, and EPSRC, and is an NIHR Senior Investigator, Director of the UK's incubator for Regulatory Science in AI and Digital Healthcare, deputy director of the Centre for Regulatory Science and Innovation, Birmingham Health Partners, system lead for AI diagnostics to NHS England, and a member of the UK Government's Regulatory Horizons Council. XL reports funding from the NIHR, the UK National Health Service AI Lab, The Health Foundation, Research England, the MHRA, the National Institute for Health and Care Excellence, and Hardian Health, and was previously a Health Studies Scientist at Apple; reports grants from the Medical Research Council, Wellcome Trust, NIHR, and Moorfields Eye Hospital Charity; received payment or honoraria for talks and book chapter reviews from the University of Turku and Elsevier; and received support from Harvard Medical School, SingHealth, and The Ada Lovelace Institute. All other authors declare no competing interests.

## References

[1] US Food and Drug Administration (2023). Artificial intelligence and machine learning (AI/ML)-enabled medical devices. https://www.fda.gov/medical-devices/software-medical-device-samd/artificial-intelligence-and-machine-learning-aiml-enabled-medical-devices.

[2] Muehlematter UJ, Daniore P, Vokinger KN (2021). Approval of artificial intelligence and machine learning-based medical devices in the USA and Europe (2015–20): a comparative analysis. Lancet Digit Health.

[3] Obermeyer Z, Powers B, Vogeli C, Mullainathan S (2019). Dissecting racial bias in an algorithm used to manage the health of populations. Science.

[4] Seidenberg AB, Moser RP, West BT (2023). Preferred reporting items for complex sample survey analysis (PRICSSA). J Survey Stat Methodol.

[5] Seyyed-Kalantari L, Zhang H, McDermott MBA, Chen IY, Ghassemi M (2021). Underdiagnosis bias of artificial intelligence algorithms applied to chest radiographs in under-served patient populations. Nat Med.

[6] Wornow M, Xu Y, Thapa R (2023). The shaky foundations of large language models and foundation models for electronic health records. NPJ Digit Med.

[7] Chen IY, Pierson E, Rose S, Joshi S, Ferryman K, Ghassemi M (2021). Ethical machine learning in healthcare. Annu Rev Biomed Data Sci.

[8] Lee T, Puyol-Antón E, Ruijsink B, Aitcheson K, Shi M, King AP, Wesarg S, Puyol Antón E, Baxter JSH (2023). Clinical image-based procedures, fairness of AI in medical imaging, and ethical and philosophical issues in medical imaging.

[9] Larrazabal AJ, Nieto N, Peterson V, Milone DH, Ferrante E (2020). Gender imbalance in medical imaging datasets produces biased classifiers for computer-aided diagnosis. Proc Natl Acad Sci USA.

[10] Khan SM, Liu X, Nath S (2021). A global review of publicly available datasets for ophthalmological imaging: barriers to access, usability, and generalisability. Lancet Digit Health.

[11] Wen D, Khan SM, Xu AJ (2022). Characteristics of publicly available skin cancer image datasets: a systematic review. Lancet Digit Health.

[12] Alderman JE, Charalambides M, Sachdeva G (2024). Revealing transparency gaps in publicly available COVID-19 datasets used for medical artificial intelligence development—a systematic review. Lancet Digit Health.

[13] Laws E, Palmer J, Alderman J (2025). Diversity, inclusivity and traceability of mammography datasets used in development of Artificial Intelligence technologies: a systematic review. Clin Imaging.

[14] Wu J, Liu X, Li M (2024). Clinical text datasets for medical artificial intelligence and large language models—a systematic review. New Engl J Med AI.

[15] Fry A, Littlejohns TJ, Sudlow C (2017). Comparison of sociodemographic and health-related characteristics of UK Biobank participants with those of the general population. Am J Epidemiol.

[16] Celi LA, Cellini J, Charpignon M-L (2022). Sources of bias in artificial intelligence that perpetuate healthcare disparities—a global review. PLOS Digit Health.

[17] Gianfrancesco MA, Tamang S, Yazdany J, Schmajuk G (2018). Potential biases in machine learning algorithms using electronic health record data. JAMA Intern Med.

[18] Office for National Statistics (Jan 16, 2023). Understanding consistency of ethnicity data recorded in health-related administrative datasets in England: 2011 to 2021. https://www.ons.gov.uk/peoplepopulationandcommunity/healthandsocialcare/healthinequalities/articles/understandingconsistencyofethnicitydatarecordedinhealthrelatedadministrativedatasetsinengland2011to2021/2023-01-16.

[19] Ibrahim H, Liu X, Zariffa N, Morris AD, Denniston AK (2021). Health data poverty: an assailable barrier to equitable digital health care. Lancet Digit Health.

[20] Geirhos R, Jacobsen J-H, Michaelis C (2020). Shortcut learning in deep neural networks. Nat Mach Intell.

[21] Gichoya JW, Banerjee I, Bhimireddy AR (2022). AI recognition of patient race in medical imaging: a modelling study. Lancet Digit Health.

[22] Ferryman K, Mackintosh M, Ghassemi M (2023). Considering biased data as informative artifacts in AI-assisted health care. New Engl J Med.

[23] McCradden M, Odusi O, Joshi S (2023). Proceedings of the 2023 ACM Conference on Fairness, Accountability, and Transparency.

[24] Ganapathi S, Palmer J, Alderman JE (2022). Tackling bias in AI health datasets through the STANDING Together initiative. Nat Med.

[25] US Food and Drug Administration, Health Canada, Medicines and Healthcare products Regulatory Agency (2021). Good machine learning practice for medical device development: guiding principles. https://www.fda.gov/medical-devices/software-medical-device-samd/good-machine-learning-practice-medical-device-development-guiding-principles.

[26] Schwartz R, Vassilev A, Greene KK, Perine L, Burt A, Hall P (2022). Towards a standard for identifying and managing bias in artificial intelligence. US National Institute of Standards and Technology. https://www.nist.gov/publications/towards-standard-identifying-and-managing-bias-artificial-intelligence.

[27] Gebru T, Morgenstern J, Vecchione B (2021). Datasheets for datasets. Commun ACM.

[28] Rostamzadeh N, Mincu D, Roy S (2022). Proceedings of the 2022 ACM Conference on Fairness, Accountability, and Transparency.

[29] Block RG, Puro J, Cottrell E (2020). Recommendations for improving national clinical datasets for health equity research. J Am Med Inform Assoc.

[30] David D, James CV (2022). A data infrastructure for clinical trial diversity. New Engl J Med.

[31] Arora A, Alderman JE, Palmer J (2023). The value of standards for health datasets in artificial intelligence-based applications. Nat Med.

[32] Dalkey N, Helmer O (1963). An experimental application of the Delphi method to the use of experts. Management Sci.

[33] Keeney S, Hasson F, McKenna H (2006). Consulting the oracle: ten lessons from using the Delphi technique in nursing research. J Advanced Nurs.

[34] Solar O, Irwin AA (2010).

[35] Shelby R, Rismani S, Henne K (2023). Proceedings of the 2023 AAAI/ACM Conference on AI, Ethics, and Society.

[36] International Organization for Standardization ISO 14971:2019. Medical devices—application of risk management to medical devices. https://www.iso.org/standard/72704.html.

[37] International Organization for Standardization ISO/IEC Guide 63:2019. Guide to the development and inclusion of aspects of safety in International Standards for medical devices. https://www.iso.org/standard/67944.html.

[38] McCradden M, Hui K, Buchman DZ (2023). Evidence, ethics and the promise of artificial intelligence in psychiatry. J Med Ethic.

[39] International Medical Device Regulators Forum (2024). Good regulatory review practices. Principles of labelling for medical devices and IVD medical devices. IMDRF/GRRP WG/N52 final. Edition 2. https://www.imdrf.org/documents/principles-labelling-medical-devices-and-ivd-medical-devices.

[40] US Food and Drug Administration (2021). Code of federal regulations 21 CFR 801.4. Meaning of intended uses. https://www.ecfr.gov/current/title-21/part-801/section-801.4.

[41] UK Government The medical devices regulations 2002 (SI 2002 no 618, as amended). https://www.legislation.gov.uk/uksi/2002/618/regulation/2/made.

[42] Health Canada (July 16, 2015). Guidance document: guidance for the labelling of medical devices, not including in vitro diagnostic devices. Appendices for the labelling of soft, decorative, contact lenses and menstrual tampons. https://www.canada.ca/en/health-canada/services/drugs-health-products/medical-devices/application-information/guidance-documents/guidance-labelling-medical-devices-including-vitro-diagnostic-devices-appendices.html.

[43] Therapeutic Goods Administration (July 8, 2023). Federal register of legislation. Therapeutic goods (medical devices) regulations 2002. https://www.legislation.gov.au/F2002B00237/2023-07-01/text.

[44] Crenshaw K (1989). Demarginalizing the intersection of race and sex: a Black feminist critique of antidiscrimination doctrine, feminist theory and antiracist politics. University of Chicago legal forum. https://chicagounbound.uchicago.edu/uclf/vol1989/iss1/8.

[45] Bowleg L (2012). The problem with the phrase women and minorities: intersectionality—an important theoretical framework for public health. Am J Public Health.

[46] International Organization for Standardization ISO/IEC 11179-1. 2023. Information technology—metadata registries (MDR). Part 1: framework. https://www.iso.org/standard/78914.html.

[47] International Organization for Standardization ISO 26324:2022. Information and documentation—digital object identifier system. https://www.iso.org/standard/81599.html.

[48] International Organization for Standardization ISO 24495-1:2023. Plain language–part 1: governing principles and guidelines. https://www.iso.org/standard/78907.html.

[49] Wickham H, Averick M, Bryan J (2019). Welcome to the tidyverse. J Open Source Software.

[50] Bache SM, Wickham H, Henry L (March 20, 2022). magrittr: a forward-pipe operator for R. https://cran.r-project.org/web/packages/magrittr/index.html.

[51] Wickham H, Bryan J, Kalicinski M (July 6, 2023). readxl: read Excel files. https://cran.r-project.org/web/packages/readxl/index.html.

[52] Neuwirth E (April 3, 2022). RColorBrewer: ColorBrewer palettes. https://cran.r-project.org/web/packages/RColorBrewer/index.html.

[53] Arnold JB, Daroczi G, Werth B (Feb 10, 2024). ggthemes: extra themes, scales and geoms for ‘ggplot2’. https://cran.r-project.org/web/packages/ggthemes/index.html.

[54] South A (Oct 16, 2023). rworldmap: mapping global data. https://cran.r-project.org/web/packages/rworldmap/index.html.

[55] Wilkinson MD, Dumontier M, Aalbersberg IJJ (2016). The FAIR guiding principles for scientific data management and stewardship. Sci Data.

[56] UN Office for the High Commissioner for Human Rights (2018). A human rights based approach to data. Leaving no one behind in the 2030 agenda for sustainable development. https://www.ohchr.org/en/documents/tools-and-resources/human-rights-based-approach-data-leaving-no-one-behind-2030-agenda.

[57] Shaw RJ, Harron KL, Pescarini JM (2022). Biases arising from linked administrative data for epidemiological research: a conceptual framework from registration to analyses. Eur J Epidemiol.

[58] Observational Medical Outcomes Partnership Common data model. https://ohdsi.github.io/CommonDataModel/.

[59] Ghassemi M, Nsoesie EO (2022). In medicine, how do we machine learn anything real?. Patterns.

[60] Sjoding MW, Dickson RP, Iwashyna TJ, Gay SE, Valley TS (2020). Racial bias in pulse oximetry measurement. New Engl J Med.

[61] WHO (2021).

[62] Carroll SR, Garba I, Figueroa-Rodríguez OL (2020). The CARE principles for indigenous data governance. Data Sci J.

[63] First Nations Information Governance Centre (2020). The First Nations Principles of OCAP. https://fnigc.ca/ocap-training/.

[64] Prabhakaran V, Martin D (2020). Participatory machine learning using community-based system dynamics. Health Hum Rights.

[65] Ada Lovelace Institute (2022). Algorithmic impact assessment in healthcare. https://www.adalovelaceinstitute.org/project/algorithmic-impact-assessment-healthcare/.

[66] Information Commissioner's Office (2023). Data protection impact assessments (DPIAs). https://ico.org.uk/for-organisations/uk-gdpr-guidance-and-resources/accountability-and-governance/data-protection-impact-assessments-dpias/.

[67] Centre for Humanitarian Data, Office for Coordination of Humanitarian Affairs, UN An introduction to disclosure risk assessment. https://centre.humdata.org/learning-path/disclosure-risk-assessment-overview/.

[68] Liu X, Glocker B, McCradden MM, Ghassemi M, Denniston AK, Oakden-Rayner L (2022). The medical algorithmic audit. Lancet Digit Health.

[69] International Organization for Standardization ISO 31000:2018. Risk management—guidelines. https://www.iso.org/standard/65694.html.

[70] International Organization for Standardization IEC 31010:2019. Risk management—risk assessment techniques. https://www.iso.org/standard/72140.html.

[71] Riley RD, Collins GS (2023). Stability of clinical prediction models developed using statistical or machine learning methods. Biom J.

[72] Kauh TJ, Read JG, Scheitler AJ (2021). The critical role of racial/ethnic data disaggregation for health equity. Popul Res Policy Rev.

[73] Jacobs AZ, Wallach H (2021).

[74] Medicines and Healthcare products Regulatory Agency Software and AI as a Medical Device Change Programme. Roadmap. https://www.gov.uk/government/publications/software-and-ai-as-a-medical-device-change-programme/software-and-ai-as-a-medical-device-change-programme-roadmap.

[75] Liu X, Cruz Rivera S, Moher D, Calvert MJ, Denniston AK (2020). Reporting guidelines for clinical trial reports for interventions involving artificial intelligence: the CONSORT-AI extension. Nat Med.

[76] Vasey B, Nagendran M, Campbell B (2022). Reporting guideline for the early stage clinical evaluation of decision support systems driven by artificial intelligence: DECIDE-AI. BMJ.

[77] Collins GS, Moons KGM, Dhiman P (2024). TRIPOD+AI statement: updated guidance for reporting clinical prediction models that use regression or machine learning methods. BMJ.

