# Supplementary material for: Tackling algorithmic bias and promoting transparency in health datasets: the STANDING Together consensus recommendations
Source: Lancet Digit Health. 2024 Dec 18;7(1):e64–88. doi: 10.1016/S2589-7500(24)00224-3 (PMC11668905; doi:10.1016/S2589-7500(24)00224-3)
Supplement: Supplementary appendix [file mmc1.pdf]

THE LANCET
Digital Health

Supplementary appendix

This appendix formed part of the original submission and has been peer reviewed. We post it as supplied by the authors.

Supplement to: Alderman JE, Palmer J, Laws E, et al. Tackling algorithmic bias and promoting transparency in health datasets: the STANDING Together consensus recommendations. *Lancet Digit Health* 2024; published online Dec 18. https://doi.org/10.1016/S2589-7500(24)00224-3.

STANDING Together

Tackling algorithmic bias and promoting transparency in health datasets: the STANDING Together consensus recommendations.

Web appendix:

Appendix (page 2)
List of attributes extracted from a prior systematic review, and added to by respondents to the delphi surveys.

Supplementary Table (pages 2 to 5)
Information about participants for each round of the Delphi survey.

Supplementary Figure 1 (page 6)
Delphi voting interface.

Supplementary Figure 2 (page 7)
Breakdown of participants completing each round of the Delphi survey by role.

Supplementary Figure 3 (page 8)
Reach of the STANDING Together project disaggregated by type of involvement.

Supplementary File 1 (pages 9 to 43)
Evolution of recommendation items with changes tracked across Delphi rounds.

Supplementary File 2 (pages 44 to 85)
Question set for the first online Delphi survey round.

Supplementary File 3 (pages 86 to 148)
Question set for the second online Delphi survey round.

Supplementary File 4 (pages 149 to 154)
Question set for the third online Delphi survey round.

Appendix

List of attributes extracted from a prior systematic review, and added to by respondents to the delphi surveys. Any of these may be 'relevant' depending on the context.

(Systematic review citation: Arora A, Alderman JE, Palmer J, Ganapathi S, Laws E, McCradden MD, et al. The value of standards for health datasets in artificial intelligence-based applications. Nat Med [Internet]. 2023 Nov;29(11):2929–38. Available from: http://dx.doi.org/10.1038/s41591-023-02608-w)

Age, gender, sex, race, ethnicity, socioeconomic status, sexual orientation, disability, gender reassignment, marriage and civil partnership, pregnancy and maternity, religion or belief, nationality, ancestry, occupation, language, caste, creed, veteran status, education, rural or urban residential status, neurodiversity, displaced individuals or refugee status.

Supplementary Table

Information about participants for each round of the Delphi survey.

*Participants were able to select multiple professional backgrounds and/or to select 'other' and self identify; based on their choice they were allocated a 'role' to enable grouping of their survey responses with those from other participants. Participants who had selected 'Member of the public' were allocated this as their role only if this was the only option they had chosen. Where participants had selected multiple professional backgrounds, a single role was allocated using the following logic: (IF one of the selected professional backgrounds is 'Policy, regulation, law', allocate this as the role; ELSE (IF not already allocated a role AND one of the selected professional backgrounds is 'Computer scientist / data scientist', allocate this as the role; ELSE (IF not already allocated a role AND one of the selected professional backgrounds is 'Healthcare professional', allocate this as the role; ELSE allocate the role as 'Other'))). Participants for round 1 were not asked whether they were a dataset curator or whether they were involved in creating AI / machine learning models for healthcare. Participants for round 3 were not asked to provide demographic information relating to personal identity. *Participants were asked to voluntarily self-report ethnicity using UK Office for National Statistics (ONS) categories, though recognising that these are not inclusive for all geographies and communities, they were able to self-identify and submit a free-text response. The ONS categories are listed at the following link: https://www.ons.gov.uk/methodology/classificationsandstandards/measuringequality/ethnicgroupnationalidentityandreligion#different-aspects-of-ethnicity*

**Participant demographic data**	**Delphi voting rounds**		
	**Round 1 (n = 194)**	**Round 2 (n = 106)**	**Round 3 (n = 71)**
**Age range**			
18-30	21 (10.8%)	9 (8.5%)	
30-60	146 (75.3%)	85 (80.2%)	
60+	15 (7.7%)	7 (6.6%)	
Prefer not to answer	3 (1.5%)	3 (2.8%)	
Not answered	9 (4.6%)	2 (1.9%)	
**"What is your sex (as assigned at birth)?"**			
Female	70 (36.1%)	45 (42.5%)	
Male	100 (51.5%)	53 (50.0%)	
Other (including intersex)	1 (0.5%)	0 (0%)	
Prefer not to answer	5 (2.6%)	2 (1.9%)	
Not answered	18 (9.3%)	6 (5.7	

Continues on next page

**"Is your gender identity the same as the sex you were assigned at birth?"**			
Yes	170 (87.6%)	95 (89.6%)	
No	2 (1.0%)	1 (0.9%)	
Prefer not to answer	6 (3.1%)	1 (0.9%)	
Not answered	16 (8.2%)	9 (8.5%)	
**"How do you describe your sexual orientation?"**			
Homosexual	15 (7.7%)	4 (3.8%)	
Bisexual	10 (5.2%)	5 (4.7%)	
Queer, pan, asexual, or prefer to self define	5 (2.6%)	3 (2.8%)	
Heterosexual	134 (69.1%)	72 (67.9%)	
Prefer not to answer	13 (6.7%)	11 (10.4%)	
Not answered	17 (8.8%)	11 (10.4%)	
**"What is your ethnicity?"***			
African	9 (4.6%)	5 (4.7%)	
Arab	2 (1.0%)	2 (1.9%)	
Bangladeshi	2 (1.0%)	0 (0%)	
Chinese	8 (4.1%)	3 (2.8%)	
English / Welsh / Scottish / Northern Irish / British	55 (26.4%)	38 (35.8%)	
Indian	16 (8.2%)	8 (7.5%)	
Irish	4 (2.1%)	2 (1.9%)	
Pakistani	4 (2.1%)	1 (0.9%)	
White and Asian	3 (1.5%)	2 (1.9%)	
White and Black African	2 (1.0%)	0 (0%)	
White and Black Caribbean	1 (0.5%)	0 (0%)	
Any other Asian background	12 (6.2%)	6 (5.7%)	
Any other Black / African / Caribbean background	1 (0.5%)	2 (1.9%)	
Any other Mixed / Multiple ethnic background	10 (5.2%)	2 (1.9%)	
Any other White background	38 (19.6%)	25 (23.6%)	
Any other ethnic group	3 (1.5%)	1 (0.9%)	
I'd prefer not to answer this question	12 (6.2%)	2 (1.9%)	
Not answered	12 (6.2%)	7 (6.6%)	
**"Which of the following statements best describe you?"**			
I'm a computer scientist / data scientist	45 (23.2%)	28 (26.4%)	14 (19.7%)
I'm an academic researcher	87 (44.8%)	43 (40.6%)	31 (43.7%)
I'm a healthcare professional	70 (36.1%)	42 (39.6%)	24 (33.8%)
I'm a member of the public	18 (9.3%)	9 (8.5%)	10 (14.1%)
I work in policy, regulation, law, politics or related fields	31 (16.0%	17 (16.0%)	10 (14.1%)
I work in social science	6 (3.1%)	6 (5.7%)	4 (5.6%)
Other	27 (13.9%)	10 (9.4%)	9 (12.7%)

Continues on next page

**Assigned roles for delphi survey analysis**<br>Member of the public<br>Healthcare professional<br>Computer science / data science<br>Policy, regulation, law<br>Other	 12 (6.2%)<br>59 (30.4%)<br>40 (20.6%)<br>31 (16.0%)<br>52 (25.8%)	 8 (7.5%)<br>35 (33.0%)<br>25 (23.6%)<br>17 (16.0%)<br>21 (19.8%)	 8 (11.3%)<br>19 (26.8%)<br>11 (15.5%)<br>10 (14.1%)<br>23 (32.4%)
**“Are you a dataset curator?”**<br>Yes<br>No		 49 (46.2%)<br>57 (53.8%)	 34 (47.9%)<br>37 (52.1%)
**“Are you involved in creating AI/ML models for healthcare?”**<br>Yes<br>No		 61 (57.5%)<br>45 (42.5%)	 39 (54.9%)<br>32 (45.1%)

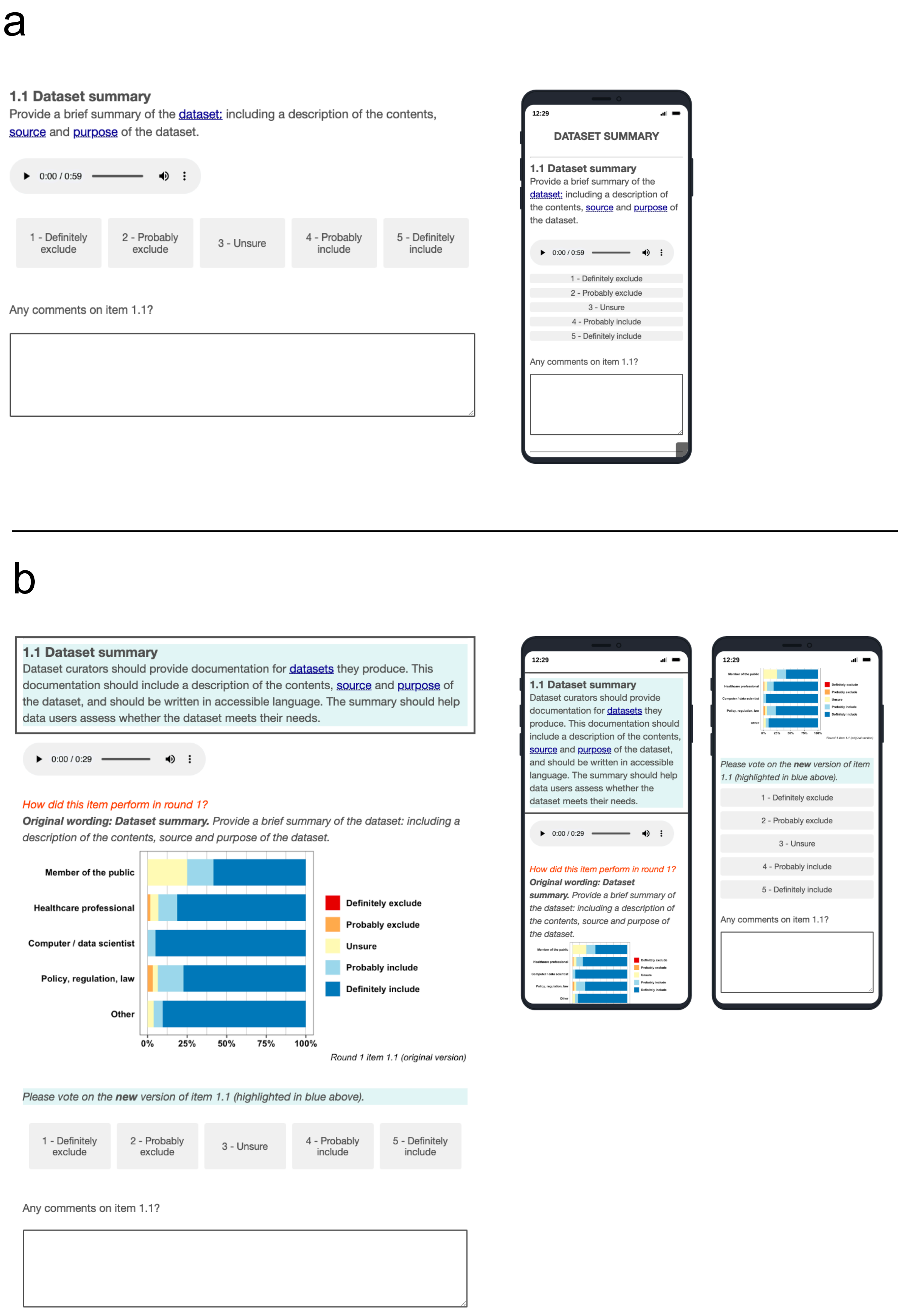

Supplementary Figure 1 *- Interface provided for participants undertaking the online Delphi survey voting rounds. Rounds 1 and 3 shared the same interface. For round 2, participants were provided with a graph showing the performance of the item in question in the previous round of voting.*

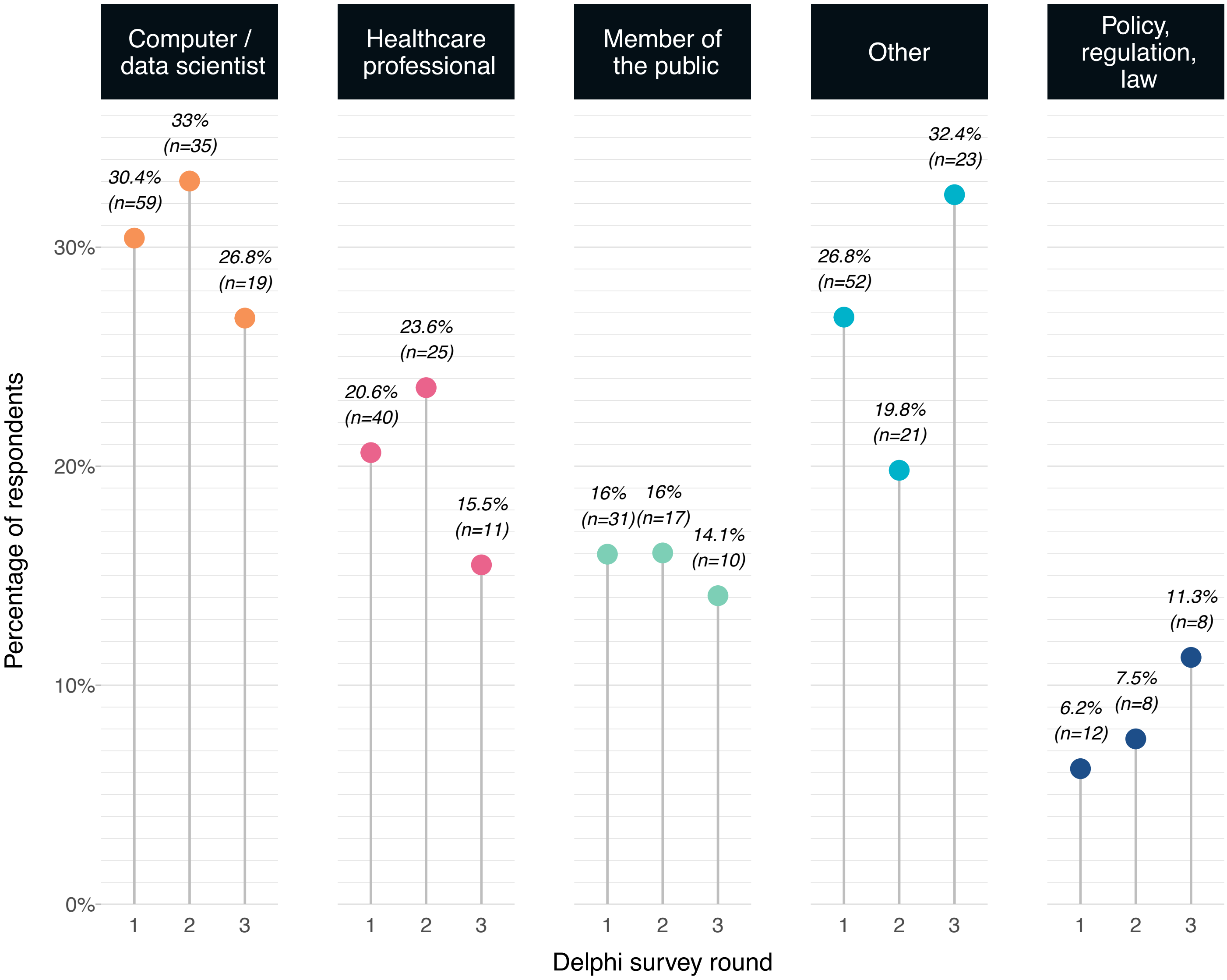

***Supplementary Figure 2** - Breakdown of participants completing each round of the Delphi survey according to their role. Labels above each data point show the proportion of each role as a percentage of all participants for each respective Delphi survey round. The total numbers of participants completing rounds one, two and three of the Delphi survey were 194, 106, and 71 respectively. Note – because percentages have been rounded they do not total exactly 100%.*

A - Delphi participants

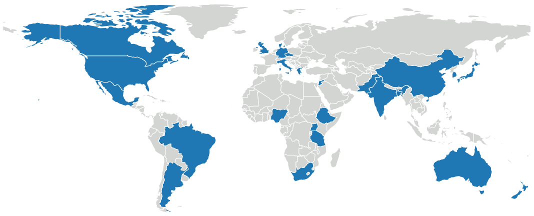

B - Patient and public participation

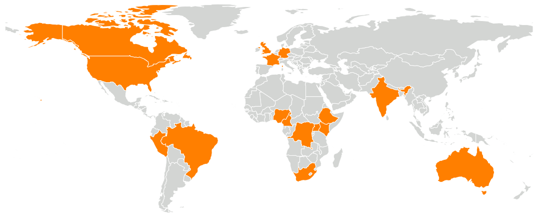

C - Consensus meeting attendees and working group

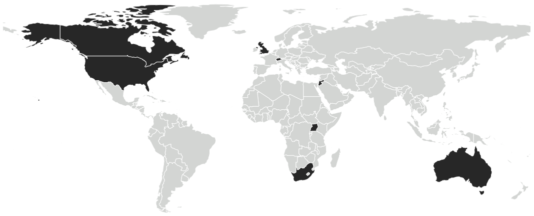

D - Interview study

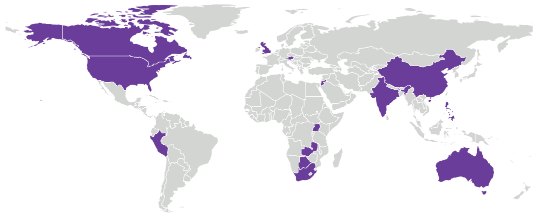

E - Overall reach of the STANDING Together initiative

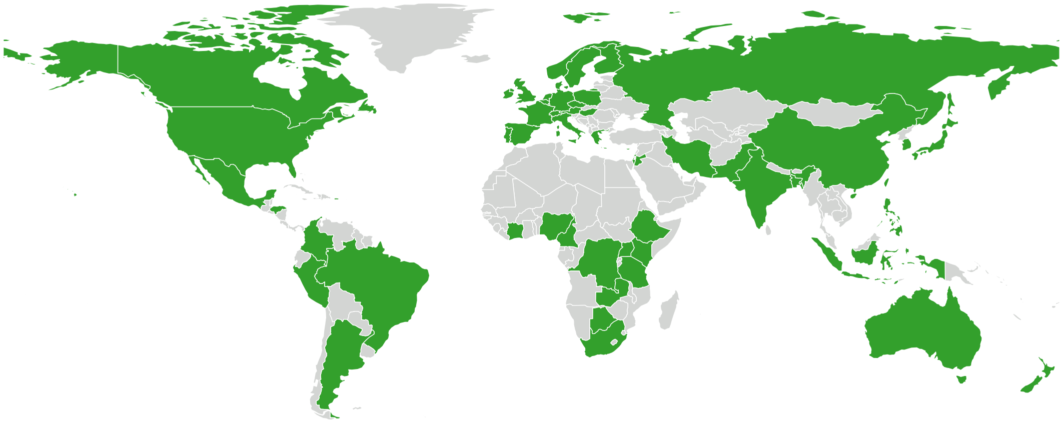

Supplementary Figure 3 - *Reach of the STANDING Together project disaggregated by type of involvement:*
A *- Nationality of participants in the Delphi study (25 countries).*
B *- Geographical location of members of the patient and public involvement and engagement group and the international advisory group (16 countries).*
C *- Geographical location of attendees at the consensus meeting and members of the working group (9 countries).*
D *- Participants in the interview study (14 countries).*
E *- Overall reach of the STANDING Together project, including A-D and those who accessed the draft recommendations from the project website during the public consultation (58 countries).*

Supplementary File tracking recommendation item performance across delphi rounds.

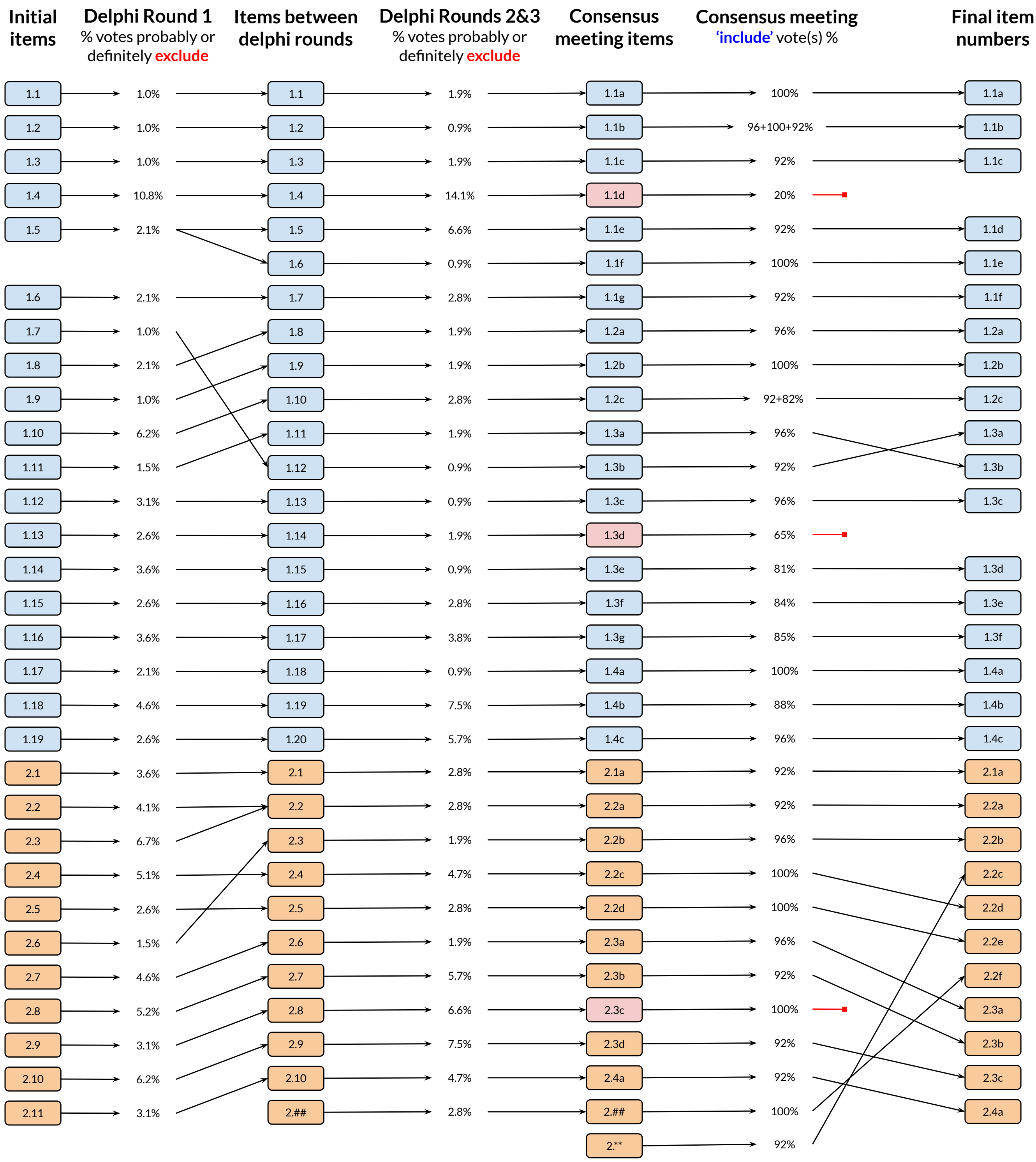

Summary diagram showing item performance across delphi voting rounds. Across each round, items were split apart, merged, added and removed. Specific details are given on subsequent pages. Three items was excluded during the consensus meeting – two because they failed to meet the threshold for inclusion (70% of eligible participants voting to include), and one because the participants requested that it instead be merged into the explanatory text of other items (see page 35 for further details of this last item).

1.1a - Dataset summary

Dataset documentation should include a summary of the dataset written in Plain Language. This summary should state the Data Origin and its purpose, and give a short description of the content to help data users assess whether the dataset meets their needs.

DELPHI ROUND 1 WORDING	DELPHI ROUND 2 WORDING	WORDING PRESENTED TO CONSENSUS MEETING
**1.1 Dataset summary**<br>Provide a brief summary of the dataset: including a description of the contents, source and purpose of the dataset<br>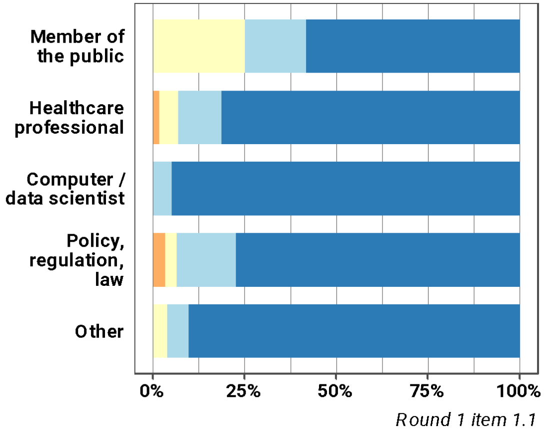<br>1.0% vote to probably/definitely exclude<br>94.3% vote to probably/definitely include	**1.1 Dataset summary**<br>Dataset curators should provide documentation for datasets they produce. This documentation should include a description of the contents, source and purpose of the dataset, and should be written in accessible language. The summary should help data users assess whether the dataset meets their needs.<br>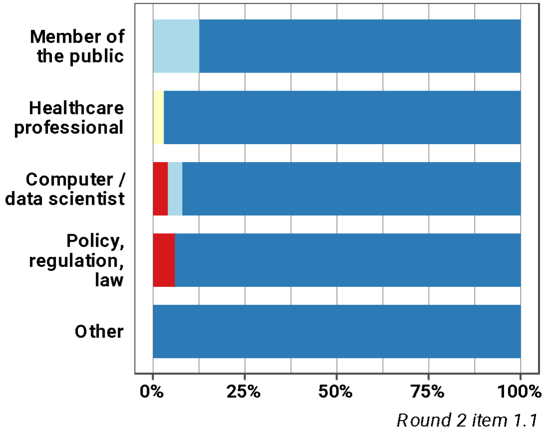<br>1.9% vote to probably/definitely exclude<br>97.2% vote to probably/definitely include	**1.1a Dataset summary**<br>Dataset curators should provide documentation for datasets they produce. This documentation should include a description of the contents, source and purpose of the dataset, and should be written in accessible language. The summary should help data users assess whether the dataset meets their needs.<br><br>**ACTIONS AT CONSENSUS MEETING**<br>- Minor reword<br>- 100% voted to include<br>- Item included in final recommendations<br><br>**AMENDMENTS FOR FINAL ITEM**<br>- Minor reword for consistency with other items, and to clarify that the summary should 'give a short description of the content'.

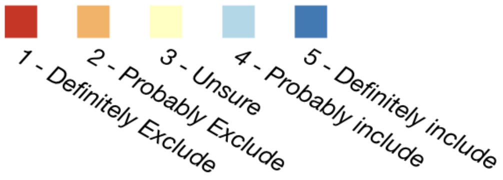

1.1b - Dataset identity and access

Dataset documentation should:

- State the dataset's identity, including a persistent identifier and information regarding date(s) of release.
- Provide information on how the data can be accessed, including permitted use, licensing arrangements and details of the data custodian(s).
- Describe adherence to principles for data use and access.

DELPHI ROUND 1 WORDING	DELPHI ROUND 2 WORDING	CONSENSUS MEETING WORDING & FINAL ITEM
**1.2 Dataset identity and access**<br>Provide essential information regarding accessibility, date of release, version, dataset size, licensing and ownership.<br>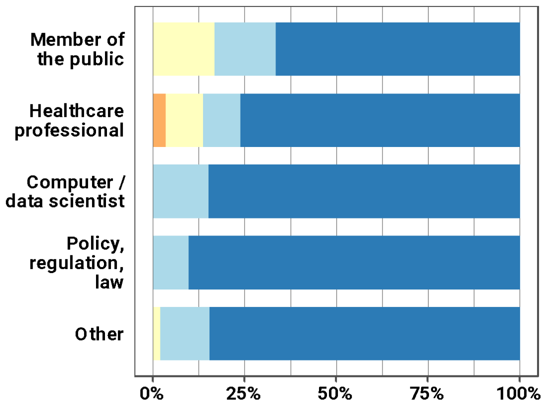*Round 1 item 1.2*<br>1.0% vote to probably/definitely exclude<br>94.3% vote to probably/definitely include	**1.2 Dataset identity and access**<br>Dataset documentation should include: dataset name, accessibility, date of release, version, licensing arrangements, and details of the data custodian(s). Where possible this documentation should adhere to FAIR principles.<br>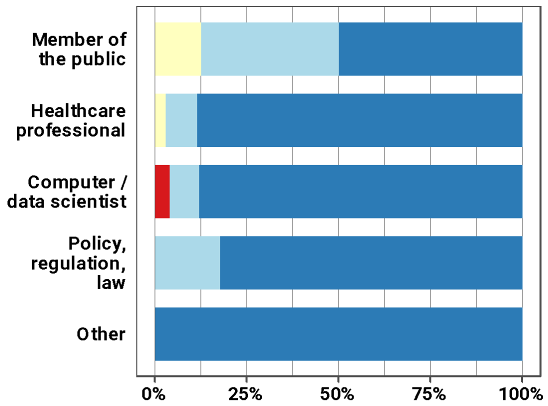*Round 2 item 1.2*<br>0.9% vote to probably/definitely exclude<br>97.2% vote to probably/definitely include	**1.1b Dataset identity and access**<br>Dataset documentation should include: dataset name, accessibility, date of release, version, licensing arrangements, and details of the data custodian(s). Where possible this documentation should adhere to FAIR principles.<br><br>**ACTIONS AT CONSENSUS MEETING**<br>- First vote: 89% include, 11% abstain<br>- Substantial reword<br>- Added reference to data sovereignty<br>- Second vote held in three parts to ascertain consensus for each item subpoint: i) 96% include, 4% abstain; ii) 100% include; iii) 92% include, 4% exclude, 4% abstain<br>- Item included in final recommendations<br>- Attendees requested that research team refine language after meeting<br><br>**AMENDMENTS FOLLOWING CONSENSUS MEETING**<br>- Minor language refinement<br>- Moved reference to data sovereignty to item 1.4a

1.1c - Reasons behind dataset creation and its purpose(s)
Dataset documentation should include the reasons why this dataset was created, including any intended benefit(s), any purposes for which dataset use should be avoided, who created the dataset (including any competing interests), and who funded it.

1.3 Motivations for dataset creation and intended purpose(s)
Describe the motivations behind the creation of this dataset, including: the purpose for dataset creation, the intended benefit, any purposes for which dataset use should be avoided, who created the dataset and who funded it.

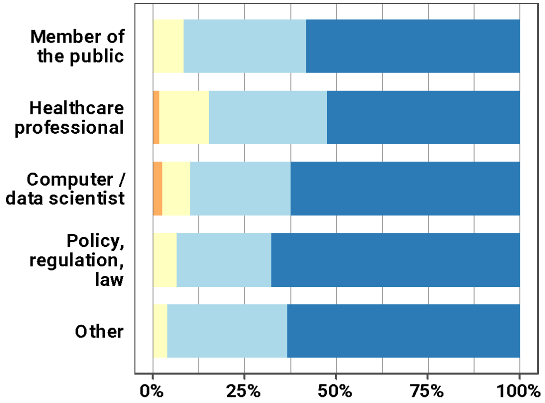

Round 1 item 1.3

1.0% vote to probably/definitely exclude
90.7% vote to probably/definitely include

1.3 Motivations for dataset creation and intended purpose(s)
Dataset documentation should include the reasons why this dataset was created, including any intended benefit(s), any purposes for which dataset use should be avoided, who created the dataset, and who funded it.

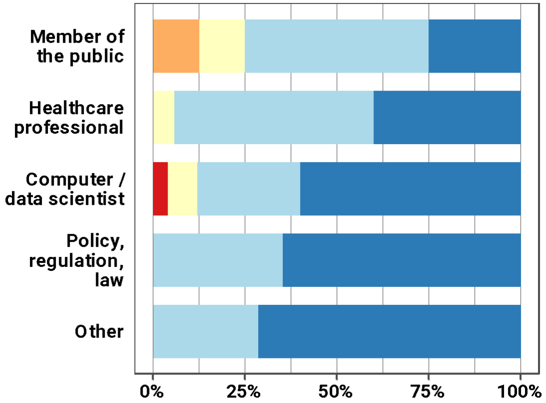

Round 2 item 1.3

1.9% vote to probably/definitely exclude
93.4% vote to probably/definitely include

1.1c Motivations for dataset creation and intended purpose(s)
Dataset documentation should include the reasons why this dataset was created, including any intended benefit(s), any purposes for which dataset use should be avoided, who created the dataset, and who funded it.

ACTIONS AT CONSENSUS MEETING
- 92% voted to include, 8% abstain
- Item included in final recommendations

AMENDMENTS FOLLOWING CONSENSUS MEETING
- Added 'including any competing interests'.

1.1d - Data Origin

Dataset documentation should describe the Data Origin, why it was selected, and what individuals were told would happen to their data.

1.1e - Data sampling and aggregation from multiple sources

Dataset documentation should describe how data were sampled from the Data Origin and any Source Dataset(s), including an explanation of sampling strategies, their rationale, and potential impact on the composition of the dataset. If the dataset has been compiled from multiple Data Origins and/or Source Datasets, dataset documentation should describe how each was selected, and how decisions were made during data aggregation, particularly in the case of grouping populations and modification of demographic coding.

**1.5 Relation to the original data source, including sampling strategy**<br>*(split into two items for round 2)*<br>Describe the original source of data and the data sampled, including an explanation of sampling strategies and their rationale. Describe the reason for generating the original data (e.g., patient records to provide clinical care, clinical trial, biobank) and what individuals were expecting to happen to their data (e.g., administrative action, participant in a research study). If the dataset has been compiled from multiple data sources, describe how datasets were selected, and how decisions were made during data aggregation, particularly in the case of grouping populations and modification of demographic coding.<br>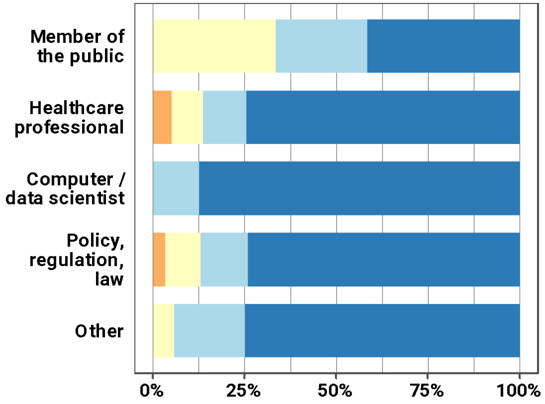<br>*Round 1 item 1.5*	**1.5 Origin and purpose of source data**<br>Dataset documentation should describe the original source of data, including the reason for generating the original data (e.g., patient records to provide clinical care, clinical trial, biobank) and what individuals were expecting to happen to their data (e.g., administrative action, participant in a research study).<br>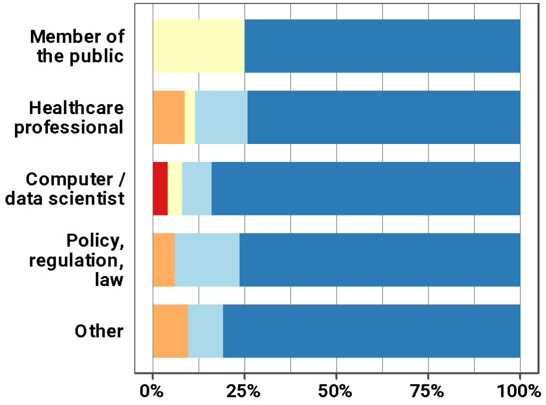<br>*Round 2 item 1.5*<br>6.6% vote to probably/definitely exclude<br>89.6% vote to probably/definitely include	**1.1e Origin and purpose of source data**<br>Dataset documentation should describe the original source of data (e.g., patient records to provide clinical care, clinical trial, biobank) and what individuals were expecting to happen to their data (e.g., administrative action, participant in a research study).<br><br>**<u>ACTIONS AT CONSENSUS MEETING</u>**<br>- Minor reword<br>- Vote: 92% include, 4% exclude, 4% abstain<br>- Item included in final recommendations<br><br>**<u>AMENDMENTS FOLLOWING CONSENSUS MEETING</u>**<br>- Introduced 'Data Origin' and 'Dataset Source' throughout recommendations to clarify the difference between the clinical environment in which data are first generated, and any downstream repositories from which data may be sampled<br>- Moved examples to explanatory text for consistency with other items<br>- Added 'why it was selected'.
	**1.6 Data sampling, and aggregation from multiple sources**<br>Dataset documentation should describe how data were sampled from the original data source, including an explanation of sampling strategies and their rationale. If the dataset has been compiled from	**1.1f Data sampling, and aggregation from multiple sources**<br>Dataset documentation should describe how data were sampled from the original data source, including an explanation of sampling strategies and their rationale. If the dataset has been compiled from

2.1% vote to probably/definitely exclude<br>90.2% vote to probably/definitely include	multiple data sources, dataset documentation should describe how datasets were selected, and how decisions were made during data aggregation, particularly in the case of grouping populations and modification of demographic coding.<br>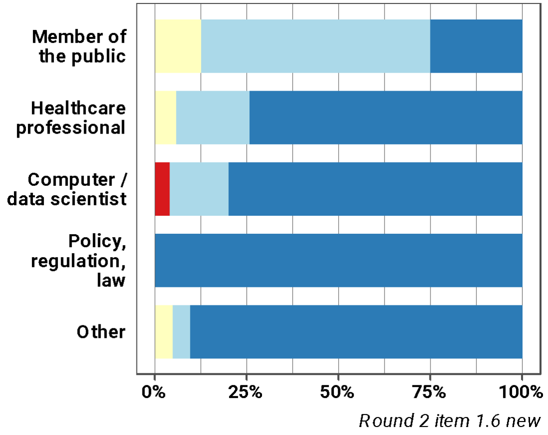<br>0.9% vote to probably/definitely exclude<br>95.3% vote to probably/definitely include	multiple data sources, dataset documentation should describe how datasets were selected, and how decisions were made during data aggregation, particularly in the case of grouping populations and modification of demographic coding.<br><br>**ACTIONS AT CONSENSUS MEETING:**<br>- Minor reword<br>- Vote: 100% include<br>- Item included in final recommendations<br><br>**AMENDMENTS FOLLOWING CONSENSUS MEETING**<br>- Introduced 'Data Origin' and 'Dataset Source' throughout recommendations to clarify the difference between the clinical environment in which data are first generated, and any downstream repositories from which data may be sampled<br>- Minor rewording for clarity

1.1f - Data shifts over time

For longitudinal datasets or datasets with multiple versions, dataset documentation should describe any known or expected changes over time relating to the population, medical practice, or how data were collected (including devices, sensors and software used), which may contribute to Data Shifts over time.

1.6 Data shifts

For longitudinal datasets or datasets with versions, describe any changes over time relating to the population, medical practice, or how data were collected, which may contribute to data shifts.

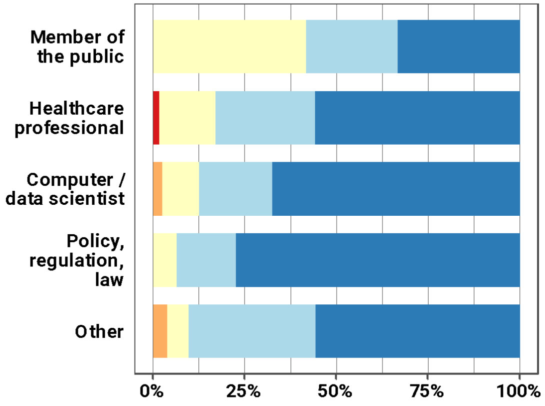

Round 1 item 1.6

2.1% vote to probably/definitely exclude
86.1% vote to probably/definitely include

1.7 Data shifts

(numbering out of sync with round 1 because of splitting of 'Round 1 item 1.5' into two items: 'Round 2 item 1.5' and 'Round 2 item 1.6')

For longitudinal datasets or datasets with versions, dataset documentation should describe any known or suspected changes over time relating to the population, medical practice, or how data were collected, which may contribute to data shifts.

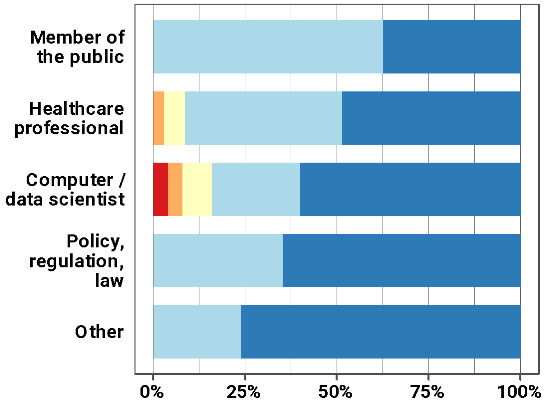

Round 2 item 1.7

2.8% vote to probably/definitely exclude
93.4% vote to probably/definitely include

1.1g Data shifts

For longitudinal datasets or datasets with versions, dataset documentation should describe any known or suspected changes over time relating to the population, medical practice, or how data were collected, which may contribute to data shifts.

ACTIONS AT CONSENSUS MEETING

- Minor reword
- Vote: 92% include, 8% exclude
- Item included in final recommendations

AMENDMENTS FOLLOWING CONSENSUS MEETING

- Minor reword of title to append 'over time'
- Added 'including devices, sensors and software used'

1.2a - Composition of groups within the dataset

Dataset documentation should:

- Include a summary of the groups present in the dataset. The choice of which groups to describe, and the means of categorisation should be explained.
- Highlight any known missing groups within the dataset and any reason(s) for their missingness.

<table>
<tr>
<td>

1.8 Composition of populations

Summarise the relevant populations present in the dataset. Defining 'relevant populations' should be contextualised according to this dataset's specific use cases and contexts, where possible. Certain attributes (including age, gender identity, sex, race, ethnicity, socioeconomic status) should always be documented, due to known associations with health outcomes and interactions with wider social factors. If data on these particular attributes are missing this should be stated. Highlight known missing population groups within the dataset and the reason for their missingness.

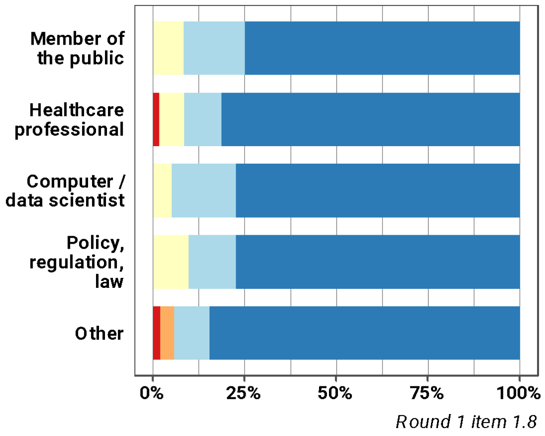

2.1% vote to probably/definitely exclude
92.8% vote to probably/definitely include

</td>
<td>

1.8 Composition of populations

Dataset documentation should:

- Summarise the populations present in the dataset. The choice of which populations to describe, and the choice of grouping/categorisation, should be explained.
- Highlight known missing groups within the dataset and any reason(s) for their missingness.

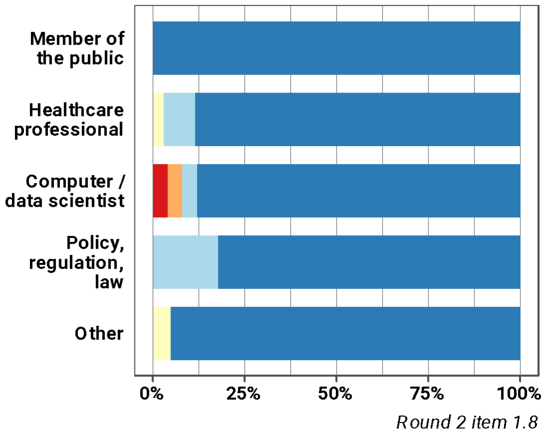

1.9% vote to probably/definitely exclude
96.2% vote to probably/definitely include

</td>
<td>

1.2a Composition of populations

Dataset documentation should:

- Include a summary of the populations present in the dataset. The choice of which populations to describe, and the choice of grouping/ categorisation, should be explained.
- Highlight any known missing groups within the dataset and any reason(s) for their missingness.

<u>**ACTIONS AT CONSENSUS MEETING**</u>

- Vote: 96% include, 4% abstain
- Item included in final recommendations

<u>**AMENDMENTS FOLLOWING CONSENSUS MEETING**</u>

- Unified language in the item – both ‘populations’ and ‘groups’ used originally, simplified to refer only to ‘groups’

</td>
</tr>
</table>

1.2b - Recording of Individuals' Attributes

Dataset documentation should:

- Describe how and why individuals' Attributes are provided in the dataset , and whether this information is available at the individual or aggregate level.
- Explain whether Attributes have been coded, condensed, derived or modified, stating how and why this was done.
- Highlight the proportion of Attributes recorded as 'unknown' or 'other' and 'prefer not to say', and if possible explain the reasons why.

**1.9 Recording of attributes of individuals**	**1.9 Recording of attributes of individuals**	**1.2b Recording of attributes of individuals**
Describe how attributes were collected (self-reported, imputed, linked through other datasets), whether this information is available at the individual or aggregate level, and whether data missingness is different across groups. Explain whether attributes have been coded, condensed or modified, stating how and why this was done.<br>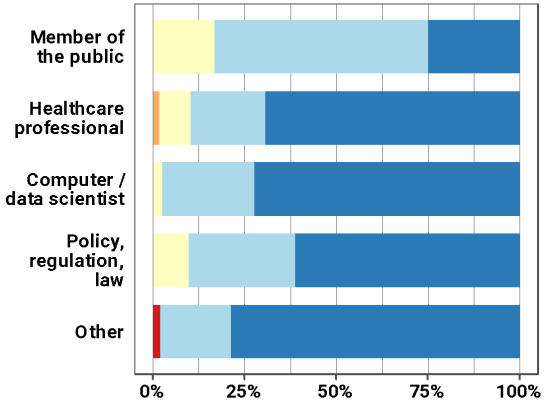<br><br>*Round 1 item 1.9*<br>1.0% vote to probably/definitely exclude<br>93.3% vote to probably/definitely include	Dataset documentation should:<br>- Describe how and why attributes are provided in the dataset (self-reported by participants, imputed, linked from other datasets), and whether this information is available at the individual or aggregate level.<br>- Explain whether attributes have been coded, condensed or modified, stating how and why this was done.<br>- Highlight the proportion of attributes recorded as 'unknown' or 'other', and if possible explain the reasons why<br>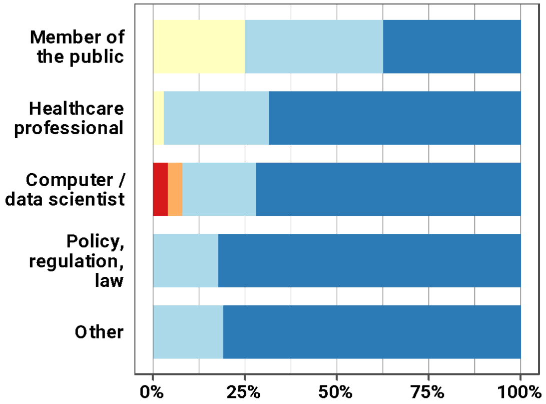<br><br>*Round 2 item 1.9*<br>1.9% vote to probably/definitely exclude<br>95.3% vote to probably/definitely include	Dataset documentation should:<br>▪ Describe how and why attributes are provided in the dataset (self- reported by participants, imputed, linked from other datasets), and whether this information is available at the individual or aggregate level.<br>▪ Explain whether attributes have been coded, condensed or modified, stating how and why this was done.<br>▪ Highlight the proportion of attributes recorded as 'unknown' or 'other', and if possible explain the reasons why.<br><br>**<u>ACTIONS AT CONSENSUS MEETING</u>**<br>- Minor reword<br>- Addition of 'prefer not to say' to third bullet<br>- Vote: 100% include<br>- Item included in final recommendations<br><br>**<u>AMENDMENTS FOLLOWING CONSENSUS MEETING</u>**<br>- Simplified title of item<br>- Moved examples to explanatory text for consistency with other items<br>- Added reference to 'derived' attributes

1.2c - Groups at risk of disparate health outcomes

Dataset documentation should:

- Include data (when available) on Relevant Attributes relating to individuals included within the dataset. If including these data may place individuals at risk of identification or endanger them, these data should instead be provided at aggregate level. If data on Relevant Attributes are missing, reasons for this should be stated.
- Highlight the presence of groups who are at risk of disparate health outcomes caused by structural or societal factors in this dataset, with consideration of both risk factors that are universal, and those that are specific to the site of data collection.

1.10 Groups experiencing vulnerabilities

Highlight the presence of any vulnerable population groups in this dataset, with consideration of both vulnerabilities that are universal (e.g., children, people with severe disabilities, displaced persons) and those that are specific to the site of data collection (e.g, marginalised religious or caste groups, sexual orientation and gender identity groups).

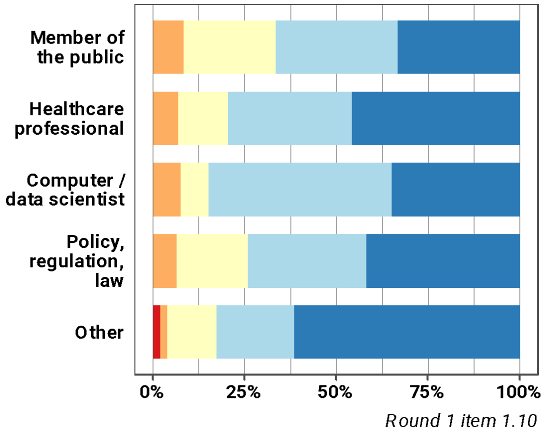

6.2% vote to probably/definitely exclude
79.9% vote to probably/definitely include

1.10 Groups at particular risk of harm.

Dataset documentation should:

- Always include data (when available) on certain attributes (including age, gender identity, sex, race, ethnicity, socioeconomic status, and sexual orientation) should always be documented, due to known associations with health outcomes and interactions with wider social factors. If including these data may place individuals at risk of identification or endanger them, these data should instead be provided at aggregate level for the whole dataset. If data on these particular attributes are missing, reasons for this should be stated.
- Highlight the presence of any vulnerable population groups in this dataset, with consideration of both vulnerabilities that are universal (e.g., children, people with severe disabilities, displaced persons) and those that are specific to the site of data collection (e.g, marginalised religious or caste groups).

1.2c Groups at particular risk of harm

Dataset documentation should:

- Always include data (when available) on certain attributes (including age, gender identity, sex, race, ethnicity, socioeconomic status, and sexual orientation), due to known associations with health outcomes and interactions with wider social factors. If including these data may place individuals at risk of identification or endanger them, these data should instead be provided at aggregate level for the whole dataset. If data on these particular attributes are missing, reasons for this should be stated.
- Highlight the presence of any vulnerable population groups in this dataset, with consideration of both vulnerabilities that are universal (e.g., children, people with severe disabilities, displaced persons) and those that are specific to the site of data collection (e.g, marginalised religious or caste groups).

<u>**ACTIONS AT CONSENSUS MEETING**</u>

- Extended debate about whether specific attributes should be listed in the item text. Indicative vote held on this question – 62% include in item text, 30% exclude from item text, 8% abstain.
- Subsequent debate and vote on whether specific attributes should be instead listed in

	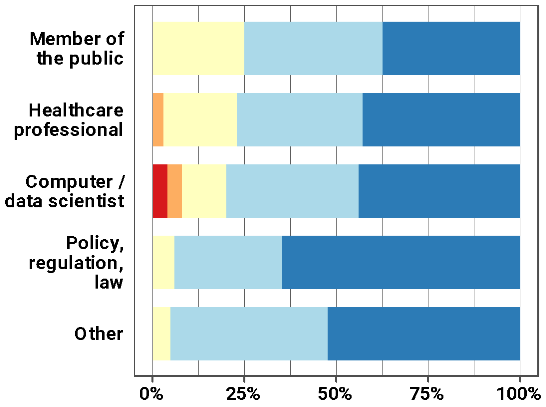<br><br>*Round 2 item 1.10*<br>2.8% vote to probably/definitely exclude<br>84.0% vote to probably/definitely include	explanatory text – 85% include, 11% exclude, 4% abstain.<br>- Rewording of proposed new item without specific attributes listed, removing request to ‘always’ include such data, and removing term ‘vulnerable’, replacing with ‘at risk’<br>- Separate votes on each bullet point:<br>- First bullet: 92% include 8% exclude<br>- Second bullet: 77% include 11.5% exclude 11.5% abstain; quorum not met so re-discussed item. Added reference to ‘structural’ factors causing risk<br>- Second bullet revote: 81% include, 11% exclude, 8% abstain<br>- Item included in final recommendations<br><br>**<u>AMENDMENTS FOLLOWING CONSENSUS MEETING</u>**<br>- Introduced new defined term ‘Relevant Attributes’ to replace ‘certain Attributes’ for consistency with other items<br>- Moved ‘due to known associations with health outcomes and interactions with wider social factors’ to the definition of Relevant Attributes<br>- Reframed item title as ‘disparate health outcomes’ from ‘harm’ for consistency with other items<br>- Clarified that Relevant Attributes relate to Individuals<br>- Minor reword of second bullet point for clarity, and added reference to ‘societal’ factors<br>- Moved examples to explanatory text for consistency with other items

1.3a - Limitations of the dataset
Dataset documentation should identify known or expected sources of bias, error or other factors that affect the dataset as a whole, which may impact its generalisability or applicability.

<table>
<tr>
<td>

1.7 Limitations of the dataset
Identify known or suspected sources of bias, error or other factors that affect the dataset as a whole, which may impact its generalisability or applicability for other use.

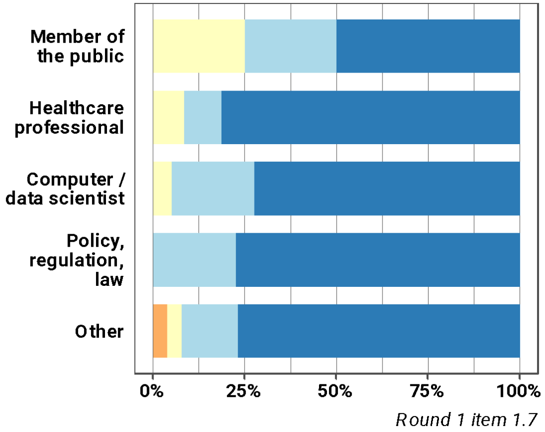

1.0% vote to probably/definitely exclude
92.8% vote to probably/definitely include

</td>
<td>

1.12 Limitations of the dataset
(re-ordered following delphi round 1)
Dataset documentation should identify known or suspected sources of bias, error or other factors that affect the dataset as a whole, which may impact its generalisability or applicability for other use.

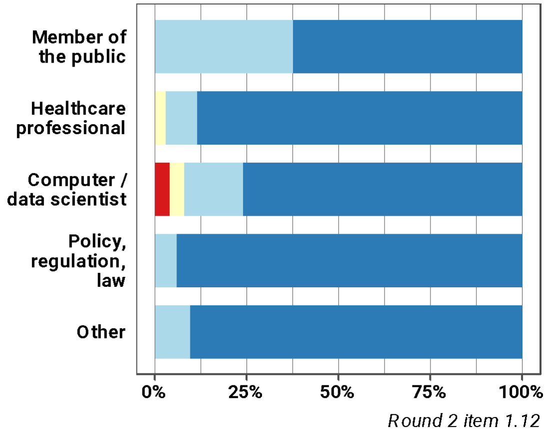

0.9% vote to probably/definitely exclude
97.2% vote to probably/definitely include

</td>
<td>

1.3b Limitations of the dataset
Dataset documentation should identify known or suspected sources of bias, error or other factors that affect the dataset as a whole, which may impact its generalisability or applicability for other use.

ACTIONS AT CONSENSUS MEETING:
- Minor reword
- Vote: 92% include, 4% exclude, 4% abstain
- Item included in final recommendations

AMENDMENTS FOLLOWING CONSENSUS MEETING:
- N/a

</td>
</tr>
</table>

1.3b - Modifications made to the data

Dataset documentation should describe whether any data items were modified from the original source, or if any of the data is synthetic, providing the rationale for doing so and any methods used.

1.11 Modifications made to the data

Describe data items which were modified from the raw source and provide the rationale for doing so and the method used. For example, for anonymisation, to correct for imbalance, to correct errors or biases, mapping to existing data standards (e.g. OMOP, FHIR, DICOM).

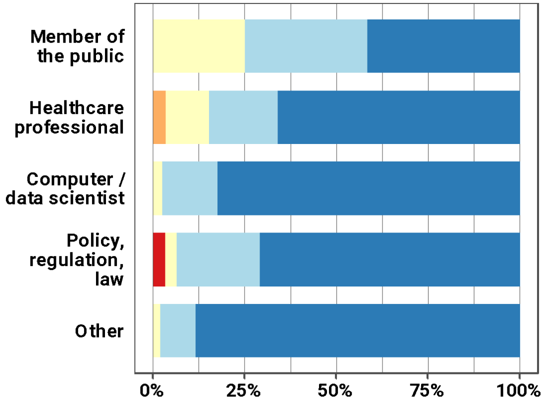

Round 1 item 1.11

1.5% vote to probably/definitely exclude
91.8% vote to probably/definitely include

1.11 Modifications made to the data

Dataset documentation should describe whether any data items were modified from the original source, providing the rationale for doing so and any methods used. For example; for anonymisation, to correct for imbalance, to correct errors or biases, or to enable mapping to existing data standards.

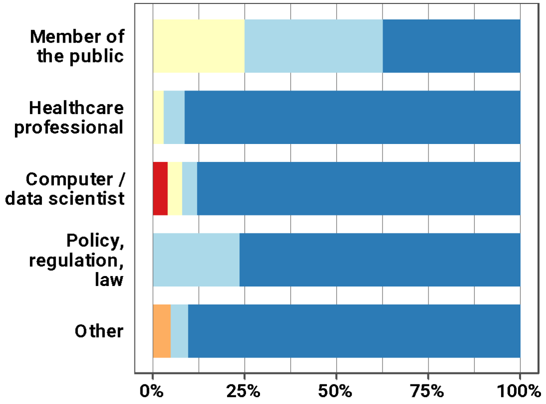

Round 2 item 1.11

1.9% vote to probably/definitely exclude
94.3% vote to probably/definitely include

1.3a Modifications made to the data

Dataset documentation should describe whether any data items were modified from the original source, providing the rationale for doing so and any methods used. For example; for anonymisation, to correct for imbalance, to correct errors or biases, or to enable mapping to existing data standards.

ACTIONS AT CONSENSUS MEETING

- Added reference to synthetic data
- Removed example, and noted that this should instead appear in the explanatory text
- Vote: 96% include, 4% exclude
- Item included in final recommendations

AMENDMENTS FOLLOWING CONSENSUS MEETING:

- N/A

1.3c - Missing data

Dataset documentation should describe the proportion, nature and causes of missing data (if known), particularly if there are systematic differences across groups within the dataset. Documentation should also describe if and how missing data have been handled (e.g. imputation).

**1.12 Missing data**	**1.13 Missing data**	**1.3c Missing data**
Describe the proportion, nature and causes of missing data, particularly if there are systematic differences across relevant population groups. Describe how missing data has been identified and handled (e.g. Imputation, correction).<br>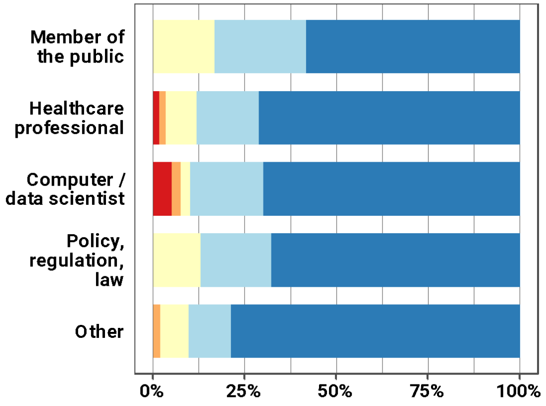<br><br>*Round 1 item 1.12*<br>3.1% vote to probably/definitely exclude<br>88.7% vote to probably/definitely include	*(numbering out of sync with round 1 because of previous items being split apart)*<br>Dataset documentation should describe the proportion, nature and causes of missing data (if known), particularly if there are systematic differences across relevant population groups. Documentation should also describe if missing data have been identified and how they have been handled (e.g. Imputation, correction).<br>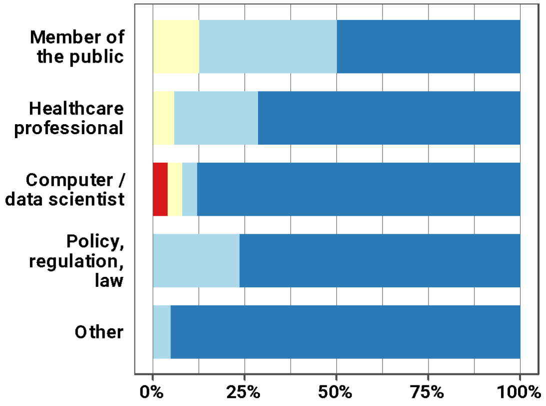<br><br>*Round 2 item 1.13*<br>0.9% vote to probably/definitely exclude<br>95.3% vote to probably/definitely include	Dataset documentation should describe the proportion, nature and causes of missing data (if known), particularly if there are systematic differences across relevant population groups. Documentation should also describe if missing data have been identified and how they have been handled (e.g. Imputation, correction).<br><br>**ACTIONS AT CONSENSUS MEETING**<br>- Vote: 96% include<br>- Item included in final recommendations<br><br>**AMENDMENTS FOLLOWING CONSENSUS MEETING**<br>- Minor reword for consistency of language with other items<br>- Removed reference to describing 'if missing data have been identified', as this is tautologous with first part of the item<br>- Removed inference that 'handling' missing data constitutes 'correction'

1.3d - Known or potential bias caused or exacerbated by data acquisition and processing

Dataset documentation should:

- Describe how bias may be introduced by the acquisition and processing of data within the dataset.
- Highlight any known or potential differences in data acquired across different groups, or differences in the uncertainty of measurements between groups.
- Describe any attempts to mitigate these biases.

<table>
<tr>
<td>

1.14 Known or potential bias in data generation

Describe how bias may be introduced by the acquisition and processing of data within the dataset, for example from the use of devices, sensors and software. Highlight any known or potential differences in data acquired across different population groups, or any uncertainty in performance within population groups.

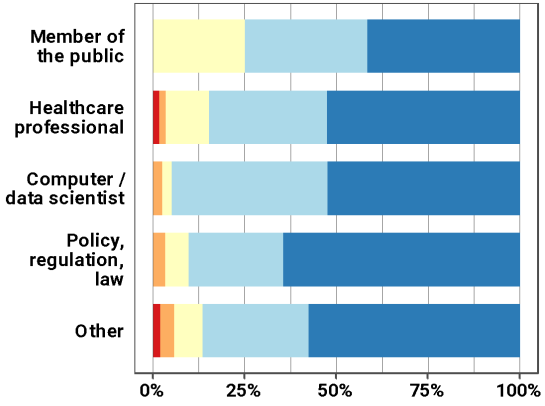

Round 1 item 1.14

3.6% vote to probably/definitely exclude
87.6% vote to probably/definitely include

</td>
<td>

1.15 Known or potential bias in data generation

Dataset documentation should:

- Describe how bias may be introduced by the acquisition and processing of data within the dataset. For example: from the use of devices, sensors and software.
- Highlight any known or potential differences in data acquired across different population groups, or any uncertainty of measurements within population groups.
- Describe any attempts to mitigate these biases.

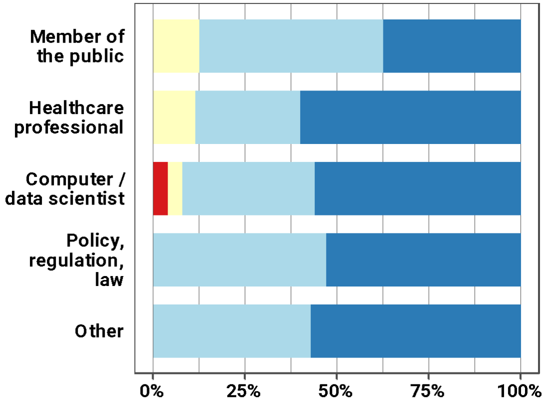

Round 2 item 1.15

0.9% vote to probably/definitely exclude
93.4% vote to probably/definitely include

</td>
<td>

1.3e Known or potential bias in data generation

Dataset documentation should:

- Describe how bias may be introduced by the acquisition and processing of data within the dataset. For example: from the use of devices, sensors and software.
- Highlight any known or potential differences in data acquired across different population groups, or any uncertainty of measurements within population groups.
- Describe any attempts to mitigate these biases.

<u>ACTIONS AT CONSENSUS MEETING</u>

- Discussion that there is redundancy between this item and the next two; request that this is addressed following consensus meeting even if all three items are included (i.e. to clearly differentiate the three)
- Minor reword of item title to '…data acquisition and processing'
- Vote: 81% include, 11% exclude, 8% abstain
- Included in final recommendations

<u>AMENDMENTS FOLLOWING CONSENSUS MEETING</u>

- Minor reword of title, adding 'caused or exacerbated'
- Minor changes of language in item text for consistency with other items
- Moved example to explanatory text

</td>
</tr>
</table>

1.3e - Known or potential exclusion introduced by data collection

Dataset documentation should:

- Identify the context of data collection and areas where exclusion may have been introduced into the data collection process.

- Describe any attempts to mitigate these biases.

**1.15 Known or potential bias in data collection**<br>Identify areas where bias may have been introduced into the data collection process. For example, only collecting data from one geographical area, only using questionnaires in English.<br>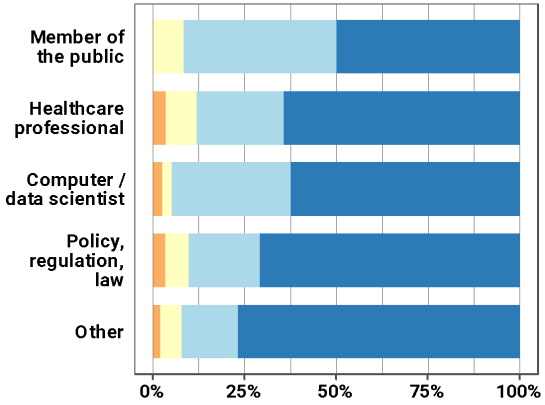<br>*Round 1 item 1.15*<br>2.6% vote to probably/definitely exclude<br>91.2% vote to probably/definitely include	**1.16 Known or potential bias in data collection**<br>Dataset documentation should:<br>- Identify areas where bias may have been introduced into the data collection process. For example: only collecting data from one geographical area, context regarding healthcare coverage and accessibility, only using questionnaires in English.<br>- Describe any attempts to mitigate these biases.<br>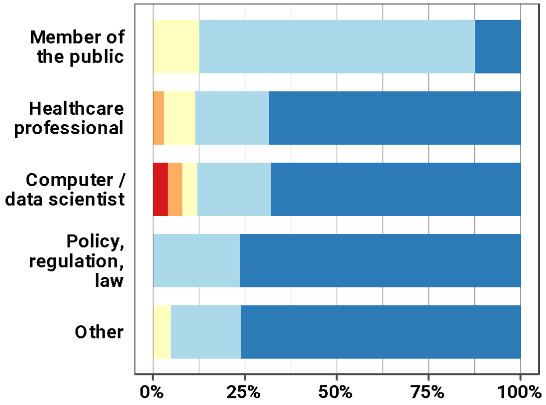<br>*Round 2 item 1.16*<br>2.8% vote to probably/definitely exclude<br>91.5% vote to probably/definitely include	**1.3f Known or potential bias in data collection**<br>Dataset documentation should:<br>▪ Identify areas where bias may have been introduced into the data collection process. For example: only collecting data from one geographical area, context regarding healthcare coverage and accessibility, only using questionnaires in english.<br>▪ Describe any attempts to mitigate these biases.<br><br>**<u>ACTIONS AT CONSENSUS MEETING</u>**<br>- Reframed item to talk about 'exclusion' rather than 'bias' more generally, to give more distinction between this and earlier items<br>- Replaced reference to 'english' to 'particular languages'<br>- Vote: 84% include, 8% exclude, 8% abstain<br>- Item included in final recommendations<br><br>**<u>AMENDMENTS FOLLOWING CONSENSUS MEETING</u>**<br>- Moved examples to explanatory text

1.3f - Known or potential bias in assigned or derived Labels

Dataset documentation should:

- Provide a description of any assigned or derived Labels, including who decided what Labels to include, what they were called, and how they were generated.
- Highlight Labels that are at high risk of bias. For example, where Label generation was at the discretion of individuals, where known biases in labelling behaviour have been evidenced previously, or in the use of proxy variables
- Describe any attempts to mitigate these biases.

1.16 Known or potential bias in data labels

Provide a description of any data labels, including who decided what labels to include, what they were called, and how they were generated. Highlight labels that are at high risk of bias. For example, where label generation was at the discretion of individuals, where known biases in labelling behaviour has been evidenced previously, or in the use of proxy variables (e.g., healthcare costs as a proxy of healthcare needs).

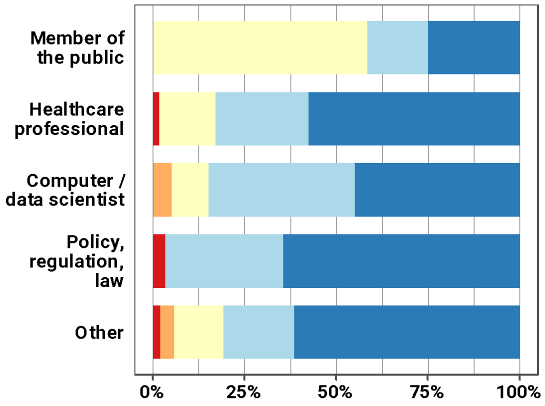

Round 1 item 1.16

3.6% vote to probably/definitely exclude
82.5% vote to probably/definitely include

1.17 Known or potential bias in data labels

Dataset documentation should:

- Provide a description of any data labels, including who decided what labels to include, what they were called, and how they were generated.
- Highlight labels that are at high risk of bias. For example, where label generation was at the discretion of individuals, where known biases in labelling behaviour has been evidenced previously, or in the use of proxy variables (e.g., healthcare costs as a proxy of healthcare needs)
- Describe any attempts to mitigate these biases.

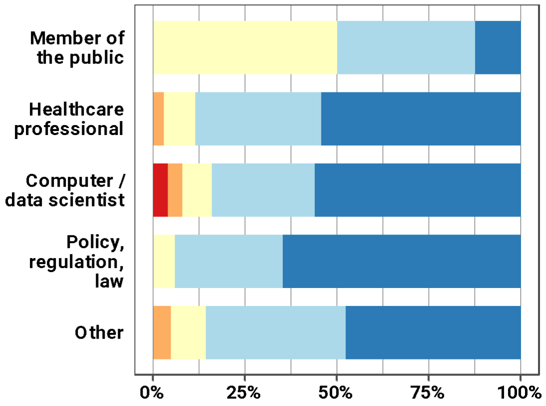

Round 2 item 1.17

3.8% vote to probably/definitely exclude
84.9% vote to probably/definitely include

1.3g Known or potential bias in data labels

Dataset documentation should:

- Provide a description of any data labels, including who decided what labels to include, what they were called, and how they were generated.
- Highlight labels that are at high risk of bias. For example, where label generation was at the discretion of individuals, where known biases in labelling behaviour has been evidenced previously, or in the use of proxy variables (e.g., healthcare costs as a proxy of healthcare needs)
- Describe any attempts to mitigate these biases.

<u>**ACTIONS AT CONSENSUS MEETING**</u>

- Amended title and item to reference assigned or derived labels
- Vote: 85% include, 11% exclude, 4% abstain
- Item included in final recommendations

<u>**AMENDMENTS FOLLOWING CONSENSUS MEETING**</u>

- Minor grammar changes
- Moved specific example to explanatory text for consistency with other items

1.4a - Ethics and governance

Dataset documentation should:

- State which data protection laws have been adhered to, and in which jurisdiction(s) they apply

- Describe measures taken to protect the identities of individuals.
- Describe permissions (including ethical, legal and institutional) obtained to enable dataset creation, and details of the governance of the dataset.
- Describe adherence to principles that respect data sovereignty for communities, where relevant.

**1.17 Ethics and data governance**	**1.18 Ethics, governance, and quality assurance**	**1.4a Ethics, governance, and quality assurance**
Describe the consent process for inclusion in the dataset, measures taken to protect identities of the individuals, permissions and governance of the dataset, and provide references to institutional review board/ethical committee review. Reference any standards (e.g. ISO, FAIR) which have been adhered to.<br>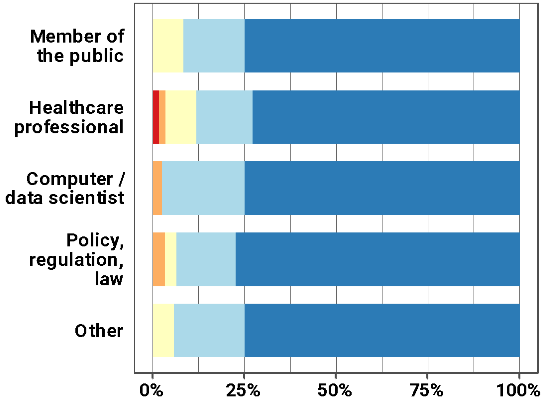<br>*Round 1 item 1.17*<br>2.1% vote to probably/definitely exclude<br>92.8% vote to probably/definitely include	Dataset curators should describe in their documentation whether data protection laws specific to their jurisdiction have been adhered to. Dataset documentation should also:<br>- Describe measures taken to protect the identities of individuals.<br>- Describe permissions obtained to enable dataset curation, and details of the governance of the dataset.<br>- Provide references to institutional review board/ethical committee review (or equivalent, as appropriate).<br>- Reference standards (e.g. ISO, FAIR) which have been adhered to.<br>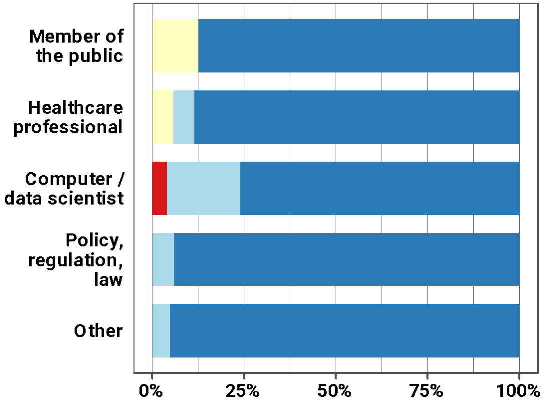<br>*Round 2 item 1.18*<br>0.9% vote to probably/definitely exclude<br>96.2% vote to probably/definitely include	Dataset curators should state in their documentation whether data protection laws specific to their jurisdiction have been adhered to. Dataset documentation should also:<br>▪ Describe measures taken to protect the identities of individuals.<br>▪ Describe permissions obtained to enable dataset curation, and details Of the governance of the dataset.<br>▪ Provide references to institutional review board/ethical committee review (or equivalent, as appropriate).<br>▪ Reference standards (e.g. ISO, FAIR) which have been adhered to.<br><br>**ACTIONS AT CONSENSUS MEETING**<br>- Removed reference to 'quality assurance' from item title<br>- Minor rewording of item text<br>- Vote: 100% include<br>- Item included in final recommendations<br><br>**AMENDMENTS FOLLOWING CONSENSUS MEETING**<br>- Minor grammar & wording changes for clarity<br>- Moved references to IRB & review processes, and to ISO to explanatory text<br>- Removed reference to FAIR and added data sovereignty to reduce item overlap

1.4b - Patient and public participation

Dataset documentation should:

- Describe the role of any advisory boards and patient and public participation groups.
- Provide information on any efforts to share data and findings with those who contributed to the dataset and any feedback gathered from participants that is relevant to data interpretation.

1.18 Patient and public involvement and engagement
Describe the role of advisory boards and patient and public involvement and engagement groups in the dataset curation. Provide information on efforts to share data and findings with those who contributed to the dataset and any feedback that was gathered from participants that is relevant to data interpretation.

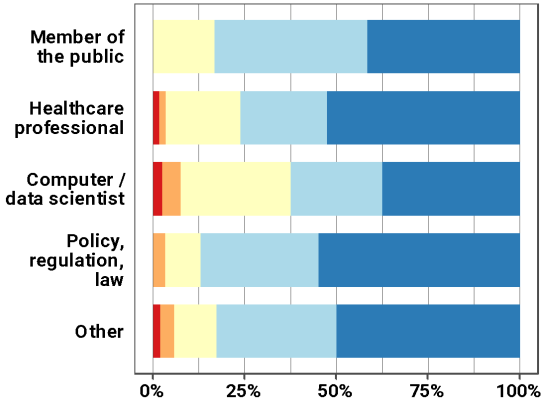

Round 1 item 1.18

4.6% vote to probably/definitely exclude
77.3% vote to probably/definitely include

1.19 Patient and public involvement and engagement
Dataset documentation should:

- Describe the role of any advisory boards and patient and public involvement and engagement groups in the dataset curation.
- Provide information on any efforts to share data and findings with those who contributed to the dataset and any feedback gathered from participants that is relevant to data interpretation.

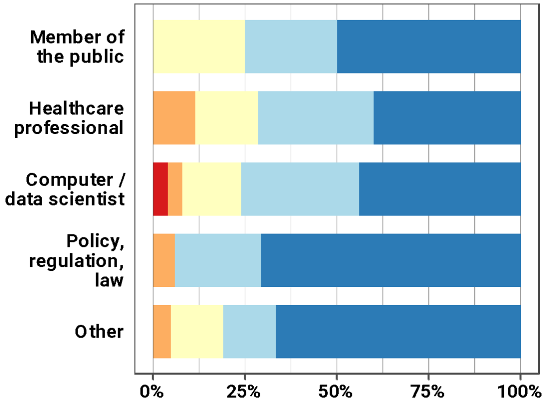

Round 2 item 1.19

7.5% vote to probably/definitely exclude
78.3% vote to probably/definitely include

1.4b Patient and public involvement and engagement
Dataset documentation should:

- Describe the role of any advisory boards and patient and public involvement and engagement groups in the dataset curation.
- Provide information on any efforts to share data and findings with those who contributed to the dataset and any feedback gathered from participants that is relevant to data interpretation.

ACTIONS AT CONSENSUS MEETING

- Minor rewording
- Vote: 88% include, 4% exclude, 8% abstain
- Item included in final recommendations

AMENDMENTS FOLLOWING CONSENSUS MEETING

- Introduced 'patient and public participation' in lieu of 'patient and public involvement and engagement' to align with terminology used by international bodies, and because 'involvement' and 'engagement' describe discrete activities whereas 'participation' is more holistic.

1.4c - Bias and impact assessments

If a formal assessment of bias, fairness or societal impact has been previously conducted on the dataset, dataset documentation should include these assessments and results.

1.19 Bias and impact assessments

If a formal assessment of bias, fairness or societal impact has been previously conducted on the dataset, please provide the assessment and results. This may include algorithmic impact assessments (aias), data protection impact assessments (dpias), equality impact assessments, documentation tools, risk of bias assessments or automated toolkits.

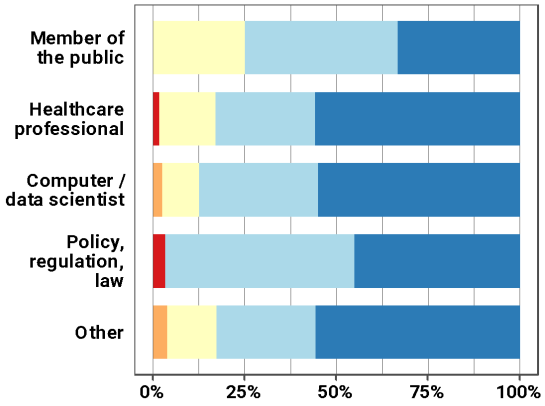

Round 1 item 1.19

2.6% vote to probably/definitely exclude
85.6% vote to probably/definitely include

1.20 Bias and impact assessments

If a formal assessment of bias, fairness or societal impact has been previously conducted on the dataset, dataset documentation should provide these assessments and results. This may include algorithmic impact assessments (AIAs), data protection impact assessments (DPIAs), equality impact assessments, documentation tools, risk of bias assessments or automated toolkits.

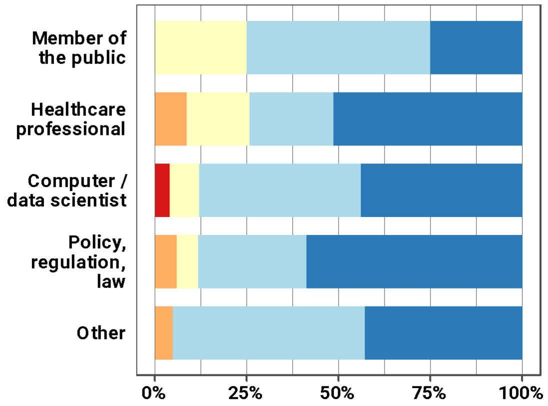

Round 2 item 1.20

5.7% vote to probably/definitely exclude
84.0% vote to probably/definitely include

1.4c Bias and impact assessments

If a formal assessment of bias, fairness or societal impact has been previously conducted on the dataset, dataset documentation should provide these assessments and results. This may include algorithmic impact assessments (AIAs), data protection impact assessments (DPIAs), equality impact assessments, documentation tools, risk of bias assessments or automated toolkits.

ACTIONS AT CONSENSUS MEETING

- Discussion around whether specific toolkits should be included. Clear feeling that the examples should be moved to explanatory text.
- Vote on item without specific toolkits mentioned: 96% include, 4% exclude
- Item included in final recommendations

AMENDMENTS FOLLOWING CONSENSUS MEETING

- N/A

2.1a - Provide sufficient information about dataset(s) to allow traceability and auditability

Datasets used in the lifecycle of AI health technologies should be accompanied by documentation which conforms to **Recommendations for Dataset Documentation** (See Section 1), enabling auditing against these standards.

2.1

Full documentation of datasets used in the lifecycle of AI health technologies should be provided to enable audit against these standards.

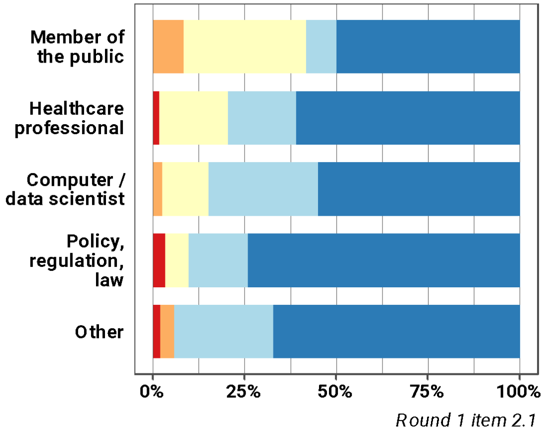

3.6% vote to probably/definitely exclude
85.1% vote to probably/definitely include

2.1 Provide sufficient information about dataset(s) to allow traceability and auditability

Datasets used in the lifecycle of AI health technologies should be accompanied by documentation which conforms to items 1.1-1.20, enabling audit against these standards.

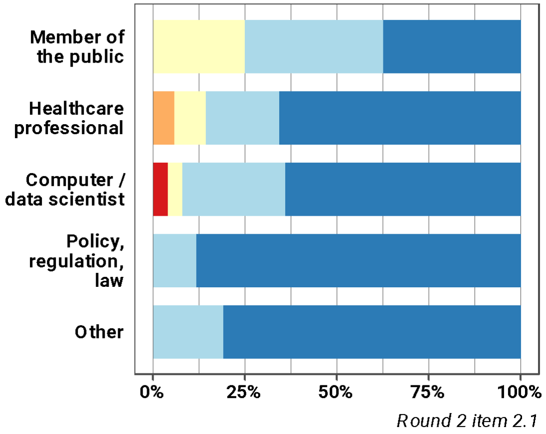

2.8% vote to probably/definitely exclude
91.5% vote to probably/definitely include

2.1a Provide sufficient information about dataset(s) to allow traceability and auditability

Datasets used in the lifecycle of AI health technologies should be accompanied by documentation which conforms to dataset documentation standards, enabling audit against these standards.

ACTIONS AT CONSENSUS MEETING

- Discussion about the scope of this item – should it apply to all datasets or only those used for testing?
- Request to add rationale and expansion in the explanatory text
- Vote: 92% include, 8% exclude
- Item included in final recommendations

AMENDMENTS FOLLOWING CONSENSUS MEETING

- Minor change to update the reference to section 1 of recommendations

2.2a - Identify Contextualised Groups of Interest in advance who may be at risk of disparate performance or harm from the AI health technology

Data Users should identify Contextualised Groups of Interest in advance. These may be identified in various ways, including evidence appraisal and literature review, collaboration with domain experts in the Intended Use of the AI health technology, consultation with those who have lived experience, and evidence generation and discovery through data analysis and algorithm testing.

2.2

Data Users should identify contextualised subgroups of interest in advance: these are subgroups with shared attributes, identified as being relevant and important for the use case, and where they are known to have worse health outcomes or are subject to other systems driving health inequity related to the use case.

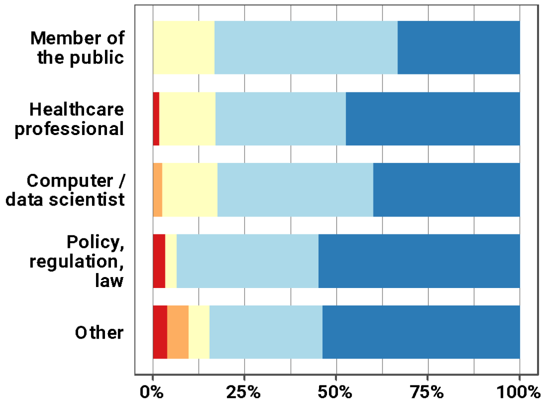

Round 1 item 2.2

4.1% vote to probably/definitely exclude
85.1% vote to probably/definitely include

2.3

(merged with 'Round 1 item 2.2' to create new 'Round 2 item 2.2')

Data Users should identify and document in the literature review evidence of discrepant performance across any subgroups in similar devices.

2.2 Identify contextualised subgroups of interest who are particularly at risk of harm from the AI health technology under development

(combination of 'Round 1 item 2.2' and 'Round 1 item 2.3')

Data Users should identify contextualised subgroups of interest in advance: these are subgroups with shared attributes, identified as being relevant and important for the use case, and where they are known to have worse health outcomes or are subject to other systems driving health inequity related to the use case. Contextualised subgroups of interest may be discovered via multiple sources, including literature review, evidence from the development or use of similar AI health technologies, consultation with experts in health inequity, clinical practice, etc.

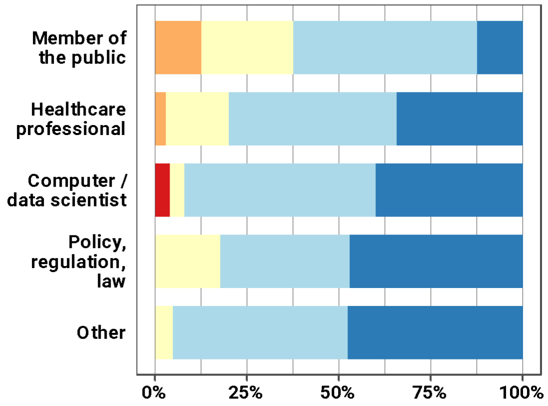

Round 2 item 2.2

2.8% vote to probably/definitely exclude
85.9% vote to probably/definitely include

2.2a Identify contextualised subgroups of interest who are particularly at risk of harm from the AI health technology under development

Data users should identify contextualised subgroups of interest in advance: these are subgroups with shared attributes, identified as being relevant and important for the use case, and where they are known to have worse health outcomes or are subject to other systems driving health inequity related to the use case. Contextualised subgroups of interest may be discovered via multiple sources, including literature review, evidence from the development or use of similar AI health technologies, consultation with experts in health inequity, clinical practice, etc.

ACTIONS AT CONSENSUS MEETING

- Request to reframe 'contextualised (sub)groups of interest' as those where performance may vary, rather than specifically being worse
- Request to specifically define harm in explanatory text
- Specify in title that groups should be identified in advance
- Vote: 92% include, 4% exclude, 4% abstain
- Item included in final recommendations

AMENDMENTS FOLLOWING CONSENSUS MEETING

- Definition of 'Contextualised Groups of Interest' moved to explanatory text & glossary
- Rewording of item text & title for clarity of message
- Definition of harm added to glossary

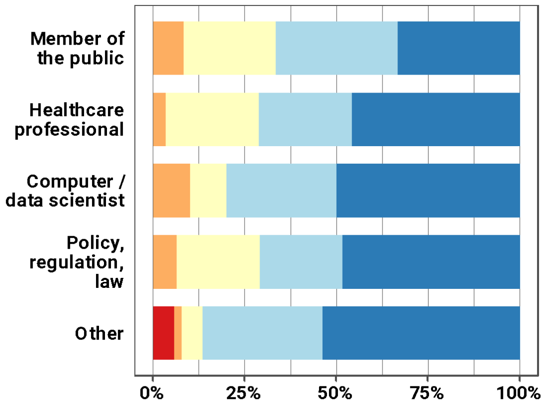<br>*Round 1 item 2.3*<br>6.7% vote to probably/definitely exclude<br>76.8% vote to probably/definitely include		- Expanded list of ways 'Contextualised Groups of Interest' can be identified

2.2b - Justify that datasets have been used appropriately to support the Intended Use Population, and Intended Use of the AI health technology

The Intended Use Population should be appropriately represented in datasets used in an AI health technology. The Contextualised Groups of Interest (i.e. those who may be at risk of disparate performance or harm, see item 2.2a) should also be included where possible, and if not included this should be explicitly stated by Data Users. Areas of under-representation should be identified and transparently reported by Data Users.

2.6

The intended use population of the AI Health Technology should be adequately represented in the training and test datasets for an AI Health Technology. The contextualised subgroups of interest should also be included where possible, and if not, explicitly stated. Areas of under-representation should be identified and transparently reported.

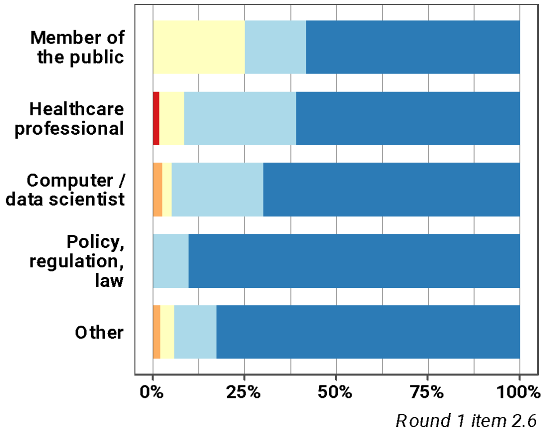

1.5% vote to probably/definitely exclude
93.3% vote to probably/definitely include

2.3 Use appropriate datasets to support the intended use population, and intended purpose of the AI health technology

(order changed following delphi round 1)

The intended use population should be adequately represented in the datasets used in an AI Health Technology. The contextualised subgroups of interest (see item 2.3) should also be included where possible, and if not, explicitly stated. Areas of under-representation should be identified and transparently reported.

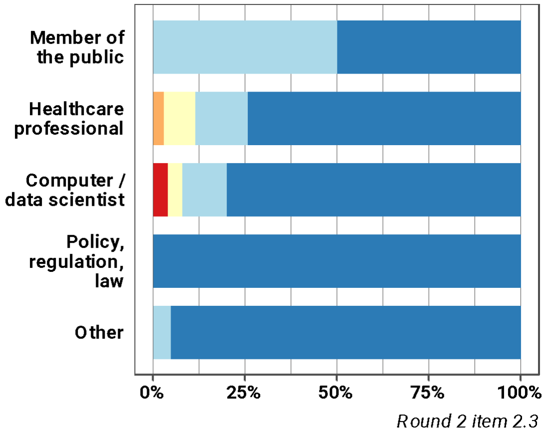

1.9% vote to probably/definitely exclude
94.3% vote to probably/definitely include

2.2b Use appropriate datasets to support the intended use population, and intended purpose of the AI health technology

The intended use population should be adequately represented in the datasets used in an AI health technology. The contextualised subgroups of interest (see item 2.2a) should also be included where possible, and if not included this should be explicitly stated. Areas of under-representation should be identified and transparently reported.

ACTIONS AT CONSENSUS MEETING

- Amended title to request that data users ‘justify’ their choice of datasets
- Changed language from ‘adequately’ to ‘appropriately’ represented
- Request to clarify that the onus is on data users to justify that dataset representation is appropriate
- Vote: 96% include, 4% abstain
- Item included in final recommendations

AMENDMENTS FOLLOWING CONSENSUS MEETING

- Minor rewording for consistency of language with other items, and to clarify that the responsibility to justify is that of data users

2.2c Report the explicit and implicit use of Relevant Attributes during the lifecycle of the AI health technology
Data Users should report whether and how any Relevant Attributes were used during the lifecycle of the AI health technology, including as a feature, proxy or label.

		**2.** Report the explicit and implicit use of relevant attributes during the development of the AI health technology**<br>Data Users should report whether and how any relevant attributes were used in the development of the AI health technology, including as a feature, proxy or label.<br><br>**ACTIONS DURING CONSENSUS MEETING**<br>- New item proposed by attendees<br>- Vote: 92% include, 8% exclude<br>- Item included in final recommendations<br><br>**AMENDMENTS FOLLOWING CONSENSUS MEETING**<br>Replaced 'development' with 'lifecycle' to widen scope of the item.

2.2d - Evaluate performance of the AI health technology for Contextualised Groups of Interest

Data users should report performance of the AI health technology for Contextualised Groups of Interest identified in 2.2a, to enable comparison of performance for each group versus aggregate performance across the overall study population, and comparison of performance between different Contextualised Groups of Interest.

**2.4**	**2.4 Identify discrepant performance of the AI health technology for contextualised subgroups of interest**	**2.2c Identify discrepant performance of the AI health technology for contextualised subgroups of interest**
Having identified contextualised subgroups of interest, Data Users should report evaluation results of the AI Health Technology within those subgroups, in addition to aggregate results of evaluation.<br>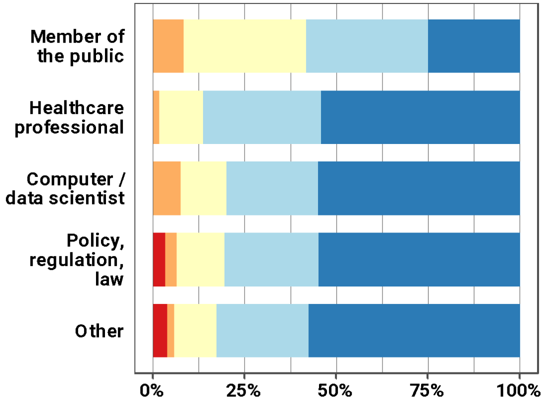<br>*Round 1 item 2.4*<br>5.2% vote to probably/definitely exclude<br>81.4% vote to probably/definitely include	Data Users should:<br>- Report performance of the AI Health Technology for contextualised subgroups of interest identified in 2.3.<br>- Compare performance for contextualised subgroups of interest to aggregate performance in the overall study population.<br>Report performance of the AI Health Technology for subgroup(s) who have the best pre-existing health outcomes in this clinical area, and compare this to performance for contextualised subgroups of interest.<br>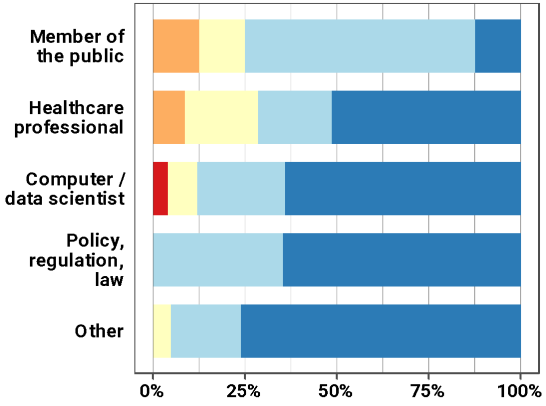<br>*Round 2 item 2.4*<br>4.7% vote to probably/definitely exclude<br>84.9% vote to probably/definitely include	Data users should:<br>▪ Report performance of the AI health technology for contextualised subgroups of interest identified in 2.2a.<br>▪ Compare performance for contextualised subgroups of interest to aggregate performance in the overall study population.<br>▪ Report performance of the ai health technology for subgroup(s) who have the best pre-existing health outcomes in this clinical area, and compare this to performance for contextualised subgroups of interest.<br><br>**ACTIONS AT CONSENSUS MEETING**<br>- Amended item wording to account for new definition of 'Contextualised (sub)Groups of Interest'<br>- Vote: 100% include<br>- Item included in final recommendations<br><br>**AMENDMENTS FOLLOWING CONSENSUS MEETING**<br>- Simplified item title<br>- Minor grammar change to removed bulleted list from item for consistency with other items in this section.

2.2e - Identify disparate performance in any additional groups outside of the pre-specified contextualised groups of interest
As well as conducting pre-specified evaluation of performance in Contextualised Groups of Interest (2.2a and 2.2c), Data Users should also evaluate performance across other groups to identify disparate performance of the AI health technology which were not previously anticipated, and may lead to harm.

2.5
Data Users should also report evaluation results across certain attributes (including age, gender, sex, race, ethnicity, socioeconomic status), due to known associations with health outcomes and interactions with wider social factors. This may not always be possible or appropriate, in which case the reasons for not doing so should be documented.

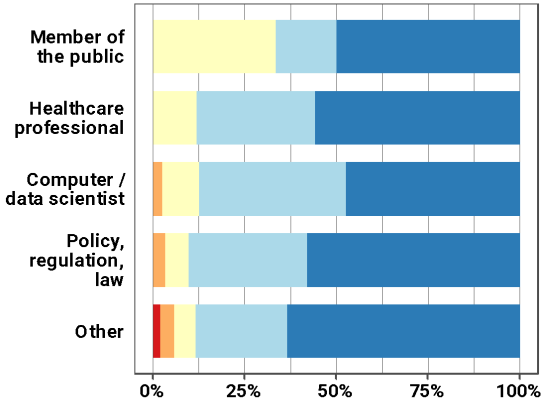

Round 1 item 2.5

2.6% vote to probably/definitely exclude
87.1% vote to probably/definitely include

2.5 Evaluate performance of the AI health technology for subgroups experiencing vulnerability
If not already addressed by 2.4, Data Users should report evaluation results across certain attributes (including age, gender identity, sex, race, ethnicity, socioeconomic status and sexual orientation), due to known associations with health outcomes and interactions with wider social factors. This may not always be possible or appropriate, in which case the reasons for not doing so should be documented.

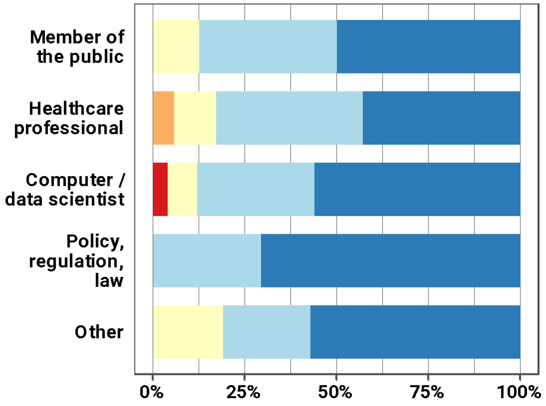

Round 2 item 2.5

2.8% vote to probably/definitely exclude
86.8% vote to probably/definitely include

2.2d Evaluate performance of the AI health technology for subgroups experiencing vulnerability
If not already addressed by 2.2c, data users should report evaluation results across certain attributes (including age, gender identity, sex, race, ethnicity, socioeconomic status and sexual orientation), due to known associations with health outcomes and interactions with wider social factors. This may not always be possible or appropriate, in which case the reasons for not doing so should be documented.

<u>ACTIONS AT CONSENSUS MEETING</u>
- Moved specific attributes from item to explanatory text in accordance with earlier vote
- Changed vulnerability to 'subgroups experiencing disparate health outcomes'
- Vote: 100% include
- Included in final recommendations

<u>AMENDMENTS FOLLOWING CONSENSUS MEETING</u>
- Reframed item title and text to address overlap of this item with others following redefinition of 'Contextualised (sub)Groups of Interest'; clarifyied that the task is to perform exploratory post-hoc analysis rather than repeating the evaluation for 'Contextualised Groups of Interest'.

2.2f Report any approaches or methods (including fairness methods) used to intentionally modify performance across groups.
Data users should document any attempts during the lifecycle of the AI health technology which attempt to modify performance, including addressing disparate performance across groups. If applicable, explain the rationale and goals for doing so, the methods and metrics used, whether/how thresholds were set and whether these varied between groups.

-	**2.## - Report any statistical approaches (including 'fairness methods/metrics') used to intentionally modify performance across subgroups.**<br>Data users should document any attempts during development and evaluation of the ai health technology, which attempt to make predictions more equitable across subgroups. Describe:<br>▪ The rationale and goals for doing so.<br>▪ The methods and metrics used.<br>▪ How thresholds were set, including whether these vary between subgroups of people.<br>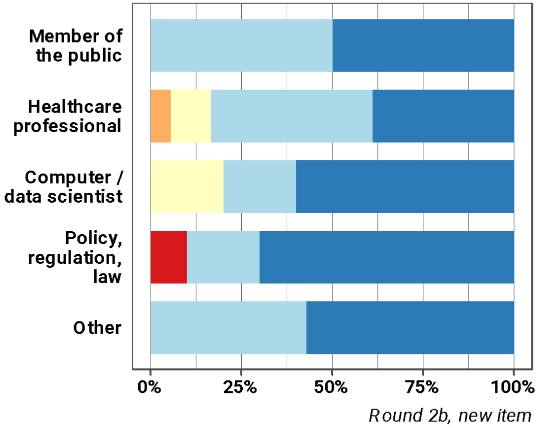<br>2.8% vote to probably/definitely exclude<br>90.4% vote to probably/definitely include	**2.## Report any statistical approaches (including 'fairness methods/metrics') used to intentionally modify performance across subgroups.**<br>Data users should document any attempts during development and evaluation of the AI health technology, which attempt to make predictions more equitable across subgroups. Describe:<br>▪ The rationale and goals for doing so.<br>▪ The methods and metrics used.<br>▪ How thresholds were set, including whether these vary between subgroups of people.<br><br>**<u>ACTIONS AT CONSENSUS MEETING</u>**<br>- General support for item<br>- Vote: 100% include<br>- Item included in final recommendations<br><br>**<u>AMENDMENTS FOLLOWING CONSENSUS MEETING</u>**<br>- Changed 'development and evaluation' to the more precise term 'lifecycle'<br>- Changed 'attempt to make predictions more equitable' to 'modify performance, including addressing disparate performance across groups', recognising that the intent may vary<br>- Minor changes to language and grammar, including removing bullet points for consistency with other items

2.3a - Report limitations of datasets used, and any implications on the AI health technology.
Data Users should report the limitations of datasets used, and any implications with reference to the Intended Use of the AI health technology. Data Users should investigate whether these limitations are systematically different across relevant groups, including those with attributes categorised as ‘unknown’, ‘prefer not to say’, or ‘other’, and report differences which could result in disparate performance of the AI health technology across groups.

2.7
Data Users should report limitations of the dataset and the implications on the AI Health Technology. Data Users should investigate whether limitations are systematically different across relevant population subgroups, including those categorised as ‘unknown’ or ‘other’, and report differences which could result in worse performance on the AI Health Technology across groups.

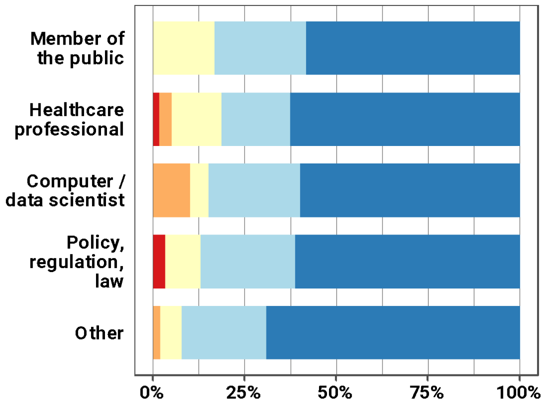

Round 1 item 2.7

4.6% vote to probably/definitely exclude
86.1% vote to probably/definitely include

2.6 Report limitations of datasets used, and any implications on the AI health technology
(item numbering out of sync because of combination of earlier items together)
Data Users should report limitations of the dataset and the implications on the target AI health technology. Data Users should investigate whether limitations are systematically different across relevant population subgroups, including those categorised as ‘unknown’ or ‘other’, and report differences which could result in worse performance on the AI Health Technology across groups.

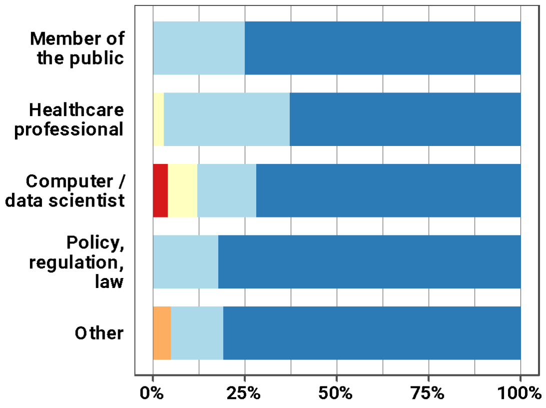

Round 2 item 2.6

1.9% vote to probably/definitely include
95.3% vote to probably/definitely include

2.3a Report limitations of datasets used, and any implications on the AI health technology
Data users should report limitations of datasets used, and the implications of these on the target AI health technology. Data users should investigate whether limitations are systematically different across relevant population subgroups, including those categorised as ‘unknown’ or ‘other’, and report differences which could result in worse performance on the ai health technology across groups.

ACTIONS AT CONSENSUS MEETING

- Add ‘prefer not to say’
- Minor rewording of item
- Vote: 96% include, 4% abstain
- Item included in final recommendations

AMENDMENTS FOLLOWING CONSENSUS MEETING

- Minor rewording for clarity and consistency with other items

2.3b - Report differences between the intended purposes of the AI health technology and datasets used, including the implications of discordance.
Data Users should report the purpose(s) of datasets used (see 1.1c), and how these differ from the Intended Use of the AI health technology (see 2.2b). The implications of any discordance and how this affects the suitability of the dataset for its role should be stated.

**2.8**<br>State any intended purposes of the dataset, and how this differs from the intended use of the AI Health Technology. State implications of any discordance and provide justification regarding the suitability of the dataset, including assumptions made and aspects of the dataset which are not directly applicable.<br>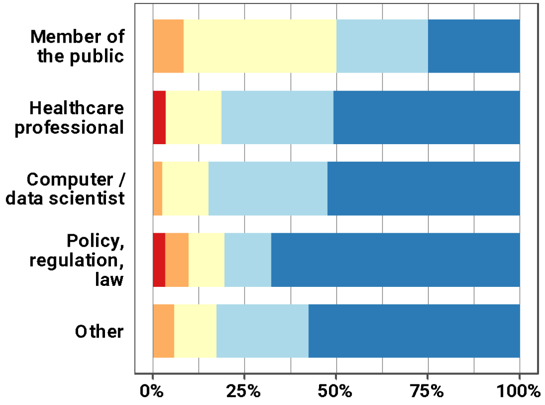<br><br>*Round 1 item 2.8*<br>5.2% vote to probably/definitely exclude<br>80.4% vote to probably/definitely include	**2.7 Report differences between the intended purposes of the AI health technology and datasets used during development, including the implications of discordance.**<br>Data users should report any intended purposes of datasets used (item 1.3), and how these differ from the intended purpose of the AI Health Technology (item 2.3). State implications of any discordance and provide justification regarding the suitability of the dataset, including assumptions made and aspects of the dataset which are not directly applicable.<br>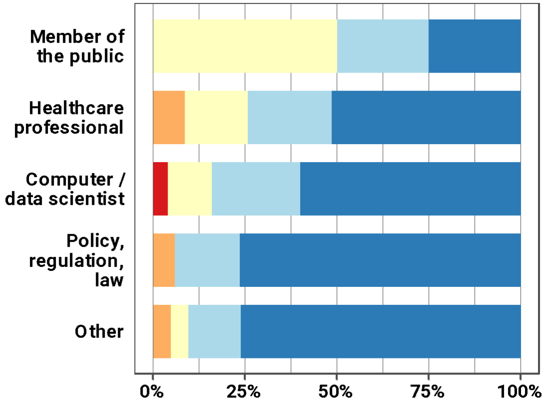<br><br>*Round 2 item 2.7*<br>5.7% vote to probably/definitely include<br>81.1% vote to probably/definitely include	**2.3b Report differences between the intended purposes of the AI health technology and datasets used during development, including the implications of discordance.**<br>Data users should report any intended purposes of datasets used (item 1.1C), and how these differ from the intended purpose of the AI Health Technology (item 2.2B). State implications of any discordance and provide justification regarding the suitability of the dataset, including assumptions made and aspects of the dataset which are not directly applicable.<br><br>**ACTIONS AT CONSENSUS MEETING:**<br>- Vote: 88% include, 12% abstain; quorum not met<br>- Re-discussed: request to provide elaboration in the explanatory text<br>- Re-voted: 92% include, 8% abstain<br>- Item included in final recommendations<br><br>**AMENDMENTS FOLLOWING CONSENSUS MEETING**<br>- Removed ‘during development’ from item title to widen scope of the item<br>- Minor reword for clarity of message and consistency with other items

2.3c - Report findings from pre-existing assessments of the AI health technology and any datasets used.
Data Users should review any available pre-existing assessments of both the AI health technology and any datasets used, and report how the findings may have implications on groups within the Intended Use Population, including risk of harm.

2.10
Data Users should review pre-existing assessments of the datasets (e.g. Algorithmic impact assessments, equality impact assessments, data protection impact assessments, datasheets, healthsheets) and report how the findings may translate to harm for subgroups within the intended use population.

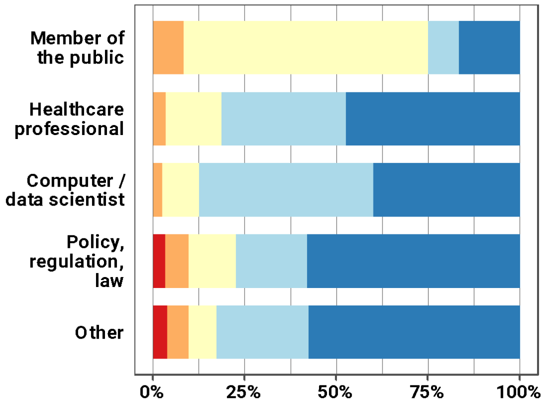

Round 1 item 2.10

6.2% vote to probably/definitely exclude
78.9% vote to probably/definitely include

2.9 Report findings of pre-existing dataset assessments
Data Users should review any pre-existing assessments of the datasets which are available (e.g. Algorithmic impact assessments, equality impact assessments, data protection impact assessments, datasheets, healthsheets) and report how the findings may translate to harm for subgroups within the intended use population.

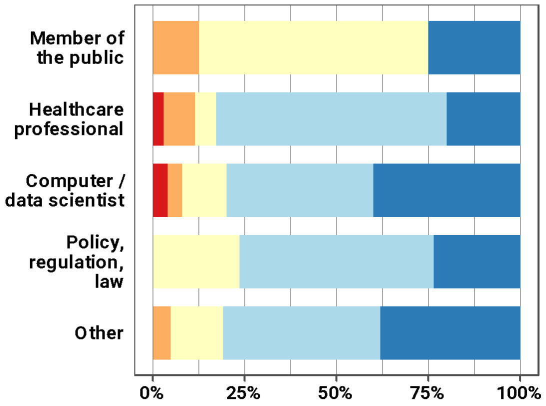

Round 2 item 2.9

7.5% vote to probably/definitely include
76.4% vote to probably/definitely include

2.3d Report findings of pre-existing dataset assessments
Data users should review any pre-existing assessments of the datasets which are available (e.g. Algorithmic impact assessments, equality impact assessments, data protection impact assessments, datasheet, healthsheet) and report how the findings may translate to harm for subgroups within the intended use population.

<u>ACTIONS AT CONSENSUS MEETING</u>
- Request to move specific tools to expanatory text
- Minor rewording of item text
- Vote: 92% include, 4% exclude, 4% abstain
- Item included in final recommendations

<u>AMENDMENTS FOLLOWING CONSENSUS MEETING</u>
- Widened scope of item to include assessments of the AI health technology as well as datasets
- Minor wording changes for consistency with other items
- Clarified that only available assessments are requested to be shared – this item is not a recommendation to undertake assessments

2.4a - Address uncertainties and risks with mitigation plans.

Where Data Users have identified uncertainty or potentially variable performance in groups, any clinical implications resulting from these findings must be clearly stated and reported as risks. The Data User should document strategies to monitor, manage and reduce these risks as part of the implementation of the AI health technology.

<table>
<tr>
<td>2.11
Where Data Users have identified uncertainty or potentially variable performance in subgroups, this should be identified and reported as a risk. If the Data User is a manufacturer of an AI health technology, risks must be monitored as part of the post market clinical follow-up and post market surveillance plans.
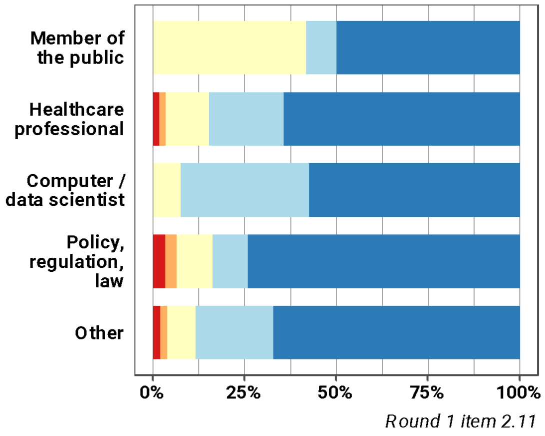

3.1% vote to probably/definitely exclude
85.6% vote to probably/definitely include</td>
<td>2.10 Address uncertainties and risks with mitigation plans
Where Data Users have identified uncertainty or potentially variable performance in subgroups, any clinical implications resulting from these findings must be clearly stated and reported as risks. The Data User should document plans to monitor these risks as part of the post market clinical follow-up and post market surveillance.
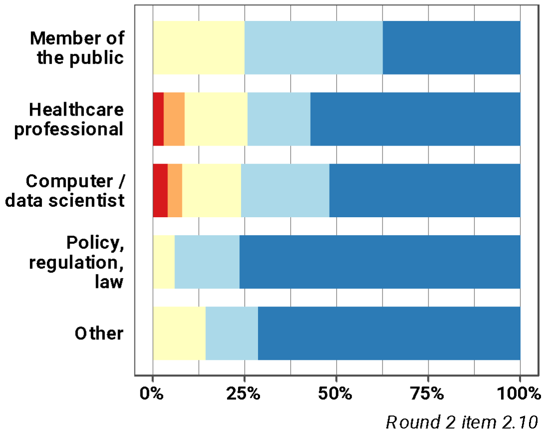

4.7% vote to probably/definitely include
80.2% vote to probably/definitely include</td>
<td>2.4a Address uncertainties and risks with mitigation plans
Where data users have identified uncertainty or potentially variable performance in subgroups, any clinical implications resulting from these findings must be clearly stated and reported as risks. The data user should document plans to monitor these risks as part of the post-market clinical follow-up and post-market surveillance.

<u>ACTIONS AT CONSENSUS MEETING</u>
- Amended language of item to say data users should ‘monitor, manage and reduce’ risks
- Vote: 92% include, 4% exclude, 4% abstain
- Item included in final recommendations

<u>AMENDMENTS FOLLOWING CONSENSUS MEETING</u>
- Minor wording changes for consistency with other items</td>
</tr>
</table>

**** ITEM REMOVED BY CONSENSUS MEETING ATTENDEES ****

1.4 Dataset curation team

Describe how the diversity of those represented within the dataset are reflected in the curation team, to ensure assumptions and preconceptions are considered. This may involve attributes of the dataset curators themselves, as well as governing and consultation groups (e.g. Advisory boards, patient and public involvement and engagement groups).

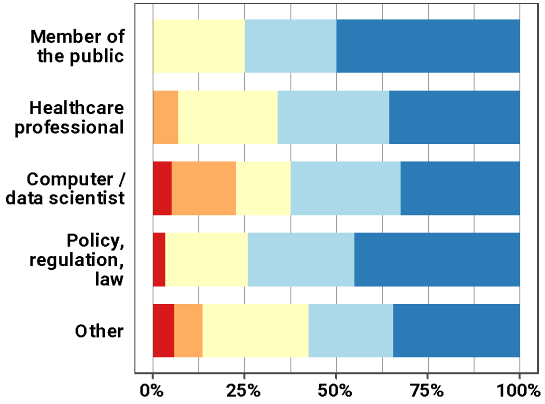

Round 1 item 1.4

10.8% vote to probably/definitely exclude
65.0% vote to probably/definitely include

1.4 Assumptions and preconceptions of the dataset curation team

Dataset documentation should describe how the curation team has considered the impact of their prior assumptions and preconceptions on biases in the dataset. This may include reflecting on the experiences of the dataset curators themselves, as well as any advice from governing and consultation groups (e.g. Advisory boards, patient and public involvement and engagement groups).

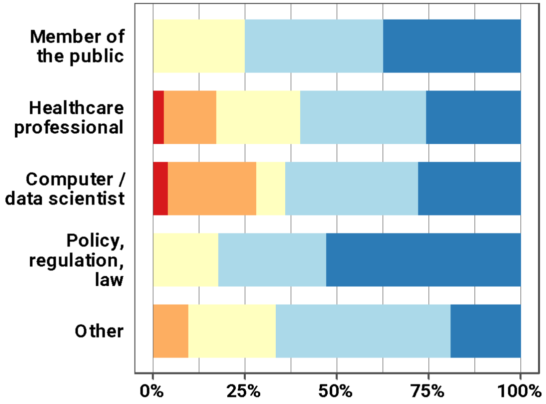

Round 2 item 1.4

14.2% vote to probably/definitely include
67.0% vote to probably/definitely include

1.1d Assumptions and preconceptions of the dataset curation team

Dataset documentation should describe how the curation team has considered the impact of their prior assumptions and preconceptions on biases in the dataset. This may include reflecting on the experiences of the dataset curators themselves, as well as any advice from governing and consultation groups (e.g. Advisory boards, patient and public involvement and engagement groups).

ACTIONS AT CONSENSUS MEETING

- Extensive discussion, but clear majority view that item should be removed, and content merged with other items or their explanatory text.
- Vote: 20% include, 64% exclude, 16% abstain
- Item removed

**** ITEM REMOVED BY CONSENSUS MEETING ATTENDEES ****

1.13 Errors in the data

Describe how errors can be/have been identified in the data and how they have been handled (e.g. Have they been removed, modified, corrected or left in the dataset). Provide an estimation of the proportion of errors that are present and whether they are more prevalent in some population groups than others. Provide possible reasons for any systematic differences in error rates across population groups within the dataset.

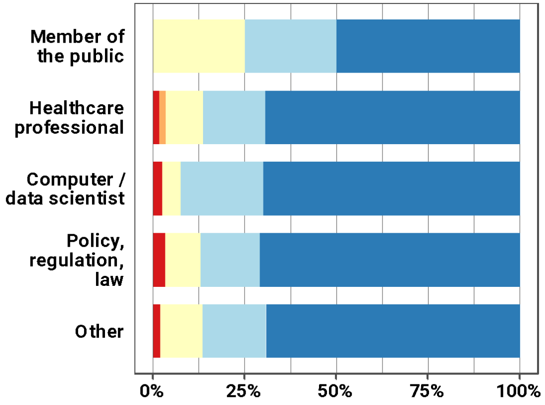

Round 1 item 1.13

2.6% vote to probably/definitely exclude
87.1% vote to probably/definitely include

1.14 Errors in the data

Dataset documentation should:

- Describe how errors can be/have been identified in the data and how they have been handled (e.g. Have they been removed, modified, corrected or left in the dataset).
- Provide an estimation of the proportion of errors that are present and whether they are more prevalent in some population groups than others.
- Provide possible reasons for any systematic differences in error rates across population groups within the dataset.

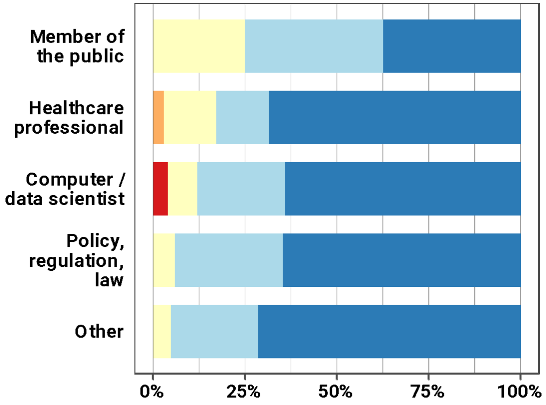

Round 2 item 1.14

1.9% vote to probably/definitely include
87.7% vote to probably/definitely include

1.3d Errors in the data

Dataset documentation should:

- Describe how errors can be/have been identified in the data and how they have been handled (e.g. Have they been removed, modified, corrected or left in the dataset).
- Provide an estimation of the proportion of errors that are present and whether they are more prevalent in some population groups than others.
- Provide possible reasons for any systematic differences in error rates across population groups within the dataset.

<u>ACTIONS AT CONSENSUS MEETING</u>

- Extensive discussion, highlighting that the concept of 'error' was hard to definitively define. Clear feeling that the item should be excluded and concept of 'error' moved to other items or their explanatory text instead
- Vote: 65% include, 27% exclude, 8% abstain
- Item removed

**** ITEM REMOVED BY CONSENSUS MEETING ATTENDEES ****

**2.9**	**2.8 Report level of uncertainty for performance in subgroups when sample size is insufficient**	**2.3c Report level of uncertainty for performance in subgroups when sample size is insufficient**
In the case of minority and intersectional population groups, achieving sufficient sample size may not be possible. Data Users should report the level of uncertainty for performance in these subgroups (e.g. With confidence intervals). Where this may confer additional risk, mitigation plans should be in place to avoid harm to these groups.<br>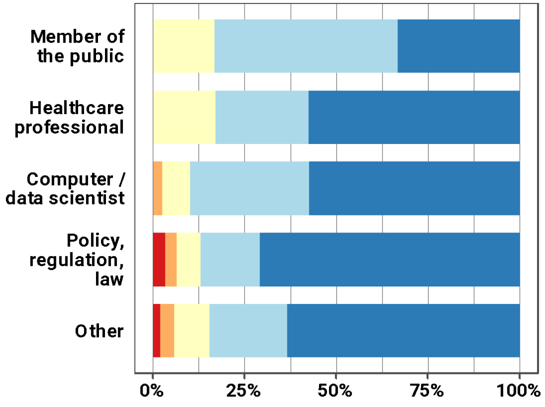<br>*Round 1 item 2.9*<br>3.1% vote to probably/definitely exclude<br>85.6% vote to probably/definitely include	Should sufficient sample size not be achieved in minority and/or intersectional subgroups, Data Users should report the level of uncertainty for performance in these subgroups (e.g. With confidence intervals). Where this may suggest additional risk, describe whether mitigation plans are in place to avoid harm to these groups.<br>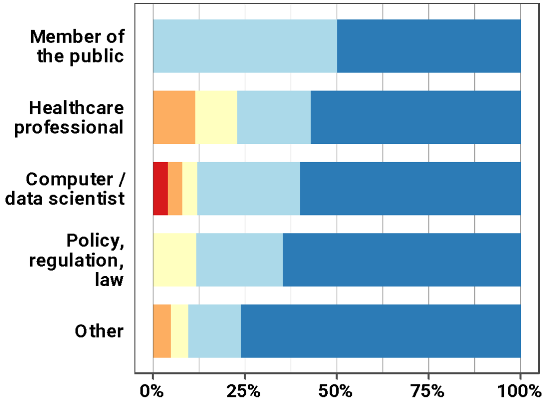<br>*Round 2 item 2.8*<br>6.6% vote to probably/definitely include<br>85.5% vote to probably/definitely include	Should sufficient sample size not be achieved in minority and/or intersectional subgroups, data users should report the level of uncertainty for performance in these subgroups (e.g. With confidence intervals). Where this may suggest additional risk, describe whether mitigation plans are in place to avoid harm to these groups.<br><br>**<u>ACTIONS AT CONSENSUS MEETING</u>**<br>- Discussion amongst attendees about retaining the content of this item, but merging it with other items rather than it being standalone<br>- Proposal to move content to explanatory text of items 2.2d and 2.2e – vote: 88% include, 12% abstain<br>- Re-discussed; re-vote: 100% include<br>- Item removed from final recommendations, but request to report uncertainty of performance and specify if sample size is insufficient moved to other items' explanatory text as requested.

STANDING Together

Survey introduction & consent

STANDING Together - a project to develop STANdards for data Diversity, INclusivity and Generalisability.

Click the play button below to hear this page read out as audio. You'll find buttons like this throughout the questionnaire.

0:00 / 1:04

We would like to invite you to participate in a study to help us create new standards aimed at improving the quality of datasets used in healthcare AI development. **Everyone's welcome to participate, regardless of where you live or your background.**

Our standards' proposed aims are to encourage dataset creators to provide better

documentation, and for users of datasets to choose the best datasets for artificial intelligence (AI) applications in healthcare. We think this can help reduce the risk of biases, and so reduce health inequalities. We hope that the standards we produce will be widely adopted, including by medical professionals, AI developers, medical regulators, and other organisations.

By participating with this study, you'll help us make sure the standards work for you - whether you're a member of the public, a medical professional, a policymaker, a developer, or someone else! The study will take approximately 30 minutes to complete.

Click the blue arrow below to continue

Participant Information Sheet (Delphi Study)

Study Title: Developing STANdards for data Diversity, INclusivity and Generalisability (STANDING together)

0:00 / 6:24

WHAT IS THE PURPOSE OF THIS STUDY?
Artificial Intelligence provides a platform that can make accessible and personalised medicine a reality. Whilst AI has the potential to improve healthcare, it can also perpetuate and even exacerbate existing societal biases leading to further health inequalities. A key consideration is representativeness of the data underpinning AI: if the data is not representative of specific populations, there is a risk that the AI algorithm will underperform in those individuals. Poor data composition and quality in underrepresented groups risks poorer algorithmic performance and perpetuation of societal biases.

To address this, gathering of health data needs to be designed with inclusivity and diversity in mind. We need standards to guide how AI datasets should be composed (‘who’ is represented in the data) and transparency around the data composition (‘how’ they are represented).

This project is about developing standards for health data, to support development of AI algorithms which do not disadvantage minoritised population groups. The standards

arising from this project will be of importance to policy makers, regulators, developers of AI systems, patients and healthcare professionals.

WHO IS ORGANISING AND FUNDING THE RESEARCH?

This project is being conducted by an international team of researchers from multiple research institutes – the full list of co-investigators can be found at www.datadiversity.org. The study is funded by the National Institute of Health Research, NHSx and The Health Foundation as part of an AI and Racial and Ethnic Inequalities in Health and Care award (AI_HI200014).

WHY ARE WE APPROACHING YOU?

We are inviting individuals who have expertise in, or experience of, machine learning, health data science, digital health technologies and health inequalities. Your participation will help us to identify the items that should be incorporated in the standards we are seeking to produce. Additionally, we're inviting patients and other members of the public to participate to ensure that the standards we produce meet the needs of wider society.

WHAT WILL HAPPEN TO ME IF I TAKE PART?

You will be contacted regarding participation in a modified e-Delphi study, which is a two round questionnaire study. There will be a period of three weeks between rounds to allow for response analysis by the study team. A third round may be required in the event of poor consensus in round two. You do not have to complete all the rounds, but we prefer that you do, to preserve the validity of the study findings and minimise bias. Each round of the modified e-Delphi will take no longer than 40 minutes to complete in total.

DO I HAVE TO TAKE PART?

No, your participation is entirely voluntary. If you do decide to take part you will be given a copy of this information sheet and be asked to confirm consent on the first page of the online e-Delphi form.

WHAT WILL HAPPEN IF I NO LONGER WANT TO TAKE PART IN THE STUDY?

You may change your mind at any time (before the start of the study or even after you have commenced the study) for whatever reason without having to justify your decision. Please contact the research team (Dr Xiaoxuan Liu, x.liu.8@bham.ac.uk) and they will remove your confidential information from the study database. You may additionally ask for the removal of data you have provided up until the point of withdrawal, in which case we will remove any data that is identifiably linked to you. However, please be advised that data will be aggregated and anonymised five days after each round of the Delphi study has closed, so if you withdraw after this date, we will no longer be able to remove this data as we will not be able to identify that it was specifically collected from you.

WHAT ARE THE POSSIBLE BENEFITS OF TAKING PART?

There will be no direct benefits for you if you decide to take part. However, there will be wider benefits anticipated for marginalised or disadvantaged groups, who may not have been previously represented in the development of AI in healthcare. Thus ensuring that minority groups can derive the benefit from this technology that other healthcare groups currently already experience.

WILL MY TAKING PART IN THIS STUDY BE KEPT CONFIDENTIAL?

All information about you which is connected with the research study will be kept strictly confidential. Only the research team will have access to any data generated from this study. The data that we will collect from you include the following: name, contact details (email), age, gender, ethnicity, professional role, organisation you work for and geographical location. When we use your information for research, we rely on Article 6(1)e ("processing is necessary for the performance of a task carried out in the public interest") and Article 9(2)j ("processing is necessary for archiving purposes in the public interest, scientific or historical research purposes") of the General Data Protection Regulation (GDPR) in combination with Schedule 1, Part 1, Art 4 Data Protection Act (DPA) 2018. For more information on how we manage your data and what are your rights, our privacy notice can be accessed at https://www.birmingham.ac.uk/privacy/index.aspx

Electronic data will be stored on University of Birmingham encrypted computers that are password-protected, whilst any data collected on paper, such as paper consent forms and any paper correspondence will be stored in locked filing cabinets in the researcher's office. Data will be kept for 10 years and after this retention period has

finished, the study data will be destroyed in line with University of Birmingham's standard operating procedures. Any information used in publications or reports will be anonymised so that your identity cannot be known.

WHO HAS REVIEWED THIS STUDY?
This research study has been reviewed and approved by the University of Birmingham ethics committee (ERN_21-1831).

If you have concerns about any aspect of this study, or if you wish to withdraw, please contact Dr Xiaoxuan Liu (x.liu.8@bham.ac.uk) or contact@datadiversity.org

I confirm that I have read the participant information and I am willing to participate in this study

If you select 'no' below you'll be taken to the end of the survey and no data will be recorded about you

- ○ Yes
- ○ No

I consent to information I enter into this form being stored and processed by the STANDING Together research team in accordance with the privacy notice above

If you select 'no' below you'll be taken to the end of the survey and no data will be recorded about you

- ○ Yes
- ○ No

Briefing

Over the next few pages you'll have the opportunity to read and vote upon a number of proposed statements which will make up our final set of standards.

0:00 / 1:24

Throughout this survey certain key words are underlined, and in blue text. *These can*

be hovered over to learn more about what they mean. If you are using a mobile device you can tap the word instead.

These statements have been produced by the STANDING Together research team following a systematic review of existing dataset standards, and collecting the views of a wide range of experts in this field.

The proposed standards are split into two parts:

1) Documentation standards - to encourage transparency and clarity about a dataset for those using it for research or development of healthcare AI systems.

2) Process standards - to provide guiding principles on appropriateness of the data in particular circumstances

In each of these two parts there are several pages, and each page has several proposed statements.

For each of the statements you'll be able to vote on whether the item should be included or excluded. You'll also be able to write any comments you like about the item. This can be in relation to the content, to the way it's written, or anything else. We will act on your comments, so this is a great way to improve the items if you think this is needed.

Click here to download a copy of all of the draft delphi items in a single document. This might be helpful to see how they all fit together. NB - the document may open in a new tab in your web browser.

Below is a short video which explains a bit more about how the delphi study will work. When you're ready to continue, click the 'Next Page' button below to begin the survey.

We recommend you complete this survey using a computer, but it should also work on mobile devices.

We also recommend you complete the survey in one sitting, but if you need to pause and come back your responses should be saved as long as you use the

same device as before. This will only work if you have cookies enabled on your device and you're not using a private browsing mode.

Documentation: Dataset summary

1 - Dataset Documentation Standards

0:00 / 1:25

The primary purpose of the Dataset Documentation Standards is to promote minimum reporting requirements for the aspects of a dataset which may introduce bias. The goal is to provide sufficient information so that users of the data, as well as third party evaluators and auditors (such as regulators and policymakers) can appraise the datasets and make an informed decision on the risk of bias when applied to a specific use-case.

The Dataset Documentation Standards are primarily for Data Curators, but it will also be useful for the Data Users. For the purposes of these standards, Data Curators are defined as anyone involved in making a dataset available for use - this will include those who generate, collect or process data, or make decisions about its structure and/or availability.

The intention of Dataset Documentation Standards are not to be prescriptive about the contents of datasets, but to encourage transparent description, so that others may make the judgement for themselves on issues such as bias. These are generic considerations and therefore all items should be considered applicable to all datasets.

Please indicate whether we should include or exclude each of the below delphi items. There is a space to write comments or suggest improvements below each item.

DATASET SUMMARY

1.1 Dataset summary

Provide a brief summary of the dataset: including a description of the contents, source and purpose of the dataset.

0:00 / 0:59

1 - Definitely exclude

○

2 - Probably exclude

○

3 - Unsure

○

4 - Probably include

○

5 - Definitely include

○

Any comments on item 1.1?

1.2 Dataset identity and access

Provide essential information regarding accessibility, date of release, version, dataset size, licensing and ownership.

0:00 / 1:07

1 - Definitely exclude

2 - Probably exclude

3 - Unsure

4 - Probably include

5 - Definitely include

Any comments on item 1.2?

1.3 Motivations for dataset creation and intended purpose(s)

Describe the motivations behind the creation of this dataset, including: the purpose for dataset creation, the intended benefit, any purposes for which dataset use should be avoided, who created the dataset and who funded it.

0:00 / 0:47

1 - Definitely exclude

2 - Probably exclude

3 - Unsure

4 - Probably include

5 - Definitely include

Any comments on item 1.3?

1.4 Dataset curation team

Describe how the diversity of those represented within the dataset are reflected in the curation team, to ensure assumptions and preconceptions are considered. This may involve attributes of the dataset curators themselves, as well as governing and consultation groups (e.g. advisory boards, patient and public involvement and engagement groups).

0:00 / 1:11

1 - Definitely exclude

2 - Probably exclude

3 - Unsure

4 - Probably include

5 - Definitely include

Any comments on item 1.4?

1.5 Relation to the original data source, including sampling strategy

Describe the original source of data and the data sampled, including an explanation of sampling strategies and their rationale. Describe the reason for generating the original data (e.g., patient records to provide clinical care, clinical trial, biobank) and what individuals were expecting to happen to their data (e.g., administrative action, participant in a research study). If the dataset has been compiled from multiple data sources, describe how datasets were selected, and how decisions were made during data aggregation, particularly in the case of grouping populations and modification of demographic coding.

0:00 / 1:23

1 - Definitely exclude

2 - Probably exclude

3 - Unsure

4 - Probably include

5 - Definitely include

Any comments on item 1.5?

1.6 Data shifts

For longitudinal datasets or datasets with versions, describe any changes over time relating to the population, medical practice, or how data were collected, which may contribute to data shifts.

0:00 / 1:02

1 - Definitely exclude

○

2 - Probably exclude

○

3 - Unsure

○

4 - Probably include

○

5 - Definitely include

○

Any comments on item 1.6?

1.7 Limitations of the dataset

Identify known or suspected sources of bias, error or other factors that affect the dataset as a whole, which may impact its generalisability or applicability for other use.

0:00 / 0:53

1 - Definitely exclude

○

2 - Probably exclude

○

3 - Unsure

○

4 - Probably include

○

5 - Definitely include

○

Any comments on item 1.7?

Documentation: Recording of attributes of individuals

We recommend you complete the survey in one sitting, but if you need to pause and come back your responses should be saved as long as you use the same device as before. This will only work if you have cookies enabled on your device and you're not using a private browsing mode.

RECORDING OF ATTRIBUTES OF INDIVIDUALS

1.8 Composition of populations

Summarise the relevant populations present in the dataset. Defining 'relevant populations' should be contextualised according to this dataset's specific use cases and contexts, where possible. Certain attributes (including age, gender identity, sex, race, ethnicity, socioeconomic status) should always be documented, due to known associations with health outcomes and interactions with wider social factors. If data on these particular attributes are missing this should be stated. Highlight known missing population groups within the dataset and the reason for their missingness.

0:00 / 0:00

1 - Definitely exclude

2 - Probably exclude

3 - Unsure

4 - Probably include

5 - Definitely include

Any comments on item 1.8?

1.9 Recording of attributes of individuals

Describe how attributes were collected (self-reported, imputed, linked through other datasets), whether this information is available at the individual or aggregate level, and whether data missingness is different across groups. Explain whether attributes have been coded, condensed or modified, stating how and why this was done.

0:00 / 0:00

1 - Definitely exclude

○

2 - Probably exclude

○

3 - Unsure

○

4 - Probably include

○

5 - Definitely include

○

Any comments on item 1.9?

1.10 Groups experiencing vulnerabilities

Highlight the presence of any vulnerable population groups in this dataset, with consideration of vulnerabilities that are both universal (e.g., children, people with severe disabilities, displaced persons) and those that are specific to the site of data collection (e.g, marginalised religious or caste groups, sexual orientation and gender identity groups).

0:00 / 0:00

1 - Definitely exclude

○

2 - Probably exclude

○

3 - Unsure

○

4 - Probably include

○

5 - Definitely include

○

Any comments on item 1.10?

Documentation: Sources of bias

We recommend you complete the survey in one sitting, but if you need to pause and come back your responses should be saved as long as you use the same device as before. This will only work if you have cookies enabled on your device and you're not using a private browsing mode.

SOURCES OF BIAS

1.11 Modifications made to the data

Describe data items which were modified from the raw source and provide the rationale for doing so and the method used. For example, to anonymise the data, to correct for imbalance, to correct error, biases, mapping to existing data

standards (e.g. OMOP, FHIR, DICOM).

0:00 / 0:00

1 - Definitely exclude

2 - Probably exclude

3 - Unsure

4 - Probably include

5 - Definitely include

Any comments on item 1.11?

1.12 Missing data

Describe the proportion, nature and causes of missing data, particularly if there are systematic differences across relevant population groups. Describe how missing data has been identified and handled (e.g. imputation, correction).

0:00 / 0:00

1 - Definitely exclude

2 - Probably exclude

3 - Unsure

4 - Probably include

5 - Definitely include

Any comments on item 1.12?

1.13 Errors in the data

Describe how errors can be/have been identified in the data and how they have been handled (e.g. have they been removed, modified, corrected or left in the dataset). Provide an estimation of the proportion of errors that are present and whether they are more prevalent in some population groups than others. Provide possible reasons for any systematic differences in error rates across population groups within the dataset.

0:00 / 0:00

1 - Definitely exclude

2 - Probably exclude

3 - Unsure

4 - Probably include

5 - Definitely include

Any comments on item 1.13?

1.14 Known or potential bias in data generation

Describe how bias may be introduced by the acquisition and processing of data within the dataset, for example from the use of devices, sensors and software. Highlight any known or potential differences in data acquired across different population groups, or any uncertainty in performance within population groups.

0:00 / 0:00

1 - Definitely exclude

2 - Probably exclude

3 - Unsure

○

4 - Probably include

○

5 - Definitely include

○

Any comments on item 1.14?

1.15 Known or potential bias in data collection

Identify areas where bias may have been introduced into the data collection process. For example, only collecting data from one geographical area, only using questionnaires in English.

0:00 / 0:00

1 - Definitely exclude

○

2 - Probably exclude

○

3 - Unsure

○

4 - Probably include

○

5 - Definitely include

○

Any comments on item 1.15?

1.16 Known or potential bias in data labels

Provide a description of any data labels, including who decided what labels to include, what they were called, and how they were generated. Highlight labels that are at high risk of bias. For example, where label generation was at the discretion of individuals, where known biases in labelling behaviour has been evidenced previously, or in the use of proxy variables (e.g., healthcare costs as a proxy of healthcare needs).

0:00 / 0:00

1 - Definitely exclude

○

2 - Probably exclude

○

3 - Unsure

○

4 - Probably include

○

5 - Definitely include

○

Any comments on item 1.16?

Documentation: Ethics and data governance

We recommend you complete the survey in one sitting, but if you need to pause and come back your responses should be saved as long as you use the same device as before. This will only work if you have cookies enabled on your device and you're not using a private browsing mode.

ETHICS AND DATA GOVERNANCE

1.17 Ethics and data governance

Describe the consent process for inclusion in the dataset, measures taken to protect identities of the individuals, permissions and governance of the dataset, and provide references to institutional review board/ethical committee review. Reference any standards (e.g. ISO, FAIR) which have been adhered to.

0:00 / 0:00

1 - Definitely exclude

○

2 - Probably exclude

○

3 - Unsure

○

4 - Probably include

○

5 - Definitely include

○

Any comments on item 1.17?

1.18 Patient and public involvement

Describe the role of advisory boards and patient and public involvement and engagement groups in the dataset curation. Provide information on efforts to share data and findings with those who contributed to the dataset and any feedback that was gathered from participants that is relevant to data interpretation.

0:00 / 0:00

1 - Definitely exclude

2 - Probably exclude

3 - Unsure

4 - Probably include

5 - Definitely include

Any comments on item 1.18?

1.19 Bias and impact assessments

If a formal assessment of bias, fairness or societal impact has been previously conducted on the dataset, please provide the assessment and results. This may include algorithmic impact assessments (AIAs), data protection impact assessments (DPIAs), equality impact assessments, documentation tools, risk of bias assessments or automated toolkits.

0:00 / 0:00

1 - Definitely exclude

2 - Probably exclude

3 - Unsure

4 - Probably include

5 - Definitely include

Any comments on item 1.19?

Process items - part 1 of 2

We recommend you complete the survey in one sitting, but if you need to pause and come back your responses should be saved as long as you use the same device as before. This will only work if you have cookies enabled on your device and you're not using a private browsing mode.

2 - Dataset Process Standards

0:00 / 0:00

The primary purpose of ***Dataset Process Standards*** is to promote best practice for how datasets should be used throughout the AI Health Technology lifecycle, and

promote documentation of how risks to relevant subgroups have been identified and mitigated.

The Dataset Process Standards are primarily for Data Users, as they require context and should be considered with a specific use-case in mind. The concept of **contextualised subgroups of interest** is introduced: subgroups with shared attributes, identified as being relevant and important for the use case, and where they are known to have worse health outcomes or are subject to other systems driving health inequity related to the use case.

The items are written so that they can be operationalisable within the context of existing medical device regulation, leaning upon existing requirements such as intended use statements, evidentiary requirements (literature review, clinical evaluation plan) and post-market surveillance and post-market clinical follow-up. Where certain items may not be relevant, this is specifically called out in the text. Unlike the documentation requirements above, a 'NA' or 'not done' response is not considered acceptable for any of these (unless, as previously stated, a 'not applicable' option is specifically called out).

Please indicate whether we should include or exclude each of the below delphi items. There is a space to write comments or suggest improvements below each item.

PROVIDE SUFFICIENT INFORMATION ABOUT DATASET(S) TO ALLOW TRACEABILITY AND AUDITABILITY

2.1

Full documentation of datasets used in the lifecycle of AI health technologies should be provided to enable audit against these standards.

0:00 / 0:00

1 - Definitely exclude

○

2 - Probably exclude

○

3 - Unsure

○

4 - Probably include

○

5 - Definitely include

○

Any comments on item 2.1?

EVALUATE IN THE CONTEXT OF RELEVANT SUBGROUPS

2.2

Data Users should identify contextualised subgroups of interest in advance: these are subgroups with shared attributes, identified as being relevant and important for the use case, and where they are known to have worse health outcomes or are subject to other systems driving health inequity related to the use case.

0:00 / 0:00

1 - Definitely exclude

2 - Probably exclude

3 - Unsure

4 - Probably include

5 - Definitely include

Any comments on item 2.2?

2.3

Data Users should identify and document any evidence uncovered during literature review highlighting discrepant performance across any subgroups in similar devices.

0:00 / 0:00

1 - Definitely exclude

2 - Probably exclude

3 - Unsure

4 - Probably include

5 - Definitely include

Any comments on item 2.3?

2.4

Having identified contextualised subgroups of interest, Data Users should report evaluation results of the AI health technology within those subgroups, in addition to aggregate results of the evaluation.

0:00 / 0:00

1 - Definitely exclude

2 - Probably exclude

3 - Unsure

4 - Probably include

5 - Definitely include

Any comments on item 2.4?

2.5

Data Users should also report evaluation results across certain attributes (including age, gender, sex, race, ethnicity, socioeconomic status), due to known associations with health outcomes and interactions with wider social factors. This may not always be possible or appropriate, in which case the reasons for not doing so should be documented.

0:00 / 0:00

1 - Definitely exclude

2 - Probably exclude

3 - Unsure

4 - Probably include

○

5 - Definitely include

○

Any comments on item 2.5?

USE APPROPRIATE DATASETS TO SUPPORT THE INTENDED USE

2.6

The intended use population of the AI health technology should be adequately represented in the training and test datasets for an AI Health Technology. The contextualised subgroups of interest should also be included where possible, and if not, explicitly stated. Areas of under-representation should be identified and transparently reported.

0:00 / 0:00

1 - Definitely exclude

○

2 - Probably exclude

○

3 - Unsure

○

4 - Probably include

○

5 - Definitely include

○

Any comments on item 2.6?

Process items - part 2 of 2

We recommend you complete the survey in one sitting, but if you need to pause and come back your responses should be saved as long as you use the same device as before. This will only work if you have cookies enabled on your device and you're not using a private browsing mode.

ACKNOWLEDGE KNOWN BIASES AND LIMITATIONS OF DATASET(S) AND ANY IMPLICATIONS ON THE INTENDED USE OF THE AI HEALTH TECHNOLOGY

2.7

Data Users should report limitations of the dataset and the implications on the AI health technology. Data Users should investigate whether limitations are systematically different across relevant population subgroups, including those categorised as ‘unknown’ or ‘other’, and report differences which could result in worse performance on the AI Health Technology across groups.

0:00 / 0:00

1 - Definitely exclude

○

2 - Probably exclude

○

3 - Unsure

○

4 - Probably include

○

5 - Definitely include

○

Any comments on item 2.7?

2.8

State any intended purposes of the dataset, and how this differs from the intended use of the AI health technology. State implications of any discordance and provide justification regarding the suitability of the dataset, including assumptions made and aspects of the dataset which are not directly applicable.

0:00 / 0:00

1 - Definitely exclude

○

2 - Probably exclude

○

3 - Unsure

○

4 - Probably include

○

5 - Definitely include

○

Any comments on item 2.8?

2.9

In the case of minority and intersectional population groups, achieving sufficient sample size may not be possible. Data Users should report the level of uncertainty for performance in these subgroups (e.g. with confidence intervals). Where this may confer additional risk, mitigation plans should be in place to avoid harm to these groups.

0:00 / 0:00

1 - Definitely exclude

○

2 - Probably exclude

○

3 - Unsure

○

4 - Probably include

○

5 - Definitely include

○

Any comments on item 2.9?

2.10

Data Users should review pre-existing assessments of the datasets (e.g. algorithmic impact assessments, equality impact assessments, data protection impact assessments, datasheets, healthsheets) and report how the findings may translate to harm for subgroups within the intended use population.

0:00 / 0:00

1 - Definitely exclude

○

2 - Probably exclude

○

3 - Unsure

○

4 - Probably include

○

5 - Definitely include

○

Any comments on item 2.10?

ADDRESS UNCERTAINTIES AND RISKS WITH MITIGATION PLANS

2.11

Where Data Users have identified uncertainty or potentially variable performance in subgroups, this should be identified and reported as a risk. If the Data User is a manufacturer of an AI health technology, risks must be monitored as part of the post market clinical follow-up and post market surveillance plans.

0:00 / 0:00

1 - Definitely exclude

2 - Probably exclude

3 - Unsure

4 - Probably include

5 - Definitely include

Any comments on item 2.11?

Survey ending

We will amend the standard items contained in this survey based on your feedback.

We will then repeat this survey later this year - if you're willing to help us in the second round please provide an email address below.

How did you find out about our delphi study?

- ○ I received an email inviting me to participate
- ○ I clicked the link in the Nature Medicine paper
- ○ Social media
- ○ I was told about the study by someone I know (including work colleagues)
- ○ Via the website: www.datadiversity.org
- ○ Other (please state below)

What is your email address?

We will use this only to contact you regarding completing Delphi round 2

Please repeat your email address

Which of the following statements best describe you?

You can select more than one option if needed

- ☐ I'm a healthcare professional
- ☐ I'm a member of the public
- ☐ I'm a computer scientist / data scientist
- ☐ I work in policy, regulation, law, politics, or related fields
- ☐ I'm an academic researcher
- ☐ I work in social science
- ☐ Other (please state below)

Thank you for completing our survey - just a couple of final questions

These final questions are **voluntary**. If you'd rather not answer any or all of them you can scroll down and submit the survey.

Our standards aim to protect minoritised groups in society from potential harms associated with medical AI. To ensure we meet this aim, it's important we hear the voices of those who are minoritised. Your responses to these next questions will help us understand what types of people have responded to our survey so we can make sure our standards represent the views of as many people in society as possible.

We will not share your responses with anyone else.

We will not store your responses to these questions with your previous responses in this survey - they will be aggregated and individual responses will be redacted. For a reminder of how we will use your personal information, please click here.

What is your ethnicity?

We've used the UK Office for National Statistics categories here. We recognise these categories are not inclusive for all geographies and communities, so if you'd prefer to self identify there are options to write a free-text response.

- ○ I'd prefer not to answer this question

White

- English / Welsh / Scottish / Northern Irish / British
- Irish
- Gypsy or Irish Traveller
- Any other White background (please describe in the self identification box below)

Mixed / multiple ethnic groups

- White and Black Caribbean
- White and Black African
- White and Asian
- Any other Mixed / Multiple ethnic background (please describe in the self identification box below)

Asian / Asian British

- Indian
- Pakistani
- Bangladeshi
- Chinese
- Any other Asian background (please describe in the self identification box below)

Black / African / Caribbean / Black British

- African
- Caribbean
- Any other Black / African / Caribbean background (please describe in the self identification box below)

Other ethnic group

- Arab
- Any other ethnic group (please describe in the self identification box below)

What is your race?

For some people race and ethnicity are the same thing, but for others they are different constructs. Please interpret this question in your own context - we've not provided categories to allow maximal self expression. You can leave this blank if you'd prefer.

What is your age?

We've used ranges here to protect your identity

- I'd prefer not to answer this question
- 0-17 years
- 18-30 years
- 30-60 years
- 60+ years

In which country do you currently reside?

What is your sex (as assigned at birth)?

- I'd prefer not to answer this question
- Female
- Male
- Intersex

- Other

Is your gender identity the same as the sex you were assigned at birth?

- I'd prefer not to answer this question
- Yes
- No

How do you describe your sexual orientation?

- I'd prefer not to answer this question
- Homosexual
- Bisexual
- Pansexual
- Asexual
- Heterosexual
- I'd prefer to self identify (please give response below)

If you have any final comments about any of the standard items, the way we've run this study, or anything else, please write them below

Powered by Qualtrics

STANDING Together

Survey introduction & consent

STANDING Together - a project to develop STANdards for data Diversity, INclusivity and Generalisability.

Welcome to round 2 of the delphi study for the STANDING Together project

Throughout this survey certain key words are underlined, and in blue text. These can be hovered over to learn more about what they mean. If you are using a mobile device you can tap the word instead.

You can click the play button below to hear this page read out as audio. You'll find buttons like this throughout the questionnaire.

0:00 / 1:33

Thank you for completing the first round of the STANDING Together delphi study back in October! Based on your votes and comments, we've suggested some changes to our draft standards.

As a reminder, these are aimed at improving the quality of datasets used in healthcare artificial intelligence (AI) development. Our standards' proposed aims are to encourage dataset creators to provide better documentation, and for users of datasets to choose the best datasets for AI applications in healthcare. We think this can help reduce the risk of AI biases, and so reduce the risk of health inequalities. We hope that the standards we produce will be widely adopted, including by medical professionals, AI developers, medical regulators, and other organisations.

By participating with round 2 of this study, you'll help us make sure the standards work for you - whether you're a member of the public, a medical professional, a policymaker, a developer, or someone else! The study will take approximately 30 minutes to complete.

Click the blue arrow below the video to continue >>>

Participant Information

Study Title: Developing STANdards for data Diversity, INclusivity and Generalisability (STANDING together)

You will already have read and reviewed the participant information sheet when you completed round 1 of the delphi study - none of this information has changed. We do know that it can be hard to remember some of this information - if you'd like to remind yourself about the study (including how we will use any data you provide in this form) please click here to open a copy of the information sheet in your web browser.

Alternatively, you can click the play button below to listen to a recorded version of the information sheet.

0:00 / 6:24

If you have questions or concerns about any aspect of this study, or if you wish to withdraw, please contact Dr Xiaoxuan Liu - contact@datadiversity.org

I confirm that I participated in round 1 of this delphi study

- [ ] Yes
- [ ] No

I confirm that I am willing to participate in round 2 of this study

If you select 'no' below you'll be taken to the end of the survey and no data will be recorded about you

- [ ] Yes
- [ ] No

I consent to information I enter into this form being stored and processed by the STANDING Together research team

To be reminded of how we will use your data:
Click here to open a copy of the participant information sheet in your browser
Click here to view the University of Birmingham data protection policies

If you select 'no' below you'll be taken to the end of the survey and no data will be recorded about you

- [ ] Yes
- [ ] No

Briefing

Thank you for your votes and comments on the items in round 1. Below we set out how they have been updated.

0:00 / 1:59

In round 1 of this delphi study we asked you to vote and comment on two sets of standards:

1) Documentation standards - to encourage transparency and clarity about the contents of a dataset for those using it - particularly for research or development of healthcare AI systems.

2) Process standards - to allow those using a dataset to judge if it meets their aims

(given their particular circumstances).

How we've acted on your feedback

We set a 'threshold' of 75% for items to be excluded. This means that if 75% or more respondents voted to 'definitely exclude' or 'probably exclude' any particular item, it would be omitted both from round 2 of the delphi study, and from the final set of standards.

> *No items received 75% or greater votes to be excluded in this way, so all items were carried forwards to round 2 of the delphi study.*

We have read and acted upon every single comment posted in response to the items in round 1 of the delphi study. Some delphi items have changed quite substantially, and others minimally or not at all. We have added one new item, merged two items into one, and rearranged the order of others to improve the 'flow' of the items overall - all of this in response to your feedback.

Some of the item numbers have changed between rounds 1 and 2. This is because of items being moved, added, removed or combined.

What happens in round 2 of the delphi study?

Similar to in round 1, you'll be able to vote and comment on each delphi item. We will present the updated item first, then the original item underneath in italic text. **You should vote and comment on the new item**, not the item as it was in round 1 of the delphi study.

Will there be a round 3?

The same threshold will apply to items in round 2 as it did in round 1. We only plan to open round 3 if one or more items are to be omitted, or if comments indicate substantial alteration are needed to items.

0:00 / 0:48

Click here to download a copy of all of the draft delphi items in a single document, comparing the items between rounds 1 and 2. This might be helpful to see how they all

fit together. NB - the document may open in a new tab in your web browser.

Below is a short video which explains a bit more about how round 2 of the delphi study will work. When you're ready to continue, click the 'Next Page' button below to begin the survey.

We recommend you complete this survey using a computer, but it should also work on mobile devices.

We also recommend you complete the survey in one sitting, but if you need to pause and come back your responses should be saved as long as you use the same device as before. This will only work if you have cookies enabled on your device and you're not using a private browsing mode.

Documentation: Dataset summary

1 - Dataset Documentation Standards

0:00 / 1:09

The primary purpose of the Dataset Documentation Standards is to promote reporting of aspects of a dataset which may introduce bias. The goal is to provide sufficient

information so that users of the data, as well as third party evaluators and auditors (such as regulators and policymakers) can appraise the datasets and make an informed decision on the risk of bias when applied to a specific use-case.

The Dataset Documentation Standards are primarily for Data Curators, but it will also be useful for the Data Users. For the purposes of these standards, Data Curators are defined as anyone involved in making a dataset available for use - this will include those who generate, collect or process data, or make decisions about its structure and/or availability.

The intention of Dataset Documentation Standards are not to be prescriptive about the contents of datasets, but to encourage transparent description, so that others may make the judgement for themselves on issues such as bias. These are generic considerations and therefore all items should be considered applicable to all datasets, so where certain information is not available or cannot be reported this should be explicitly stated.

DATASET SUMMARY

1.1 Dataset summary

Dataset curators should provide documentation for datasets they produce. This documentation should include a description of the contents, source and purpose of the dataset, and should be written in accessible language. The summary should help data users assess whether the dataset meets their needs.

0:00 / 0:29

How did this item perform in round 1?

Original wording: Dataset summary. *Provide a brief summary of the dataset: including a description of the contents, source and purpose of the dataset.*

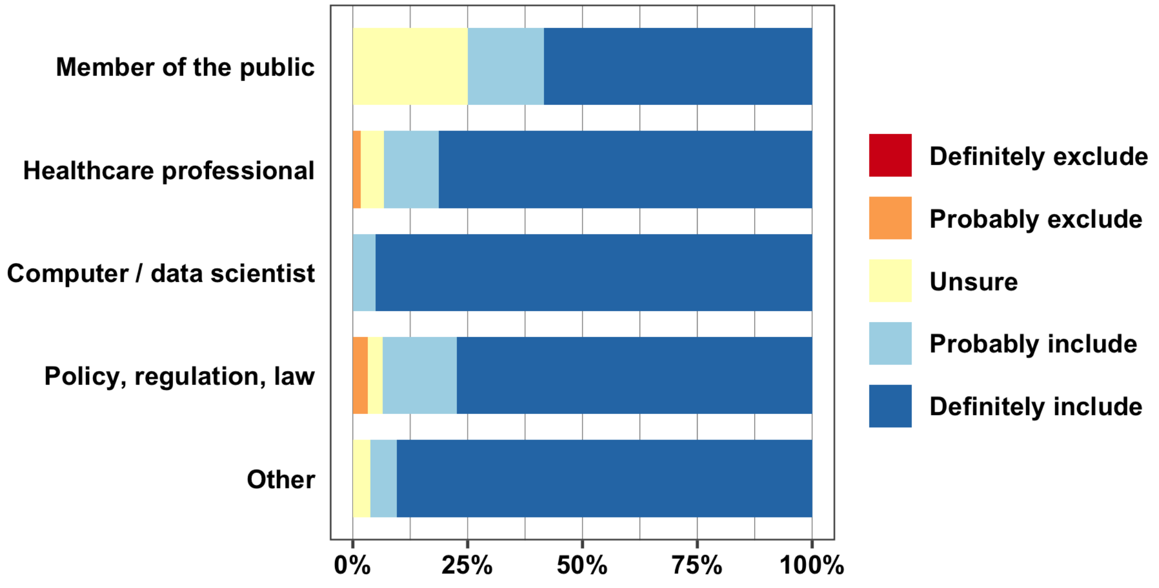

Round 1 item 1.1 (original version)

Please vote on the ***new*** *version of item 1.1 (highlighted in blue above).*

1 - Definitely exclude ○

2 - Probably exclude ○

3 - Unsure ○

4 - Probably include ○

5 - Definitely include ○

Any comments on item 1.1?

1.2 Dataset identity and access

Dataset documentation should include: dataset name, accessibility, date of release, version, dataset size, licensing arrangements and details of any data custodian(s). Where possible this documentation should adhere to FAIR principles.

0:00 / 0:29

How did this item perform in round 1?

Original wording: Dataset identity and access. *Provide essential information regarding accessibility, date of release, version, dataset size, licensing and ownership.*

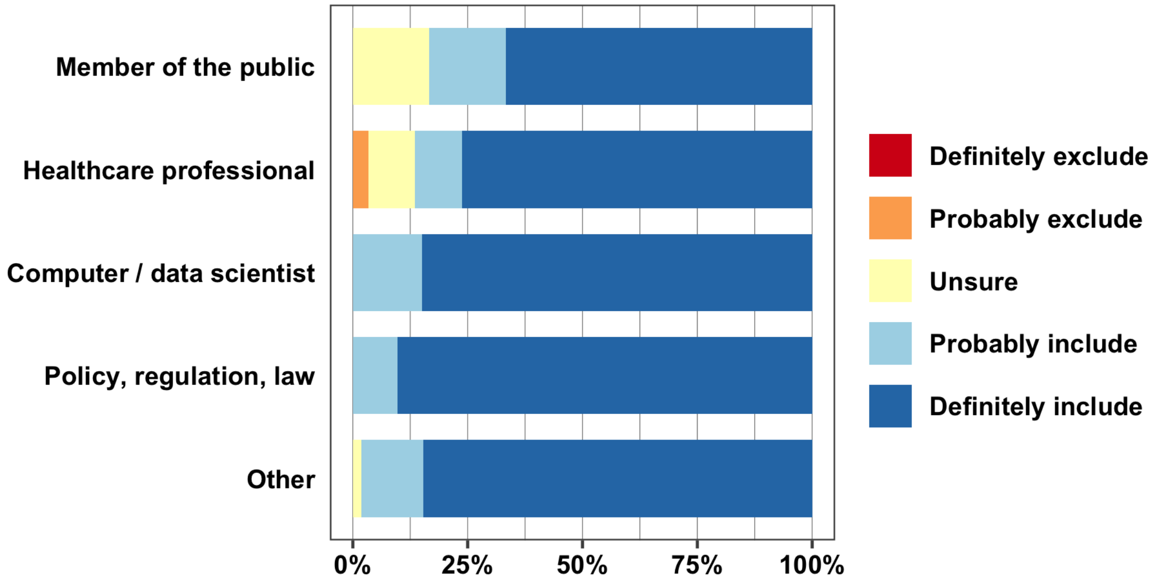

Round 1 item 1.2 (original version)

Please vote on the ***new*** *version of item 1.2 (highlighted in blue above).*

1 - Definitely exclude

○

2 - Probably exclude

○

3 - Unsure

○

4 - Probably include

○

5 - Definitely include

○

Any comments on item 1.2?

1.3 Motivations for dataset creation and intended purpose(s)

Dataset documentation should include the reasons why this dataset was created, including any intended benefit(s), any purposes for which dataset use should be avoided, who created the dataset and who funded it.

0:00 / 0:32

How did this item perform in round 1?

Original wording: Motivations for dataset creation and intended purpose(s). *Describe the motivations behind the creation of this dataset, including: the purpose for dataset creation, the intended benefit, any purposes for which dataset use should be avoided, who created the dataset and who funded it.*

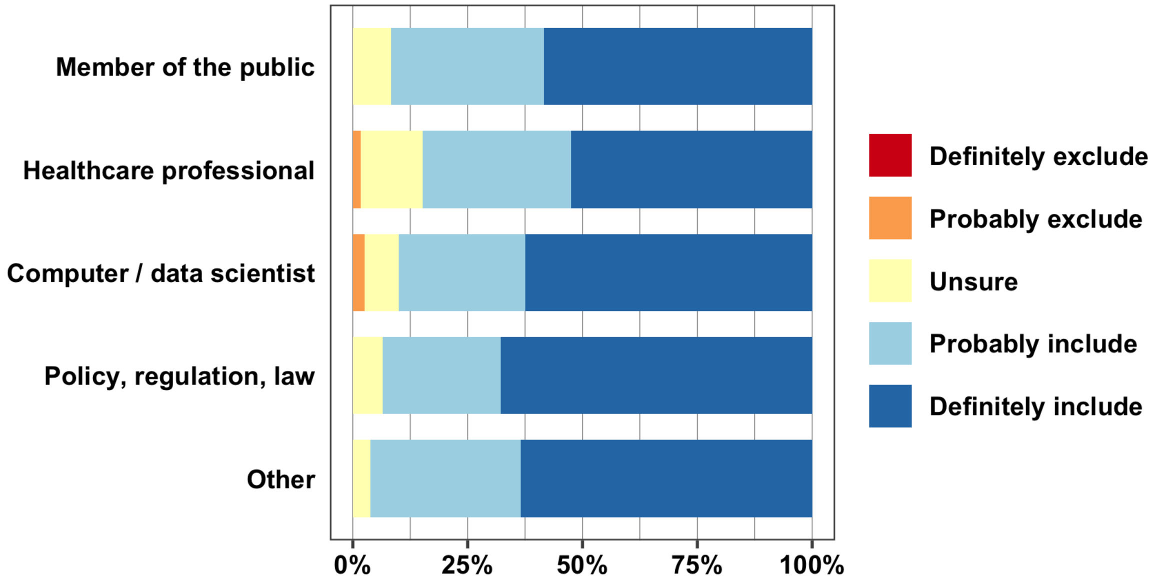

Round 1 item 1.3 (original version)

*Please vote on the **new** version of item 1.3 (highlighted in blue above).*

1 - Definitely exclude

2 - Probably exclude

3 - Unsure

4 - Probably include

5 - Definitely include

Any comments on item 1.3?

1.4 Assumptions and preconceptions of the dataset curation team

Dataset documentation should describe how the curation team has considered the impact of their prior assumptions and preconceptions on biases in the dataset. This may include reflecting on the experiences of the dataset curators themselves, as well as any governing and consultation groups (e.g. advisory boards, patient and public involvement and engagement groups).

0:00 / 0:47

How did this item perform in round 1?

Original wording: Dataset curation team. *Describe how the diversity of those represented within the dataset are reflected in the curation team, to ensure assumptions and preconceptions are considered. This may involve attributes of the dataset curators themselves, as well as governing and consultation groups (e.g. advisory boards, patient and public involvement and engagement groups).*

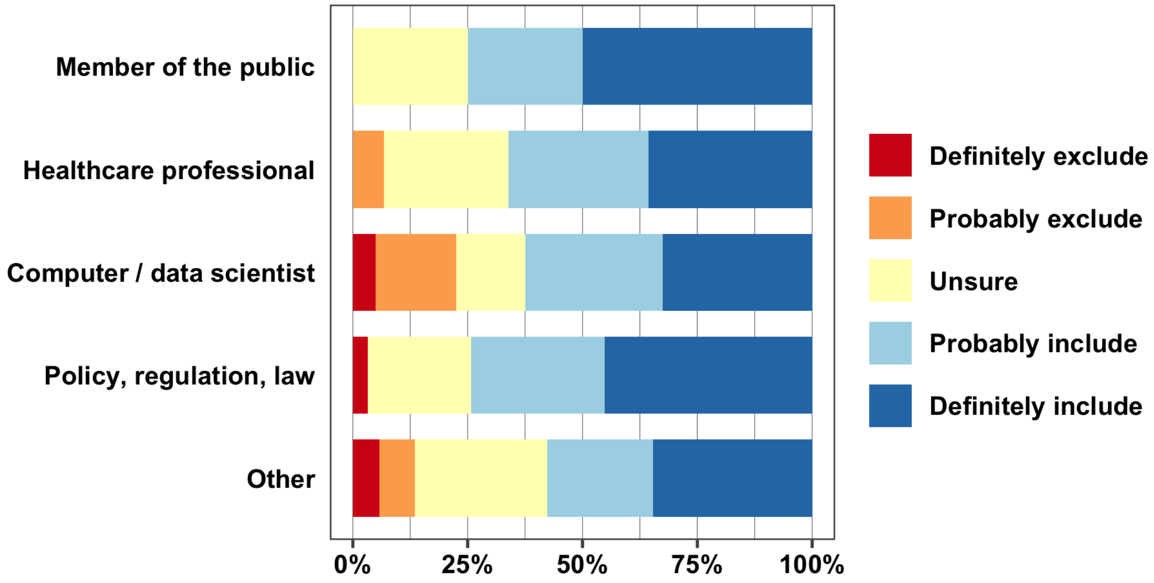

Round 1 item 1.4 (original version)

*Please vote on the **new** version of item 1.4 (highlighted in blue above).*

1 - Definitely exclude

○

2 - Probably exclude

○

3 - Unsure

○

4 - Probably include

○

5 - Definitely include

○

Any comments on item 1.4?

1.5 Origin and purpose of source data

Dataset documentation should describe the original source of data, including the reason for generating the original data (e.g., patient records to provide clinical care, clinical trial, biobank) and what individuals were expecting to happen to their data (e.g., administrative action, participant in a research study).

0:00 / 1:06

How did this item perform in round 1?

Original wording: Relation to the original data source, including sampling strategy. *Describe the original source of data and the data sampled, including an explanation of sampling strategies and their rationale. Describe the reason for generating the original data (e.g., patient records to provide clinical care, clinical trial,*

biobank) and what individuals were expecting to happen to their data (e.g., administrative action, participant in a research study). If the dataset has been compiled from multiple data sources, describe how datasets were selected, and how decisions were made during data aggregation, particularly in the case of grouping populations and modification of demographic coding.

[Removed text added below to create a new item 1.6 for round 2]

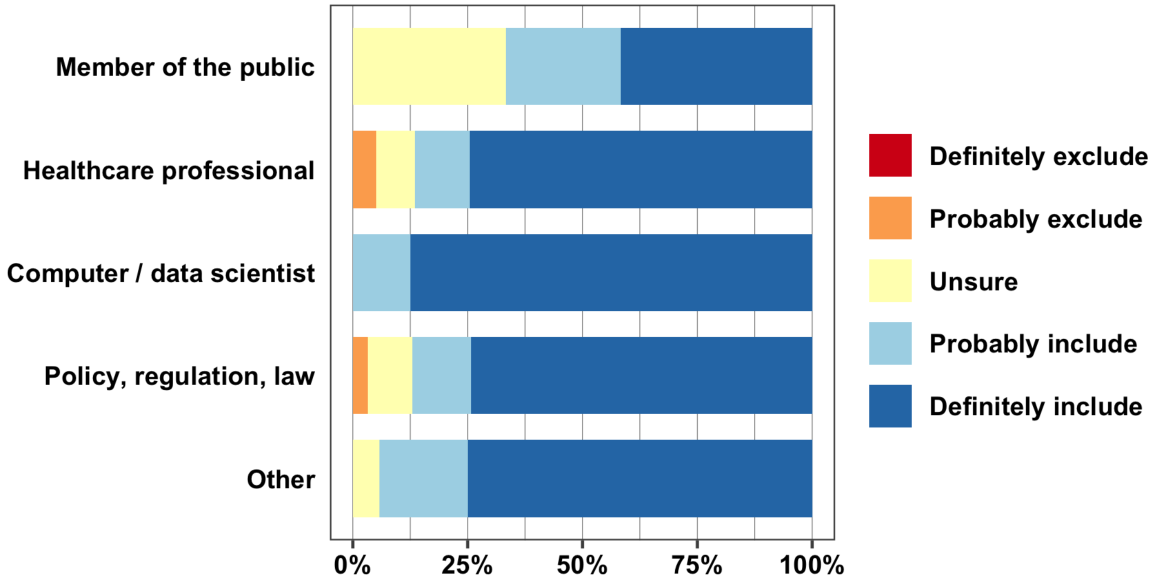

Round 1 item 1.5 (original version)

*Please vote on the **new** version of item 1.5 (highlighted in blue above).*

1 - Definitely exclude

2 - Probably exclude

3 - Unsure

4 - Probably include

5 - Definitely include

Any comments on item 1.5?

1.6 Data sampling, and aggregation from multiple data sources

Dataset documentation should describe how data were sampled, including an explanation of sampling strategies and their rationale. If the dataset has been compiled from multiple data sources, dataset documentation should describe how datasets were selected, and how decisions were made during data aggregation, particularly in the case of grouping populations and modification of demographic coding.

0:00 / 0:31

New item for round 2 (split apart from old item 1.5)

1 - Definitely exclude

2 - Probably exclude

3 - Unsure

4 - Probably include

5 - Definitely include

Any comments on item 1.6?

1.7 Data shifts

For longitudinal datasets or datasets with versions, dataset documentation should describe any changes over time relating to the population, medical practice, or how data were collected, which may contribute to data shifts.

0:00 / 0:30

How did this item perform in round 1?

Original wording: [1.6] Data shifts. *For longitudinal datasets or datasets with versions, describe any known or suspected changes over time relating to the population, medical practice, or how data were collected, which may contribute to data shifts.*

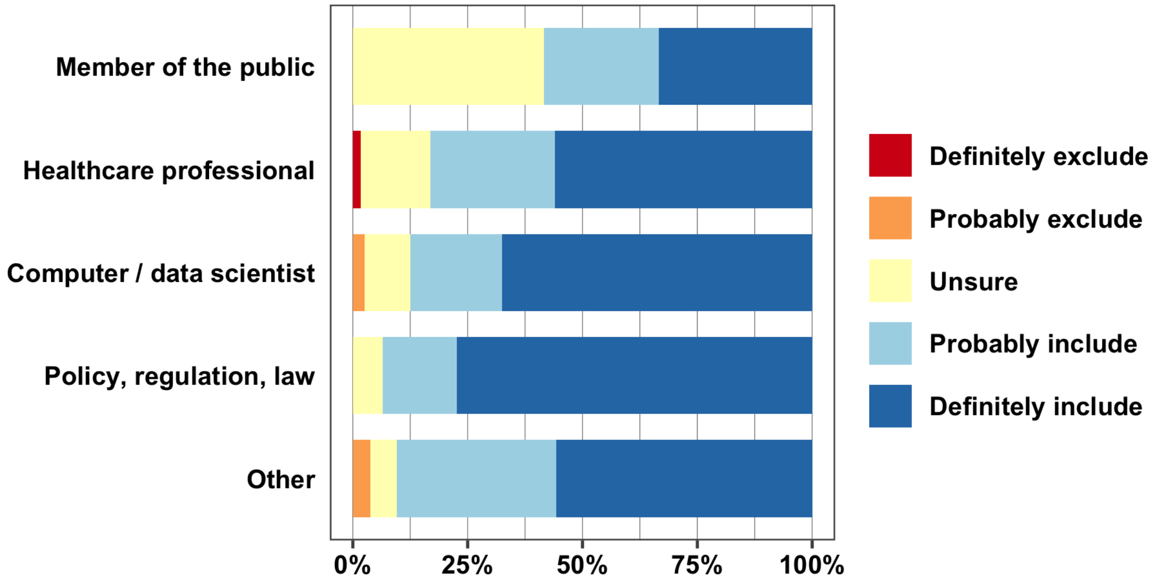

Round 1 item 1.6 (original version)

*Please vote on the **new** version of item 1.7 (highlighted in blue above).*

1 - Definitely exclude

○

2 - Probably exclude

○

3 - Unsure

○

4 - Probably include

○

5 - Definitely include

○

Any comments on item 1.7?

Documentation: Recording of attributes of individuals

We recommend you complete the survey in one sitting, but if you need to pause and come back your responses should be saved as long as you use the same device as before. This will only work if you have cookies enabled on your device and you're not using a private browsing mode.

RECORDING OF ATTRIBUTES OF INDIVIDUALS

1.8 Composition of populations

Dataset documentation should:

- Summarise the populations present in the dataset. The choice of which populations to describe, and the choice of grouping/categorisation should

be explained.

- Highlight known missing population groups within the dataset and the reason for their missingness.

0:00 / 0:59

How did this item perform in round 1?

Original wording: Composition of populations. *Summarise the relevant populations present in the dataset. Defining 'relevant populations' should be contextualised according to this dataset's specific use cases and contexts, where possible. Certain attributes (including age, gender identity, sex, race, ethnicity, socioeconomic status) should always be documented, due to known associations with health outcomes and interactions with wider social factors. If data on these particular attributes are missing this should be stated. Highlight known missing population groups within the dataset and the reason for their missingness.*

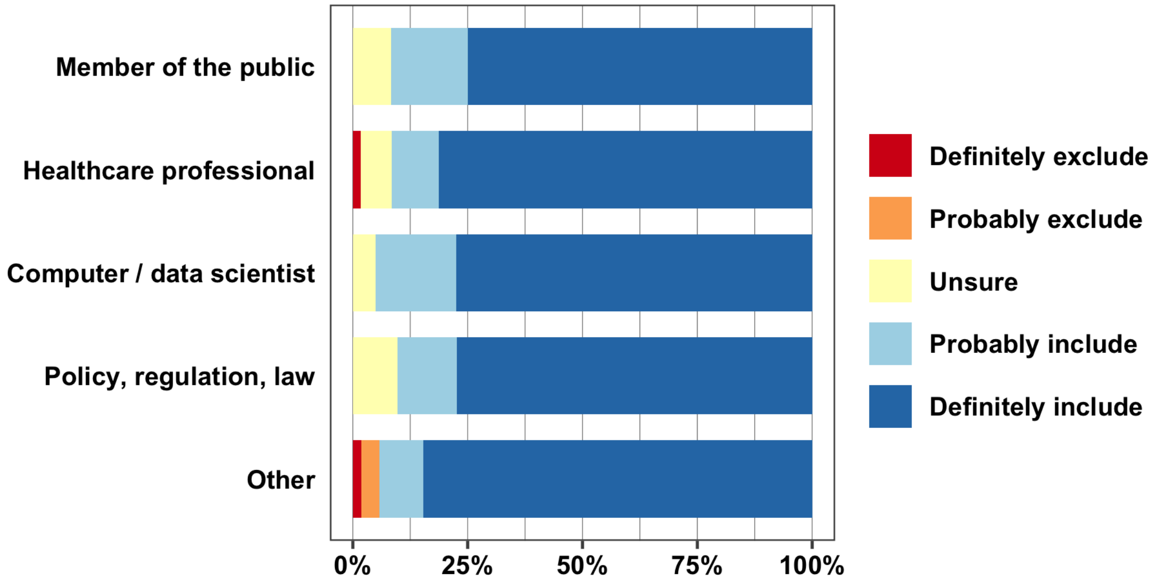

Round 1 item 1.8 (original version)

Please vote on the ***new*** *version of item 1.8 (highlighted in blue above).*

1 - Definitely exclude

2 - Probably exclude

3 - Unsure

4 - Probably include

5 - Definitely include

Any comments on item 1.8?

1.9 Recording of attributes of individuals

Dataset documentation should:

- Describe how and why attributes were collected in the dataset (self-reported by participants, imputed, linked from other datasets), and whether this information is available at the individual or aggregate level.
- Explain whether attributes have been coded, condensed or modified, stating how and why this was done.
- Highlight the proportion of attributes recorded as 'unknown' or 'other', and if possible explain the reasons why.

0:00 / 0:52

How did this item perform in round 1?

***Original wording: Recording of attributes of individuals.** Describe how attributes were collected (self-reported, imputed, linked through other datasets), whether this information is available at the individual or aggregate level, and whether data missingness is different across groups. Explain whether attributes have been coded, condensed or modified, stating how and why this was done.*

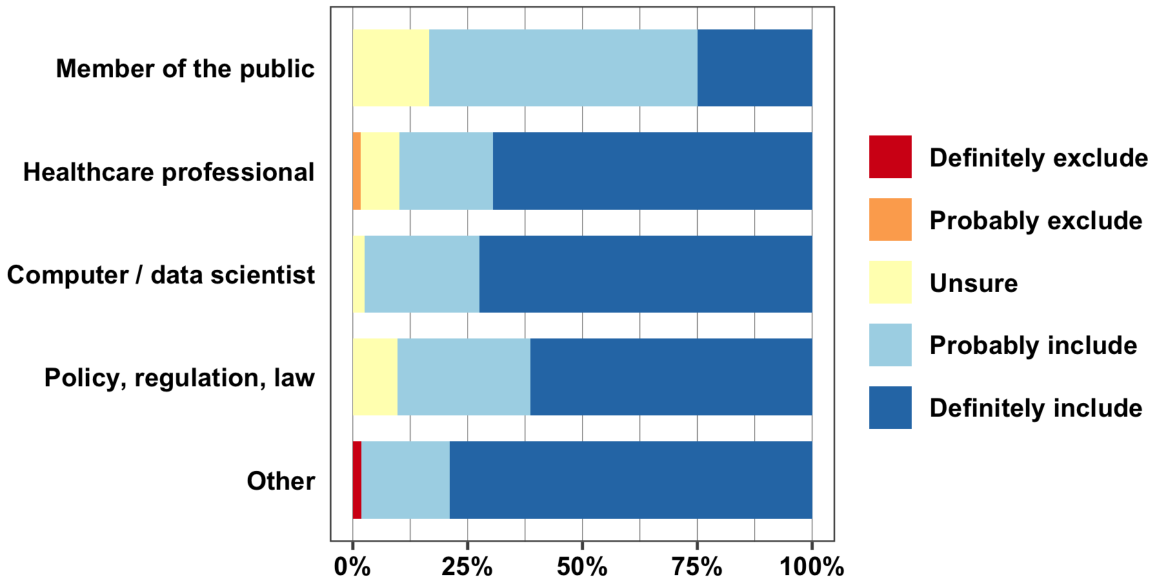

Round 1 item 1.9 (original version)

*Please vote on the **new** version of item 1.9 (highlighted in blue above).*

1 - Definitely exclude

2 - Probably exclude

3 - Unsure

4 - Probably include

5 - Definitely include

Any comments on item 1.9?

1.10 Groups at particular risk of harm

Dataset documentation should:

- Always include data (when available) on certain attributes (including age, gender identity, sex, race, ethnicity, socioeconomic status, and sexual orientation) should always be documented, due to known associations with health outcomes and interactions with wider social factors. If including these data may place individuals at risk of identification or endanger them, these data should instead be provided at aggregate level for the whole dataset. If data on these particular attributes are missing, this should be stated.
- Highlight the presence of any vulnerable population groups in this dataset, with consideration of vulnerabilities that are both universal (e.g., children, people with severe disabilities, displaced persons) and those that are specific to the site of data collection (e.g, marginalised religious or caste groups, sexual orientation and gender identity groups).

0:00 / 1:21

How did this item perform in round 1?

Original wording: Groups experiencing vulnerabilities. *Highlight the presence of any vulnerable population groups in this dataset, with consideration of both vulnerabilities that are universal (e.g., children, people with severe disabilities, displaced persons) and those that are specific to the site of data collection (e.g, marginalised religious or caste groups, sexual orientation and gender identity groups).*

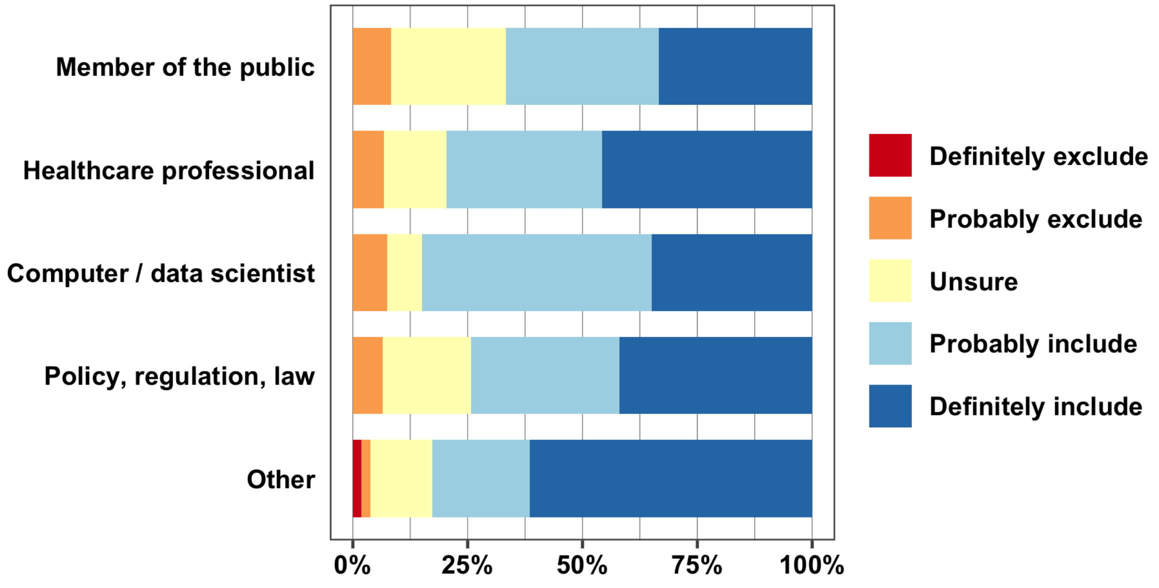

Round 1 item 1.10 (original version)

*Please vote on the **new** version of item 1.10 (highlighted in blue above).*

1 - Definitely exclude

2 - Probably exclude

3 - Unsure

4 - Probably include

5 - Definitely include

Any comments on item 1.10?

Documentation: Sources of bias

We recommend you complete the survey in one sitting, but if you need to pause and come back your responses should be saved as long as you use the same device as before. This will only work if you have cookies enabled on your device and you're not using a private browsing mode.

SOURCES OF BIAS

1.11 Modifications made to the data

Dataset documentation should describe whether any data items were modified from the original source, providing the rationale for doing so and any methods used. For example, for anonymisation, to correct for imbalance, to correct errors or biases, or to enable mapping to existing data standards.

0:00 / 0:41

How did this item perform in round 1?

Original wording: Modifications made to the data. *Describe data items which were modified from the raw source and provide the rationale for doing so and the method used. For example, for anonymisation, to correct for imbalance, to correct errors or biases, mapping to existing data standards (e.g. OMOP, FHIR, DICOM).*

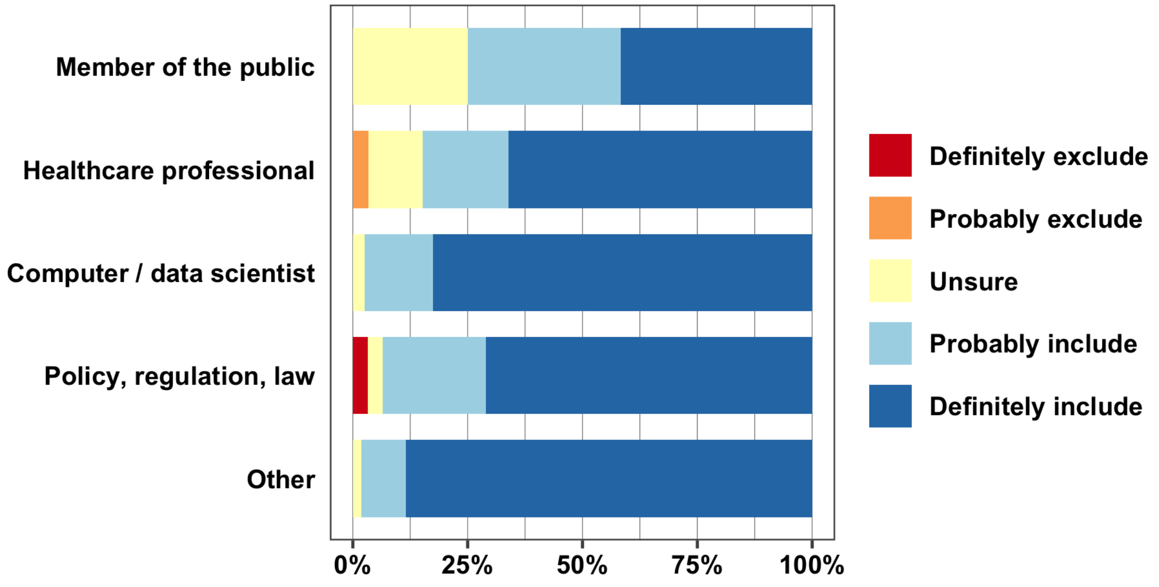

Round 1 item 1.11 (original version)

*Please vote on the **new** version of item 1.11 (highlighted in blue above).*

1 - Definitely exclude

○

2 - Probably exclude

○

3 - Unsure

○

4 - Probably include

○

5 - Definitely include

○

Any comments on item 1.11?

1.12 Limitations of the dataset

Dataset documentation should identify known or suspected sources of bias, errors or other factors that affect the dataset as a whole, which may impact its generalisability or applicability for other use.

0:00 / 0:27

How did this item perform in round 1? [moved from 1.7]

Original wording: [1.7] Limitations of the dataset *Identify known or suspected sources of bias, error or other factors that affect the dataset as a whole, which may impact its generalisability or applicability for other use.*

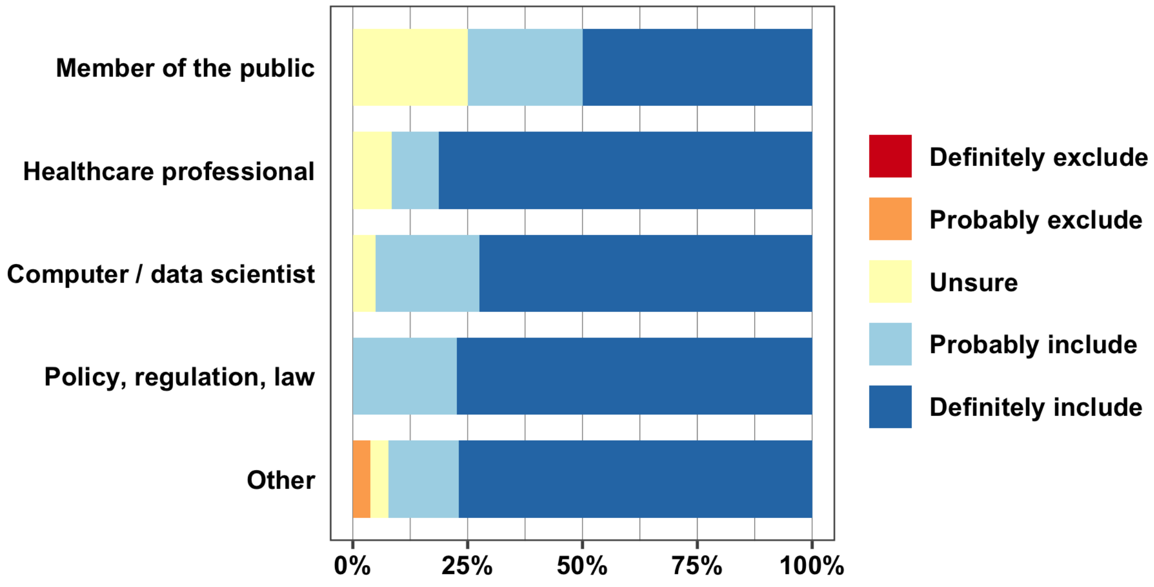

Round 1 item 1.7 (original version)

*Please vote on the **new** version of item 1.12 (highlighted in blue above).*

1 - Definitely exclude

○

2 - Probably exclude

○

3 - Unsure

○

4 - Probably include

○

5 - Definitely include

○

Any comments on item 1.12?

1.13 Missing data

Dataset documentation should describe the proportion, nature and causes of missing data (if known), particularly if there are systematic differences across relevant population groups. Documentation should also describe how missing data has been identified and handled (e.g. imputation, correction).

0:00 / 0:37

How did this item perform in round 1?

Original wording: [1.12] Missing data. *Describe the proportion, nature and causes of missing data, particularly if there are systematic differences across relevant population groups. Describe how missing data has been identified and handled (e.g. imputation, correction).*

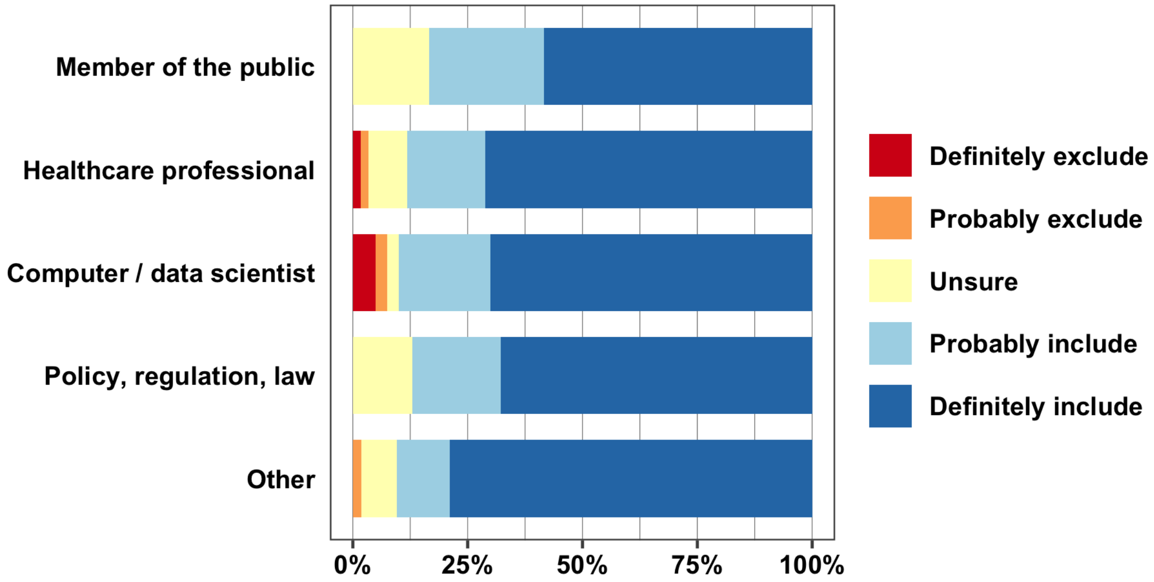

Round 1 item 1.12 (original version)

*Please vote on the **new** version of item 1.13 (highlighted in blue above).*

1 - Definitely exclude

○

2 - Probably exclude

○

3 - Unsure

○

4 - Probably include

○

5 - Definitely include

○

Any comments on item 1.13?

1.14 Errors in the data

Dataset documentation should:

- Describe how errors can be/have been identified in the data and how they have been handled (e.g. have they been removed, modified, corrected or left in the dataset).
- Provide an estimation of the proportion of errors that are present and whether they are more prevalent in some population groups than others.
- Provide possible reasons for any systematic differences in error rates across population groups within the dataset.

0:00 / 0:54

How did this item perform in round 1?

Original wording: [1.13] Errors in the data. *Describe how errors can be/have been identified in the data and how they have been handled (e.g. have they been removed, modified, corrected or left in the dataset). Provide an estimation of the proportion of errors that are present and whether they are more prevalent in some population groups than others. Provide possible reasons for any systematic differences in error rates across population groups within the dataset.*

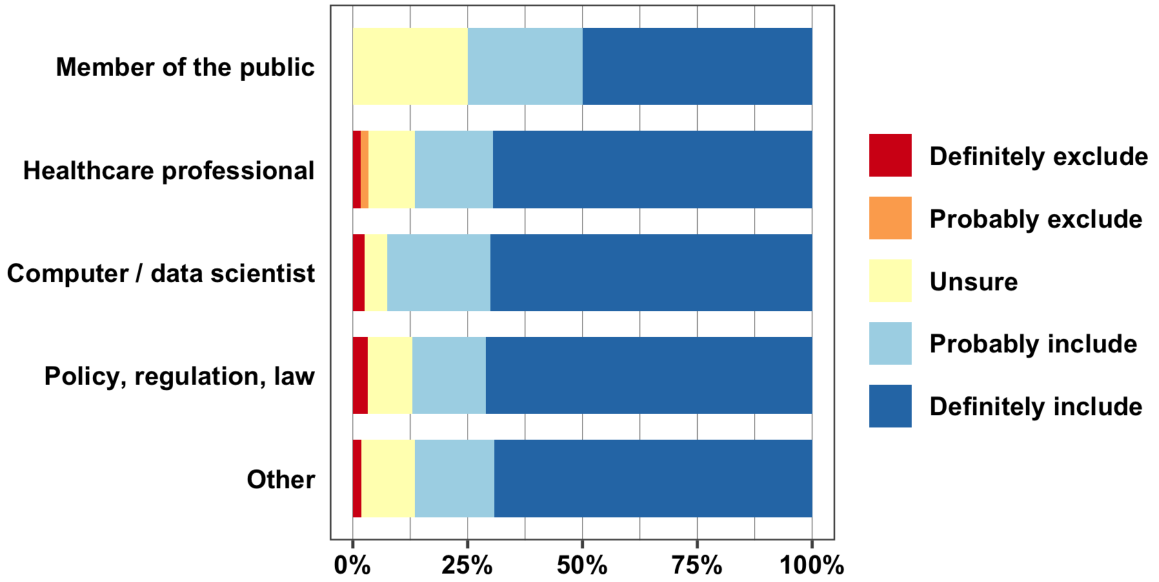

Round 1 item 1.13 (original version)

*Please vote on the **new** version of item 1.14 (highlighted in blue above).*

1 - Definitely exclude

2 - Probably exclude

3 - Unsure

4 - Probably include

5 - Definitely include

Any comments on item 1.14?

1.15 Known or potential bias in data generation

Dataset documentation should:

- Describe how bias may be introduced by the acquisition and processing of data within the dataset, for example from the use of devices, sensors and software.
- Highlight any known or potential differences in data acquired across different population groups, or any uncertainty in performance within population groups.
- Describe any attempts to mitigate these biases.

0:00 / 0:49

How did this item perform in round 1?

Original wording: [1.14] Known or potential bias in data generation. *Describe how bias may be introduced by the acquisition and processing of data within the dataset, for example from the use of devices, sensors and software. Highlight any known or potential differences in data acquired across different population groups, or any uncertainty in performance within population groups.*

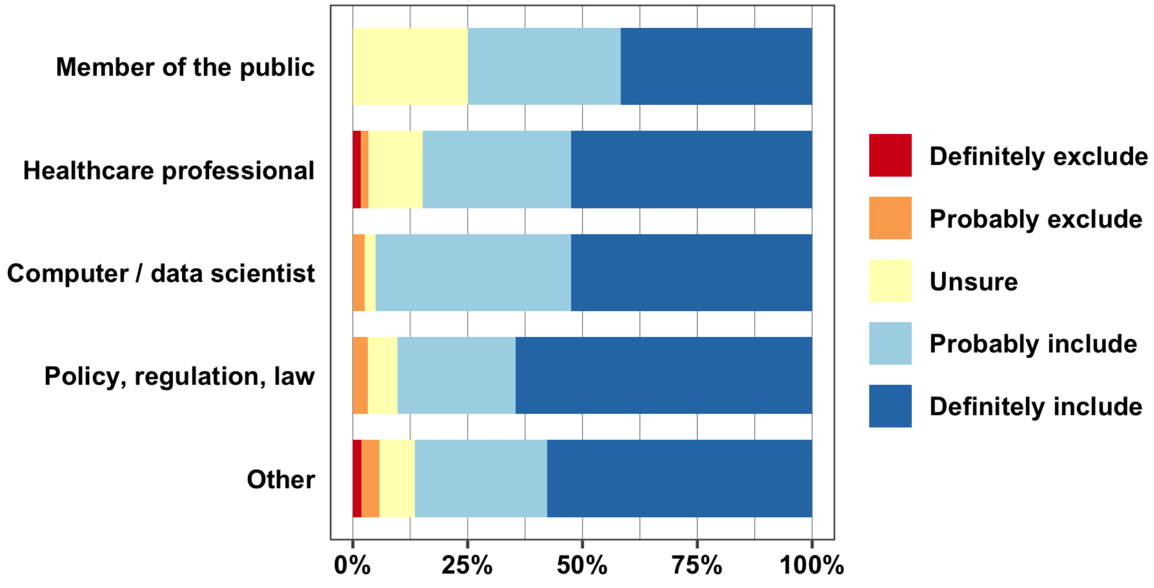

Round 1 item 1.14 (original version)

*Please vote on the **new** version of item 1.15 (highlighted in blue above).*

1 - Definitely exclude

2 - Probably exclude

3 - Unsure

4 - Probably include

5 - Definitely include

Any comments on item 1.15?

1.16 Known or potential bias in data collection

Dataset documentation should:

- Identify areas where bias may have been introduced into the data collection process. For example: only collecting data from one geographical area, context regarding healthcare coverage and accessibility, only using questionnaires in English.
- Describe any attempts to mitigate these biases.

0:00 / 0:38

How did this item perform in round 1?

Original wording: [1.15] Known or potential bias in data collection. *Identify areas where bias may have been introduced into the data collection process. For example, only collecting data from one geographical area, only using questionnaires in English.*

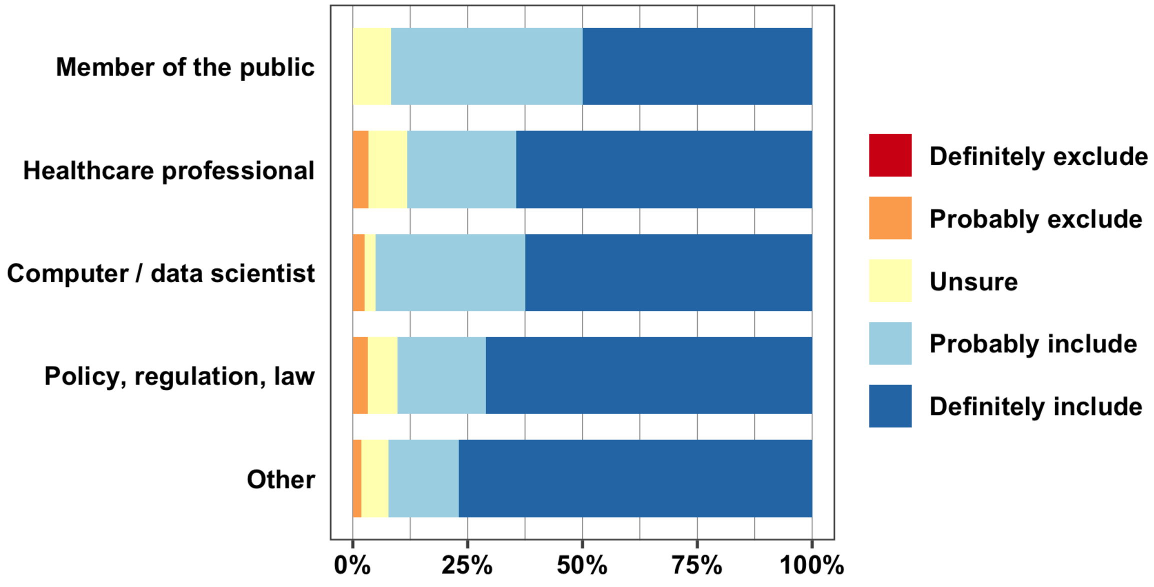

Round 1 item 1.15 (original version)

*Please vote on the **new** version of item 1.16 (highlighted in blue above).*

1 - Definitely exclude

2 - Probably exclude

3 - Unsure

4 - Probably include

5 - Definitely include

Any comments on item 1.16?

1.17 Known or potential bias in data labels

Dataset documentation should:

- Provide a description of any data labels, including who decided what labels to include, what they were called, and how they were generated.
- Highlight labels that are at high risk of bias. For example, where label generation was at the discretion of individuals, where known biases in labelling behaviour has been evidenced previously, or in the use of proxy variables (e.g., healthcare costs as a proxy of healthcare needs).
- Describe any attempts to mitigate these biases.

0:00 / 1:01

How did this item perform in round 1?

***Original wording: [1.16] Known or potential bias in data labels** Provide a description of any data labels, including who decided what labels to include, what they were called, and how they were generated. Highlight labels that are at high risk of bias. For example, where label generation was at the discretion of individuals, where known biases in labelling behaviour has been evidenced previously, or in the use of proxy variables (e.g., healthcare costs as a proxy of healthcare needs).*

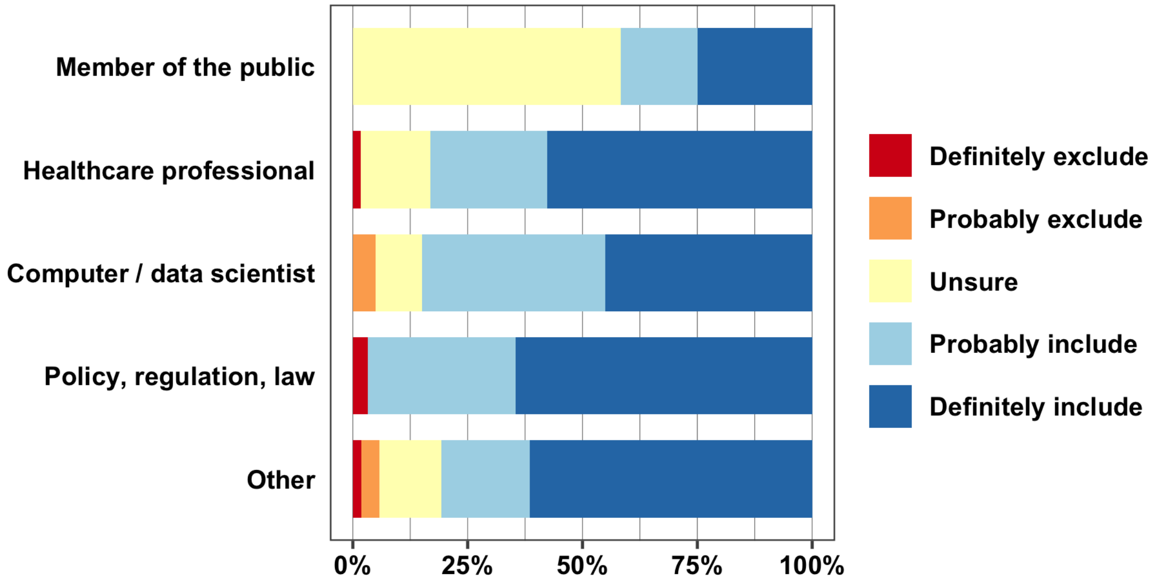

Round 1 item 1.16 (original version)

*Please vote on the **new** version of item 1.17 (highlighted in blue above).*

1 - Definitely exclude

○

2 - Probably exclude

○

3 - Unsure

○

4 - Probably include

○

5 - Definitely include

○

Any comments on item 1.17?

Documentation: Ethics and data governance

We recommend you complete the survey in one sitting, but if you need to pause and come back your responses should be saved as long as you use the same device as before. This will only work if you have cookies enabled on your device and you're not using a private browsing mode.

ETHICS AND DATA GOVERNANCE

1.18 Ethics, governance, and quality assurance

Dataset curators should describe in their documentation whether data protection laws specific to their jurisdiction have been adhered to. Dataset documentation should also:

- Describe measures taken to protect identities of individuals.
- Describe permissions obtained to enable dataset curation, and details of the governance of the dataset.
- Provide references to institutional review board/ethical committee review (or equivalent, as appropriate).
- Reference any standards (e.g. ISO, FAIR) which have been adhered to.

0:00 / 0:57

How did this item perform in round 1?

***Original wording: [1.17] Ethics and data governance** Describe the consent process for inclusion in the dataset, measures taken to protect identities of the individuals, permissions and governance of the dataset, and provide references to institutional review board/ethical committee review. Reference any standards (e.g. ISO, FAIR) which have been adhered to.*

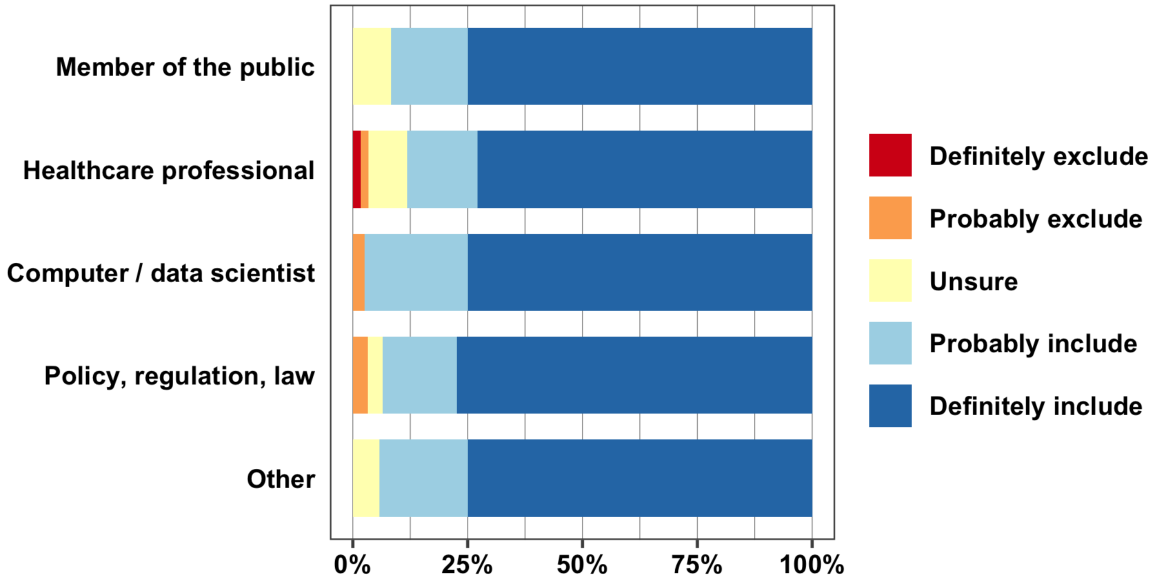

Round 1 item 1.17 (original version)

*Please vote on the **new** version of item 1.18 (highlighted in blue above).*

1 - Definitely exclude

2 - Probably exclude

3 - Unsure

4 - Probably include

5 - Definitely include

Any comments on item 1.18?

1.19 Patient and public involvement and engagement

Dataset documentation should:

- Describe the role of advisory boards and patient and public involvement and engagement groups in the dataset curation.
- Provide information on efforts to share data and findings with those who contributed to the dataset and any feedback gathered from participants that is relevant to data interpretation.

0:00 / 0:42

How did this item perform in round 1?

Original wording: [1.18] Patient and public involvement and engagement.

Describe the role of advisory boards and patient and public involvement and engagement groups in the dataset curation. Provide information on efforts to share data and findings with those who contributed to the dataset and any feedback that was gathered from participants that is relevant to data interpretation.

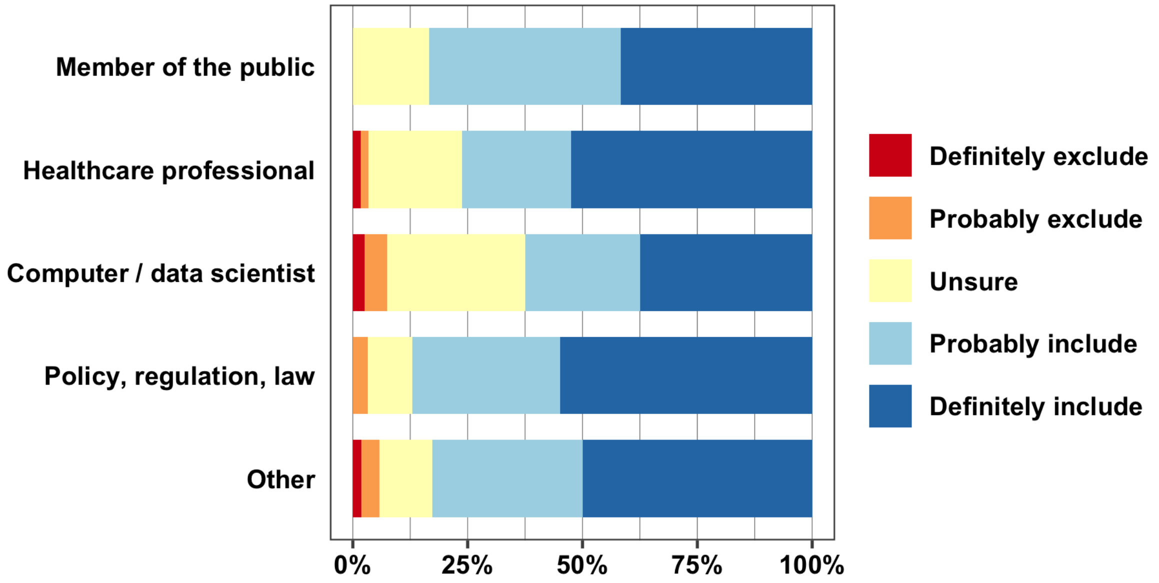

Round 1 item 1.18 (original version)

*Please vote on the **new** version of item 1.19 (highlighted in blue above).*

1 - Definitely exclude

○

2 - Probably exclude

○

3 - Unsure

○

4 - Probably include

○

5 - Definitely include

○

Any comments on item 1.19?

1.20 Bias and impact assessments

If a formal assessment of bias, fairness or societal impact has been previously conducted on the dataset, dataset documentation should provide the assessment and results. This may include algorithmic impact assessments (AIAs), data protection impact assessments (DPIAs), equality impact assessments, documentation tools, risk of bias assessments or automated toolkits.

0:00 / 0:52

How did this item perform in round 1?

Original wording: [1.19] Bias and impact assessments. *If a formal assessment of bias, fairness or societal impact has been previously conducted on the dataset, please provide the assessment and results. This may include algorithmic impact assessments (AIAs), data protection impact assessments (DPIAs), equality impact assessments, documentation tools, risk of bias assessments or automated toolkits.*

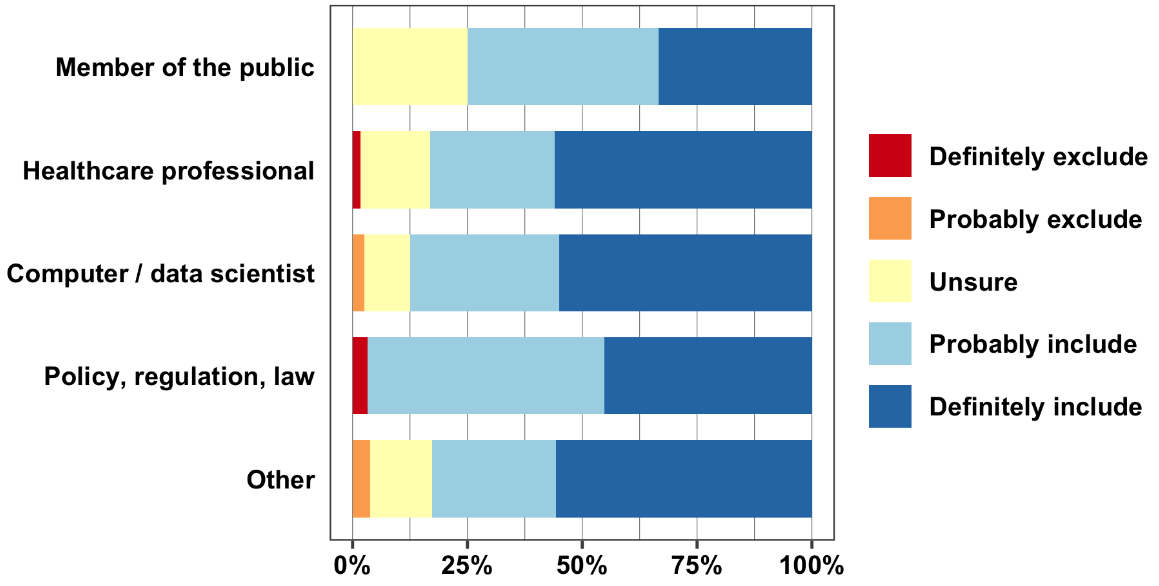

Round 1 item 1.19 (original version)

Please vote on the ***new*** *version of item 1.20 (highlighted in blue above).*

1 - Definitely exclude

2 - Probably exclude

3 - Unsure

○

4 - Probably include

○

5 - Definitely include

○

Any comments on item 1.20?

Process items - part 1 of 2

We recommend you complete the survey in one sitting, but if you need to pause and come back your responses should be saved as long as you use the same device as before. This will only work if you have cookies enabled on your device and you're not using a private browsing mode.

2 - Dataset Process Standards

0:00 / 1:24

The primary purpose of ***Dataset Process Standards*** is to promote best practice for how datasets should be used throughout the AI Health Technology lifecycle, and promote documentation of how risks to relevant subgroups have been identified and mitigated.

The Dataset Process Standards are primarily for Data Users, as they require context and should be considered with a specific use-case in mind. The concept of **contextualised subgroups of interest** is introduced: subgroups with shared attributes, identified as being relevant and important for the use case, and where they

are known to have worse health outcomes or are subject to other systems driving health inequity related to the use case.

The items are written so that they can be operationalisable within the context of existing medical device regulation, leaning upon existing requirements such as intended use statements, evidentiary requirements (literature review, clinical evaluation plan) and post-market surveillance and post-market clinical follow-up. Where certain items may not be relevant, this is specifically called out in the text. Unlike the documentation requirements above, a 'NA' or 'not done' response is not considered acceptable for any of these (unless, as previously stated, a 'not applicable' option is specifically called out).

Please indicate whether we should include or exclude each of the below delphi items. There is a space to write comments or suggest improvements below each item.

2.1 Provide sufficient information about dataset(s) to allow traceability and auditability

Datasets used in the lifecycle of AI health technologies should be accompanied by documentation which conforms to items 1.1 - 1.20, enabling audit against these standards.

0:00 / 0:27

How did this item perform in round 1?

Original wording: *Full documentation of datasets used in the lifecycle of AI health technologies should be provided to enable audit against these standards.*

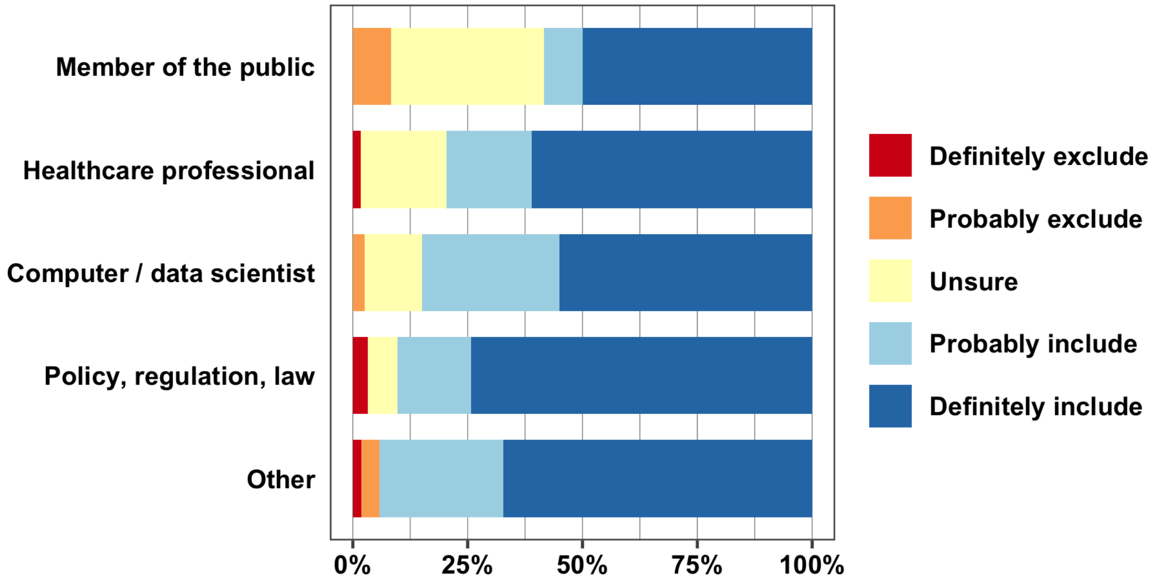

Round 1 item 2.1 (original version)

*Please vote on the **new** version of item 2.1 (highlighted in blue above).*

1 - Definitely exclude

2 - Probably exclude

3 - Unsure

4 - Probably include

5 - Definitely include

Any comments on item 2.1?

2.2 Identify contextualised subgroups of interest who are particularly at risk of harm from the AI health technology under development

Data Users should identify contextualised subgroups of interest in advance: these are subgroups with shared attributes, identified as being relevant and important for the use case, and where they are known to have worse health outcomes or are subject to other systems driving health inequity related to the use case. Contextualised subgroups of interest may be discovered via multiple sources, including literature review, evidence from the development or use of similar AI health technologies, consultation with experts in health inequity, clinical practice, etc.

0:00 / 1:13

How did this item perform in round 1? [combined original items 2.2 and 2.3]

Original wording:

2.2 *Data Users should identify contextualised subgroups of interest in advance: these are subgroups with shared attributes, identified as being relevant and important for the use case, and where they are known to have worse health outcomes or are subject to other systems driving health inequity related to the use case.*

2.3 *Data Users should identify and document in the literature review evidence of discrepant performance across any subgroups in similar devices.*

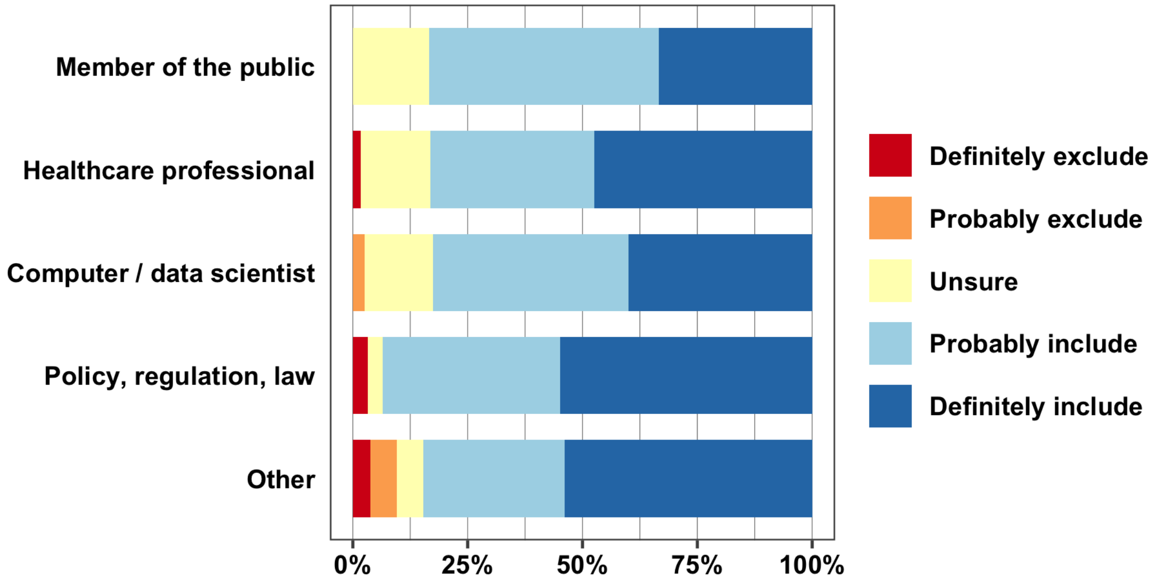

Round 1 item 2.2 (original version)

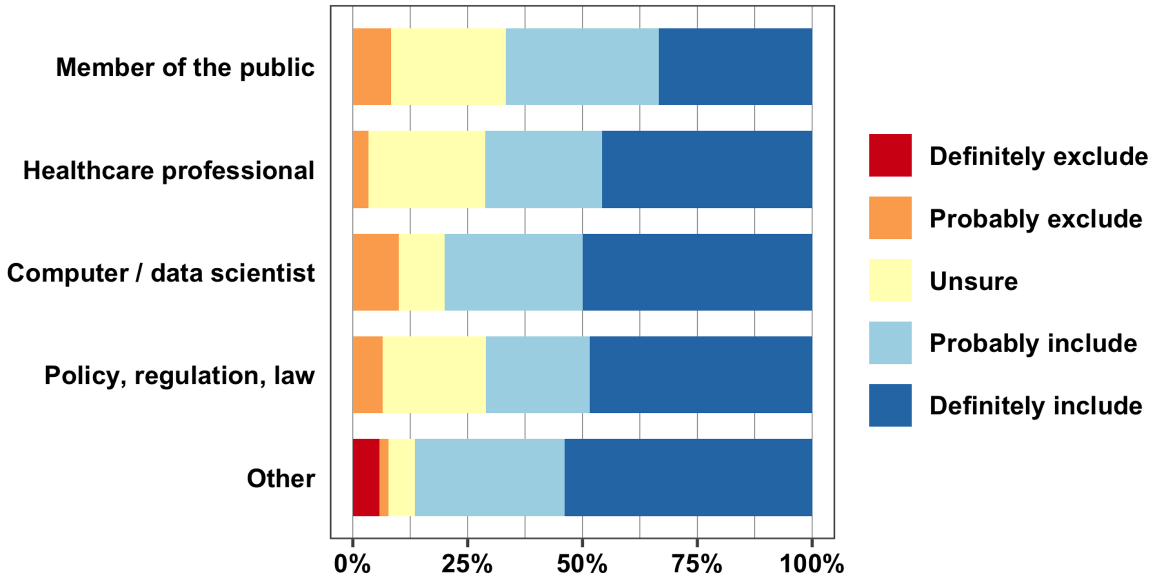

Round 1 item 2.3 (original version)

*Please vote on the **new** version of item 2.2 (highlighted in blue above).*

1 - Definitely exclude

○

2 - Probably exclude

○

3 - Unsure

○

4 - Probably include

○

5 - Definitely include

○

Any comments on item 2.2?

2.3 Use appropriate datasets to support the intended use population and intended purpose of the AI health technology

The intended use population should be adequately represented in the datasets used in an AI health technology. The contextualised subgroups of interest should also be included where possible, and if not, explicitly stated. Areas of under-representation should be identified and transparently reported.

0:00 / 0:00

How did this item perform in round 1? [moved from 2.6]

Original wording: *The intended use population of the AI Health Technology should be adequately represented in the training and test datasets for an AI Health Technology. The contextualised subgroups of interest should also be included where possible, and if not, explicitly stated. Areas of under-representation should be identified and transparently reported.*

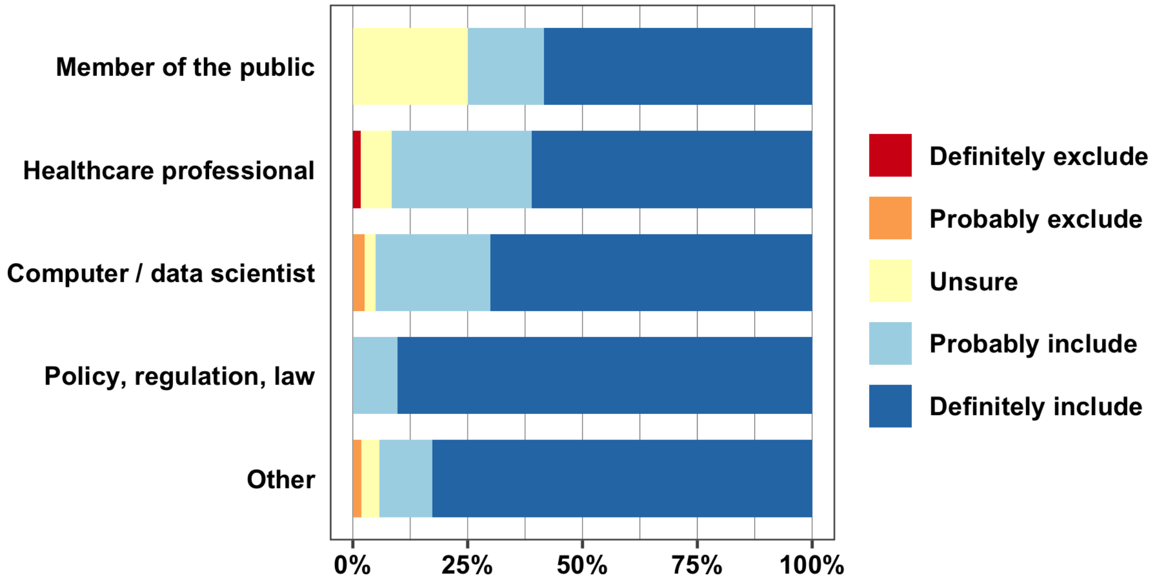

Round 1 item 2.6 (original version)

*Please vote on the **new** version of item 2.3 (highlighted in blue above).*

1 - Definitely exclude

2 - Probably exclude

3 - Unsure

4 - Probably include

5 - Definitely include

Any comments on item 2.3?

2.4 Identify discrepant performance of the AI health technology for contextualised subgroups of interest

Data Users should:

- Report performance of the AI health technology for contextualised subgroups of interest, identified in 2.3.
- Compare this performance for contextualised subgroups of interest against aggregate performance in the overall study population.
- Report performance of the AI health technology for subgroup(s) who have the best pre-existing health outcomes in this clinical area, and compare this to performance for contextualised subgroups of interest.

0:00 / 0:00

How did this item perform in round 1?

Original wording: *Having identified contextualised subgroups of interest, Data Users should report evaluation results of the AI Health Technology within those subgroups, in addition to aggregate results of evaluation.*

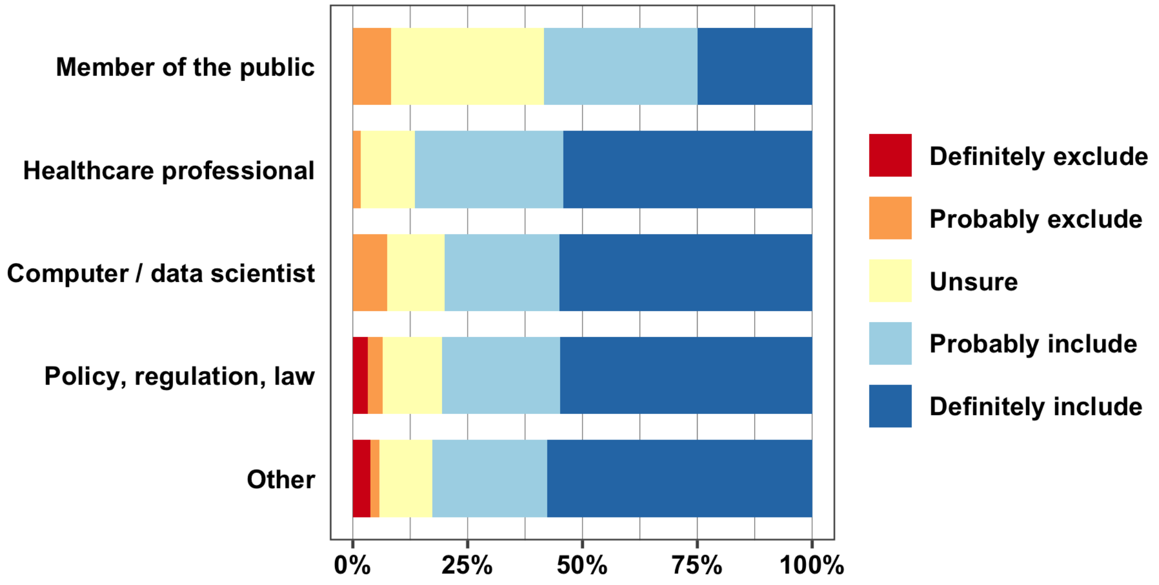

Round 1 item 2.4 (original version)

*Please vote on the **new** version of item 2.4 (highlighted in blue above).*

1 - Definitely exclude

○

2 - Probably exclude

○

3 - Unsure

○

4 - Probably include

○

5 - Definitely include

○

Any comments on item 2.4?

2.5 Evaluate performance of the AI health technology for subgroups experiencing vulnerability

If not already addressed by 2.4, Data Users should report evaluation results across certain attributes (including age, gender identity, sex, race, ethnicity, socioeconomic status and sexual orientation), due to known associations with health outcomes and interactions with wider social factors. This may not always be possible or appropriate, in which case the reasons for not doing so should be documented.

0:00 / 0:00

How did this item perform in round 1?

Original wording: *Data Users should also report evaluation results across certain attributes (including age, gender, sex, race, ethnicity, socioeconomic status), due to known associations with health outcomes and interactions with wider social factors. This may not always be possible or appropriate, in which case the reasons for not doing so should be documented.*

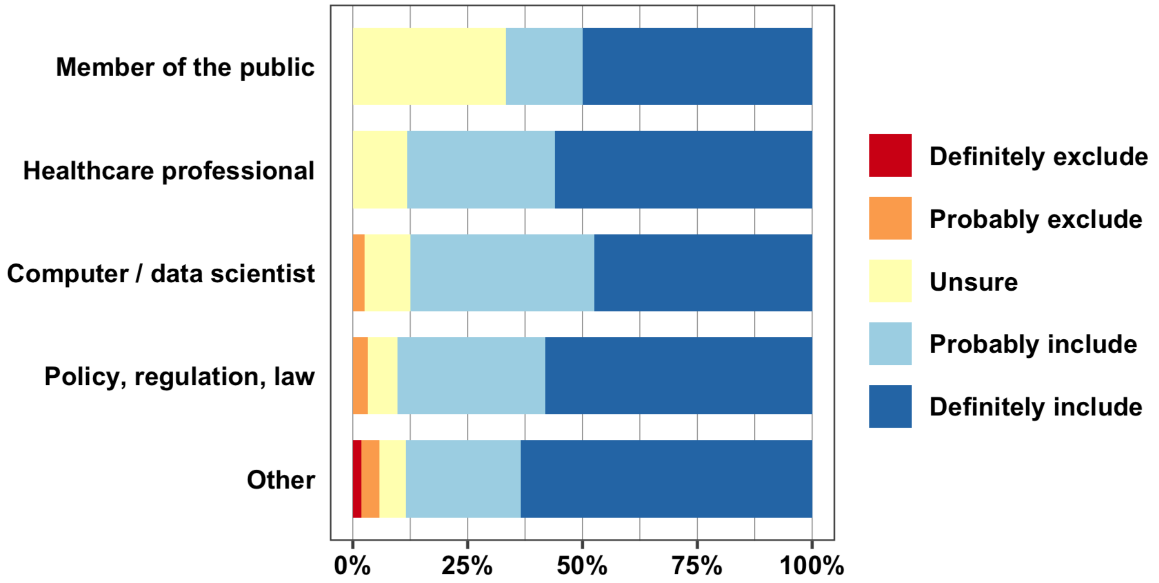

Round 1 item 2.5 (original version)

*Please vote on the **new** version of item 2.5 (highlighted in blue above).*

1 - Definitely exclude ○

2 - Probably exclude ○

3 - Unsure ○

4 - Probably include ○

5 - Definitely include ○

Any comments on item 2.5?

Process items - part 2 of 2

We recommend you complete the survey in one sitting, but if you need to pause and come back your responses should be saved as long as you use the same device as before. This will only work if you have cookies enabled on your device and you're not using a private browsing mode.

ACKNOWLEDGE KNOWN BIASES AND LIMITATIONS OF DATASET(S) AND ANY IMPLICATIONS ON THE INTENDED USE OF THE AI HEALTH TECHNOLOGY

2.6 Report limitations of datasets used and any implications on the AI health technology

Data Users should report limitations of the dataset and the implications on the target AI health technology. Data Users should investigate whether limitations are systematically different across relevant population subgroups, including those categorised as ‘unknown’ or ‘other’, and report differences which could result in worse performance on the AI Health Technology across groups.

0:00 / 0:00

How did this item perform in round 1?

Original wording: [2.7] *Data Users should report limitations of the dataset and the implications on the AI Health Technology. Data Users should investigate whether limitations are systematically different across relevant population subgroups, including those categorised as ‘unknown’ or ‘other’, and report differences which could result in worse performance on the AI Health Technology across groups.*

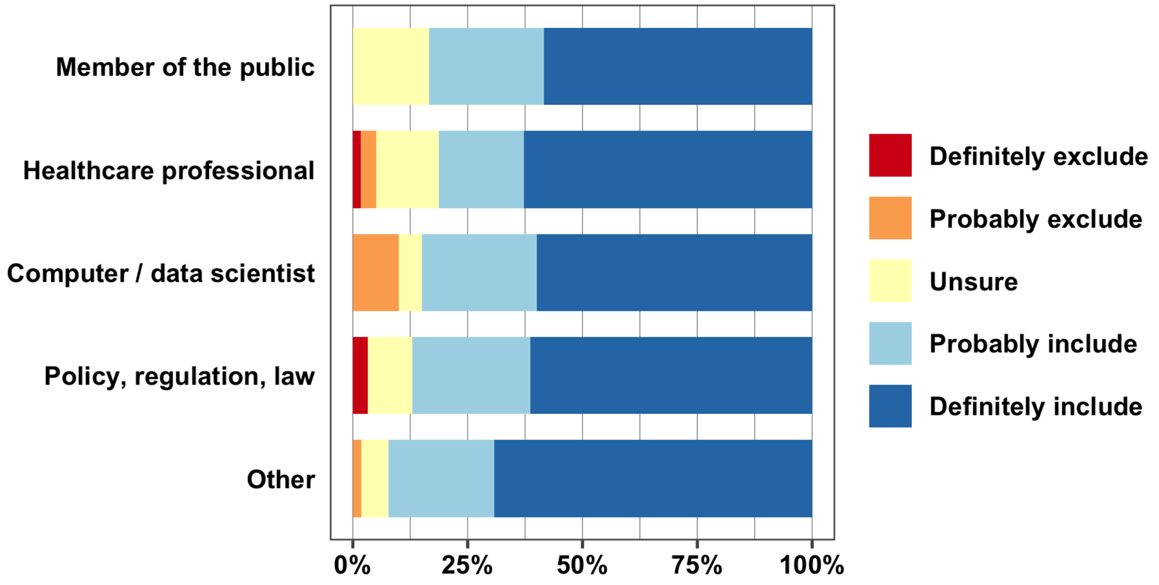

Round 1 item 2.7 (original version)

*Please vote on the **new** version of item 2.6 (highlighted in blue above).*

1 - Definitely exclude

2 - Probably exclude

3 - Unsure

4 - Probably include

5 - Definitely include

Any comments on item 2.6?

2.7 Report differences between the intended purposes of the AI health technology and datasets used during development, including the implications of discordance.

State any intended purposes of datasets used (item 1.3), and how these differ from the intended purpose of the AI health technology (item 2.3). State implications of any discordance and provide justification regarding the suitability of the dataset(s), including assumptions made and aspects of the dataset(s) which are not directly applicable.

0:00 / 0:00

How did this item perform in round 1?

Original wording: [2.8] *State any intended purposes of the dataset, and how this differs from the intended use of the AI Health Technology. State implications of any discordance and provide justification regarding the suitability of the dataset, including assumptions made and aspects of the dataset which are not directly applicable.*

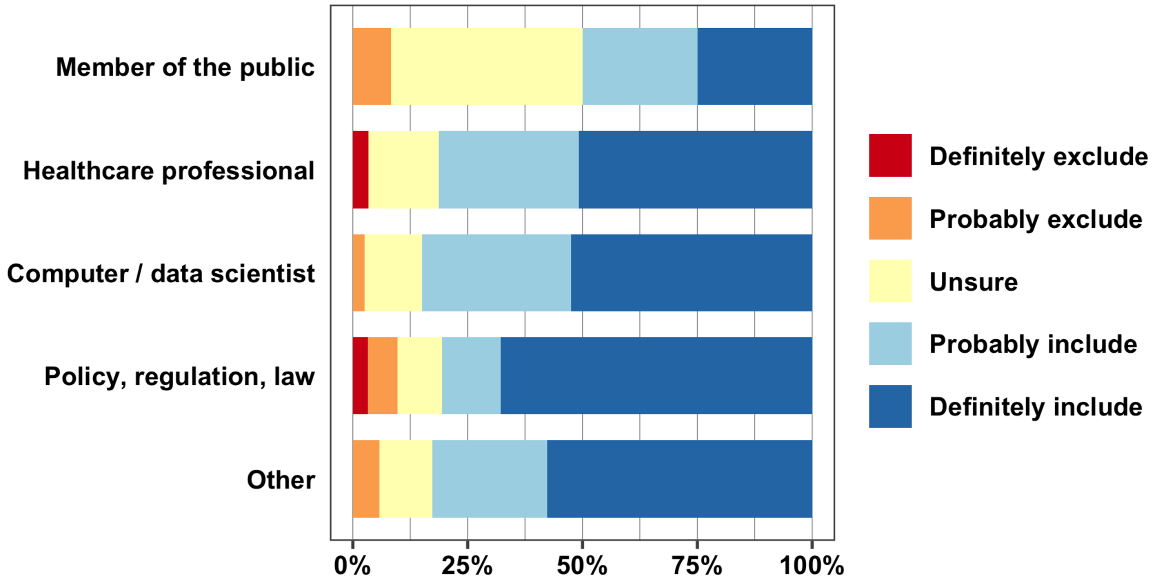

Round 1 item 2.8 (original version)

*Please vote on the **new** version of item 2.7 (highlighted in blue above).*

1 - Definitely exclude

○

2 - Probably exclude

○

3 - Unsure

○

4 - Probably include

○

5 - Definitely include

○

Any comments on item 2.7?

2.8 Report level of uncertainty for performance in subgroups when sample size is insufficient

Should sample size not be achieved in minority and/or intersectional population groups, Data Users should report the level of uncertainty for performance in these subgroups (e.g. with confidence intervals). Where this may suggest additional risk, describe whether mitigation plans are in place to avoid harm to these groups.

0:00 / 0:00

How did this item perform in round 1?

Original wording: [2.9] *In the case of minority and intersectional population groups, achieving sufficient sample size may not be possible. Data Users should report the level of uncertainty for performance in these subgroups (e.g. with confidence intervals).*

Where this may confer additional risk, mitigation plans should be in place to avoid harm to these groups.

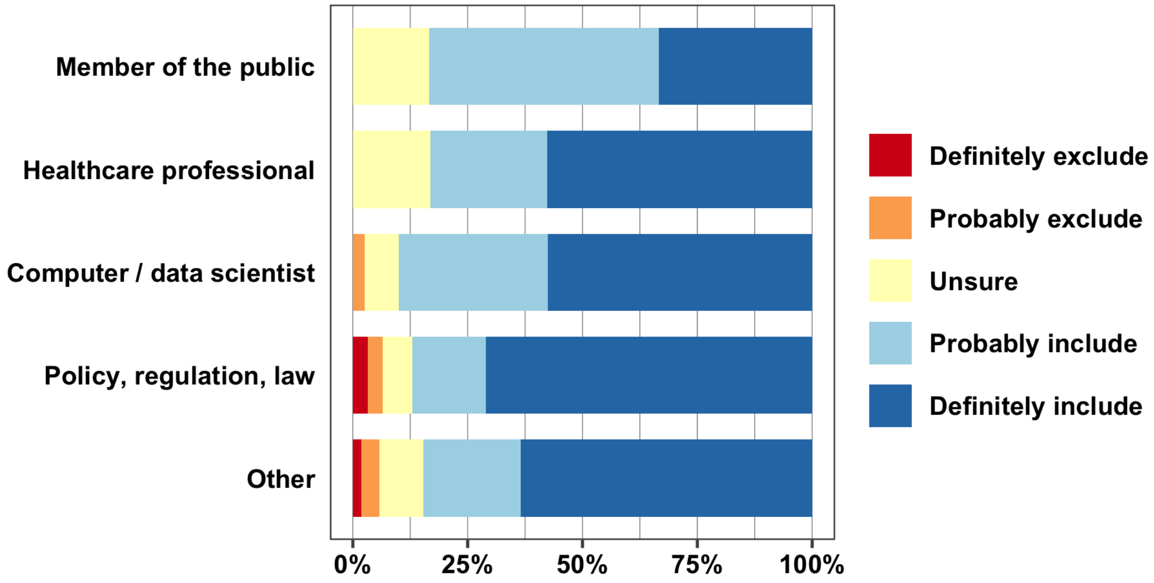

Round 1 item 2.9 (original version)

Please vote on the ***new*** *version of item 2.8 (highlighted in blue above).*

1 - Definitely exclude

2 - Probably exclude

3 - Unsure

4 - Probably include

5 - Definitely include

Any comments on item 2.8?

2.9 Report findings of pre-existing dataset assessments

Data Users should review any pre-existing assessments of the datasets which are available (e.g. algorithmic impact assessments, equality impact assessments, data protection impact assessments, datasheets, healthsheets) and report how the findings may translate to harm for subgroups within the intended use population.

0:00 / 0:00

How did this item perform in round 1?

***Original wording: [2.10]** Data Users should review pre-existing assessments of the datasets (e.g. algorithmic impact assessments, equality impact assessments, data protection impact assessments, datasheets, healthsheets) and report how the findings may translate to harm for subgroups within the intended use population.*

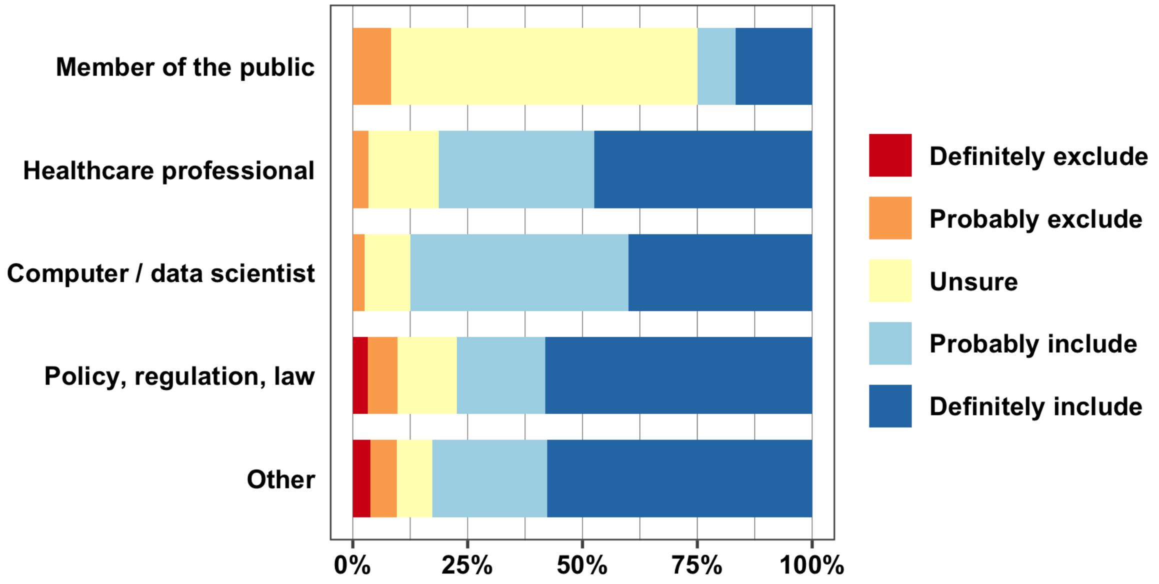

Round 1 item 2.10 (original version)

*Please vote on the **new** version of item 2.9 (highlighted in blue above).*

1 - Definitely exclude

○

2 - Probably exclude

○

3 - Unsure

○

4 - Probably include

○

5 - Definitely include

○

Any comments on item 2.9?

2.10 Address uncertainties and risks with mitigation plans

Where Data Users have identified uncertainty or potentially variable performance in subgroups, any clinical implications resulting from these findings must be clearly stated and reported as risks. The Data User should document plans to monitor these risks as part of the post market clinical follow-up and post market surveillance.

0:00 / 0:00

How did this item perform in round 1?

Original wording: [2.11] *Where Data Users have identified uncertainty or potentially variable performance in subgroups, this should be identified and reported as a risk. If*

the Data User is a manufacturer of an AI health technology, risks must be monitored as part of the post market clinical follow-up and post market surveillance plans.

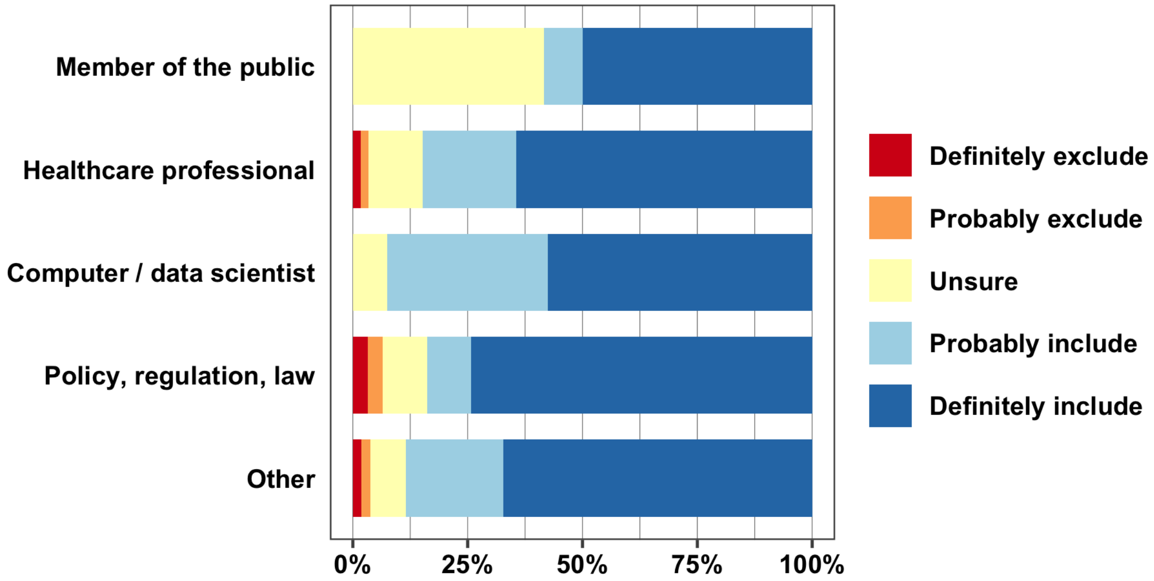

Round 1 item 2.11 (original version)

*Please vote on the **new** version of item 2.10 (highlighted in blue above).*

1 - Definitely exclude ○

2 - Probably exclude ○

3 - Unsure ○

4 - Probably include ○

5 - Definitely include ○

Any comments on item 2.10?

Survey ending

We will amend the standard items contained in this survey based on your feedback.

Which of the following statements best describe you?

You can select more than one option if needed

- ☐ I'm a healthcare professional
- ☐ I'm a member of the public
- ☐ I'm a computer scientist / data scientist
- ☐ I work in policy, regulation, law, politics, or related fields
- ☐ I'm an academic researcher
- ☐ I work in social science
- ☐ Other (please state below)

Are you a dataset curator?

Data Curators are individuals or groups who are involved in the production of datasets. This includes those who generate, collect, modify, aggregate and publish datasets. It also includes individuals or groups who provide guidance or insight which influences a dataset.

- ○ Yes
- ○ No

Are you involved in creating AI / ML models for healthcare?

- ○ Yes
- ○ No

Some of the items in these standards specifically call out certain personal attributes to be reported

0:00 / 0:00

These are: age, gender identity, sex, race, ethnicity, socioeconomic status and sexual orientation.

There are many other attributes which might be important to report. The comments from delphi round 1 showed differing opinions about which should be mentioned specifically in the item, and which may be listed in the standards' explanatory text. We plan that this explanatory text will accompany the standards, and add context and clarity to each item.

For each of the below characteristics, indicate whether they should be included in the items themselves, or mentioned in the standards' accompanying documentation:

	In the items themselves	In the explanatory text
Age	○	○
Gender identity	○	○
Sex	○	○
Race	○	○
Ethnicity	○	○
Socioeconomic status	○	○
Sexual orientation	○	○
Disability	○	○
Gender reassignment	○	○
Marriage and civil partnership	○	○
Pregnancy and maternity	○	○
Religion or belief	○	○
Nationality	○	○
Ancestry	○	○
Occupation	○	○
Language	○	○

	In the items themselves	In the explanatory text
Caste	○	○
Creed	○	○

Do you have any comments about these attributes, or any others we may have missed?

Thank you for completing our survey - just a couple of final questions

0:00 / 0:00

These final questions are **voluntary**. If you'd rather not answer any or all of them you can scroll down and submit the survey.

Our standards aim to protect minoritised groups in society from potential harms associated with medical AI. To ensure we meet this aim, it's important we hear the voices of those who are minoritised. Your responses to these next questions will help us understand what types of people have responded to our survey so we can make sure our standards represent the views of as many people in society as possible.

We will not share your responses with anyone else.

We will not store your responses to these questions with your previous responses in this survey - they will be aggregated and individual responses will be redacted. For a reminder of how we will use your personal information, please click here.

What is your age?

We've used ranges here to protect your identity

- ○ I'd prefer not to answer this question
- ○ 0-17 years
- ○ 18-30 years
- ○ 30-60 years

- 60+ years

What is your race?

For some people race and ethnicity are the same thing, but for others they are different constructs. Please interpret this question in your own context - we've not provided categories to allow maximal self expression. You can leave this blank if you'd prefer.

What is your ethnicity?

We've used the UK Office for National Statistics categories here. We recognise these categories are not inclusive for all geographies and communities, so if you'd prefer to self identify there are options to write a free-text response.

If writing a self-identified ethnicity, please only enter it into one of the comments boxes. For instance - if you wanted to identify as 'African American' you could enter this into either the box titled "Any other Black / African / Caribbean background" or the box titled "Any other ethnic group" - but not into both.

- I'd prefer not to answer this question

White

- English / Welsh / Scottish / Northern Irish / British
- Irish
- Gypsy or Irish Traveller
- Any other White background (please describe in the self identification box below)

Mixed / multiple ethnic groups

- White and Black Caribbean
- White and Black African
- White and Asian

- ○ Any other Mixed / Multiple ethnic background (please describe in the self identification box below)

Asian / Asian British

- ○ Indian
- ○ Pakistani
- ○ Bangladeshi
- ○ Chinese
- ○ Any other Asian background (please describe in the self identification box below)

Black / African / Caribbean / Black British

- ○ African
- ○ Caribbean
- ○ Any other Black / African / Caribbean background (please describe in the self identification box below)

Other ethnic group

- ○ Arab
- ○ Any other ethnic group (please describe in the self identification box below)

In which country do you currently reside?

What is your sex (as assigned at birth)?

- I'd prefer not to answer this question
- Female
- Male
- Intersex
- Other

Is your gender identity the same as the sex you were assigned at birth?

- I'd prefer not to answer this question
- Yes
- No

How do you describe your sexual orientation?

- I'd prefer not to answer this question
- Homosexual
- Bisexual
- Pansexual
- Asexual
- Heterosexual
- I'd prefer to self identify (please give response below)

If you have any final comments about any of the standard items, the way we've run this study, or anything else, please write them below

Powered by Qualtrics

STANDING Together

Survey introduction & consent

STANDING Together - a project to develop STANdards for data Diversity, INclusivity and Generalisability.

Welcome to round 2b of the delphi study for the STANDING Together project

Thank you for completing the first two rounds of the STANDING Together delphi study back in October! Based on your votes and comments, we've published our draft recommendations for anyone to view and comment on. You can find them here: http://www.datadiversity.org/public-consultation

We need your help one last time - to decide if we should include an extra item covering the concepts of **thresholds** and **fairness** in these standards.

Click the blue arrow below to continue >>>

Participant Information

Study Title: Developing STANdards for data Diversity, INclusivity and Generalisability (STANDING together)

You will already have read and reviewed the participant information sheet when you completed round 1 and/or 2 of the delphi study - none of this information has changed. We do know that it can be hard to remember some of this information - if you'd like to remind yourself about the study (including how we will use any data you provide in this form) please click here to open a copy of the information sheet in your web browser.

Alternatively, you can click the play button below to listen to a recorded version of the information sheet.

0:00 / 6:24

If you have questions or concerns about any aspect of this study, or if you wish to withdraw, please contact us by email - contact@datadiversity.org

I confirm that I participated in round 1 and/or 2 of the delphi study

- ○ Yes
- ○ No

I confirm that I am willing to participate in round 2b of this study

If you select 'no' below you'll be taken to the end of the survey and no data will be recorded about you

- ○ Yes
- ○ No

I consent to information I enter into this form being stored and processed by the STANDING Together research team

To be reminded of how we will use your data:
Click here to open a copy of the participant information sheet in your browser
Click here to view the University of Birmingham data protection policies

If you select 'no' below you'll be taken to the end of the survey and no data will be recorded about you

- ○ Yes
- ○ No

Briefing

Thank you for your votes and comments in earlier rounds. We've used these to develop and publish our draft standards.

0:00 / 1:52

How we've acted on your feedback

All of the delphi items from round 2 achieved the required 75% support needed. Based on comments, we've made some adjustments to some of the items. You can read our green paper, which sets out the items in context, by clicking here (may open a new window).

Why are you running round 2b of the delphi study?

Some feedback from other collaborators has highlighted that none of our items discuss the concepts of thresholds or algorithmic fairness. These are technical terms, which we define below. **We need your help deciding if we should add a new item to consider these concepts.**

Threshold

- Healthcare AI algorithms work by calculating probabilities, which may indicate how likely it is that a disease is present. The threshold is the probability level above which we (as humans) say that a person has a particular disease. If the AI algorithm's calculated probability is below this threshold, it means the person is not labelled as having the disease.
- By setting the threshold higher or lower, developers can change the accuracy of predictions, the number of false positives, and other aspects of the algorithm's performance.

Algorithmic fairness

- Many studies have shown that AI algorithms can perform differently for certain groups of people in society. This might mean that an algorithm benefits one group over another.
- **Algorithmic fairness** involves making changes to the way an algorithm works to reduce the differences in its performance between groups of people.
- This may have unanticipated consequences, including making the algorithm work less well overall to achieve equality between groups ('levelling down').

This video gives an overview of thresholds and fairness, and explains how the green paper & consultation work:

New delphi item

Report any statistical approaches (including 'fairness methods/metrics') used to intentionally modify performance across subgroups.

Data users should document any attempts during development and evaluation of the AI health technology, which attempt to make predictions more equitable across subgroups. Describe:

• The rationale and goals for doing so.
• The methods and metrics used.
• How thresholds were set, including whether these vary between subgroups of people.

Please vote on whether this new item should be included in the standards.

1 - Definitely exclude

○

2 - Probably exclude

○

3 - Unsure

○

4 - Probably include

○

5 - Definitely include

○

Do you have any comments or suggestions to make about this new proposed item?

Please include any suggestions to improve the wording or language (if you have any)

Which of the following statements best describe you?

You can select more than one option if needed

- ☐ I'm a healthcare professional
- ☐ I'm a member of the public
- ☐ I'm a computer scientist / data scientist
- ☐ I work in policy, regulation, law, politics, or related fields
- ☐ I'm an academic researcher
- ☐ I work in social science
- ☐ Other (please state below)

Are you a dataset curator?

Data Curators are individuals or groups who are involved in the production of datasets. This includes those who generate, collect, modify, aggregate and publish datasets. It also includes individuals or groups who provide guidance or insight which influences a dataset.

- Yes
- No

Are you involved in creating AI / ML models for healthcare?

- Yes
- No

Before this survey, had you heard of thresholds (in the context of AI algorithms)?

- Yes
- No

Before this survey, had you heard of algorithmic 'fairness'?

- Yes
- No

If you have any final comments about any of the standard items, the way we've run this study, or anything else, please write them below

This is the end of round 2b of the Delphi survey. Please click the blue button below to record your responses >>>

Powered by Qualtrics
